# GrapeTree: visualization of core genomic relationships among 100,000 bacterial pathogens

**DOI:** 10.1101/gr.232397.117

**Published:** 2018-09

**Authors:** Zhemin Zhou, Nabil-Fareed Alikhan, Martin J. Sergeant, Nina Luhmann, Cátia Vaz, Alexandre P. Francisco, João André Carriço, Mark Achtman

**Affiliations:** 1 Warwick Medical School, University of Warwick, Coventry, CV4 7AL, United Kingdom;; 2 Instituto de Engenharia de Sistemas e Computadores: Investigação e Desenvolvimento (INESC-ID), 1000-029 Lisboa, Portugal;; 3 ADEETC, Instituto Superior de Engenharia de Lisboa, Instituto Politécnico de Lisboa, 1959-007 Lisboa, Portugal;; 4 Instituto Superior Técnico, Universidade de Lisboa, 1049-001 Lisboa, Portugal;; 5 Instituto de Microbiologia, Instituto de Medicina Molecular, Faculdade de Medicina, Universidade de Lisboa, 1649-004 Lisboa, Portugal

## Abstract

Current methods struggle to reconstruct and visualize the genomic relationships of large numbers of bacterial genomes. GrapeTree facilitates the analyses of large numbers of allelic profiles by a static “GrapeTree Layout” algorithm that supports interactive visualizations of large trees within a web browser window. GrapeTree also implements a novel minimum spanning tree algorithm (MSTree V2) to reconstruct genetic relationships despite high levels of missing data. GrapeTree is a stand-alone package for investigating phylogenetic trees plus associated metadata and is also integrated into EnteroBase to facilitate cutting edge navigation of genomic relationships among bacterial pathogens.

Legacy MLST (multilocus sequence typing) based on seven housekeeping genes was introduced 20 years ago (Maiden et al. 1998) and is now routinely used for the characterization of numerous bacterial pathogens (Jolley and Maiden 2014). MLST assigns distinct integer numbers to each unique sequence (allele) and a distinct integer number, the sequence type (ST), to each unique combination of allelic integers. Unrelated STs share few alleles or none at all. In contrast, STs that share all but one or two alleles are considered to be strongly related even if the differing alleles contain multiple SNPs due to recombination. The largest legacy MLST databases contain data on ≥60,000 bacterial strains (https://pubmlst.org/databases.shtml).

In order to support epidemiological tracking of transmission networks and disease control, the resolution achieved by MLST was recently expanded to encompass more than seven gene fragments. Expanded MLST schemes can include all 53 genes encoding ribosomal proteins (rMLST) (Jolley et al. 2012), thousands of core genes that are present in most isolates of a species or genus (core genome MLST, cgMLST) (Mellmann et al. 2011; Maiden et al. 2013; Moura et al. 2016), or even all the genes in the entire genome (whole genome MLST, wgMLST) (Nadon et al. 2017). We have recently developed EnteroBase (https://enterobase.warwick.ac.uk), a genotyping website for selected enteric pathogens (Alikhan et al. 2018). EnteroBase automatically assembles Illumina short reads into contigs and assigns the assembled sequences to MLST alleles and STs at all levels of resolution from legacy MLST through to wgMLST. EnteroBase performs these operations for short reads that are in the public domain or uploaded by users.

In April 2018, EnteroBase contained ∼130,000 *Salmonella* genomes and >65,000 *Escherichia* genomes, and the numbers of sets of Illumina short reads in the public domain continues to grow rapidly (Alikhan et al. 2018). A driving force behind developing such large databases is to facilitate our understanding of epidemiological and population genetic phenomena among isolates from distinct geographical sources and over extended time scales. Initially, the genetic relationships of legacy STs were represented by phylograms based on hierarchical clustering methods, an approach which can be very useful for visualizing deeper branching structures. Phylograms may, however, be problematic for the presentation of large numbers of genotypes because each genotype is represented by a unique branch, even when multiple genotypes are identical. An example of this problem arises when visualizing the allelic distances between 99,722 *Salmonella* spp. strains from 3902 legacy MLST STs. The associations between serovars and genetic clades are somewhat difficult to interpret within the default presentation of this phylogram by iTOL (Fig. 1A; Letunic and Bork 2016). Dendrograms generated by other programs (FigTree [v.1.4.3, http://tree.bio.ed.ac.uk/software/figtree/]; Dendroscope [Huson and Scornavacca 2012]) from large data sets were also difficult to interpret. Still other graphical user interfaces were unable to even depict this large number of items, including PHYLOViZ 2.0 (Nascimento et al. 2017), SplitsTree4 (Huson and Bryant 2006), EvolView (He et al. 2016), Microreact (Argimon et al. 2016), TreeDyn (Chevenet et al. 2006), TreeView (Page 1996), and Phandango (Hadfield et al. 2018). Similarly, handling more than 5000 genomes presents problems for de novo sequence-based SNP comparisons (Mazariegos-Canellas et al. 2017), and trees based on phylogenetic algorithms are difficult to comprehend when they contain large numbers of nodes (Fig. 1B,D).

**Figure 1.**
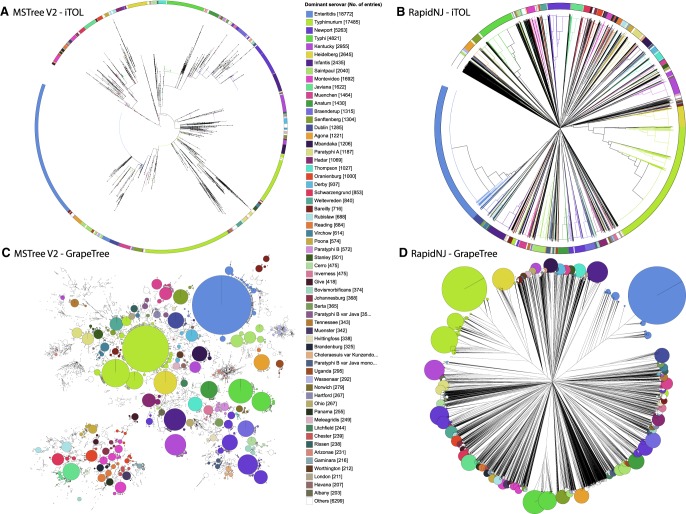
Visualization of 3902 legacy *Salmonella* MLST STs from 99,722 genomic assemblies in EnteroBase (Alikhan et al. 2018) by a phylogram versus a minimum spanning tree. (*A*,*B*) iTol (Letunic and Bork 2016) visualization of genetic relationships. Nodes at the ends of the terminal edges represent each of the 99,722 genomic assemblies. (*C*,*D*) GrapeTree visualization of genetic clusters. Nodes represent each of the 3902 STs, with diameters scaled to the number of assemblies. In *C*, edges between nodes mark allelic distances of 1–2 of the seven loci. (*A*,*C*) Representation of a minimum spanning tree generated by MSTree V2 in Newick format. (*B*,*D*) Representation of a neighbor-joining tree generated by RapidNJ (Simonsen et al. 2011) in Newick format. Color codes for the 60 most common serovars are indicated in the central key legend and used to color branches plus wedges in an external circle (*A*,*B*) or individual nodes (*C*,*D*). Interactive versions of the trees can be found at (*A*) http://bit.ly/2qH06jp, (*B*) https://bit.ly/2mDOpbS, (*C*) http://bit.ly/2H69dkG, and (*D*) https://bit.ly/2LG62Tl.

An alternative to phylograms is minimum spanning trees, which have less demanding graphical requirements because they map clusters of related nodes in 2D space (Francisco et al. 2012; Nascimento et al. 2017). A commercial software program (BioNumerics, Applied Maths) introduced an improved visualization of a minimum spanning tree to microbiologists in the early 1990s, which reduced complexity by grouping isolates with identical STs within single nodes whose diameter reflected the numbers of isolates. A similar visualization was subsequently offered by the noncommercial PHYLOViZ software (Francisco et al. 2009). We have now extended these approaches with GrapeTree, a software package that supports the efficient visualization of minimum spanning trees and phylograms from character data. An initial indication of its capabilities can be gained by comparing the representations of genetic relationships according to legacy MLST data by iTOL (Fig. 1A,B) and GrapeTree (Fig. 1C,D).

Calculating minimum spanning trees from legacy MLST is quick and efficient because legacy MLST is based on only seven loci, and allelic calls for each of the seven loci are a prerequisite for calling an ST, i.e., no missing data. As a result, the GrapeTree visualization in Figure 1C took only 1.5 min. However, the cgMLST of *Salmonella* spans 3002 loci (Alikhan et al. 2018), and STs routinely include low levels of missing data because some cgMLST genes are occasionally deleted or are not identified due to various bioinformatics problems in the assembly of genomes from short reads. As a result, multiple sets of almost identical STs exist in EnteroBase that only differ due to missing data, but each of which is, nevertheless, a unique node in a phylogram because its allelic content differs from those of other STs. As demonstrated below, missing data are also a problem for the classical minimum spanning tree approach (henceforth MSTree) implemented by BioNumerics and goeBURST (Francisco et al. 2009). We have therefore implemented MSTree V2, which is an improved algorithm for generating minimum spanning trees from character sets that contain missing data.

Here we present GrapeTree, a web browser application that efficiently reconstructs and visualizes intricate minimum spanning trees together with detailed metadata.

## Results

### Overview of GrapeTree features

**Figure 2.**
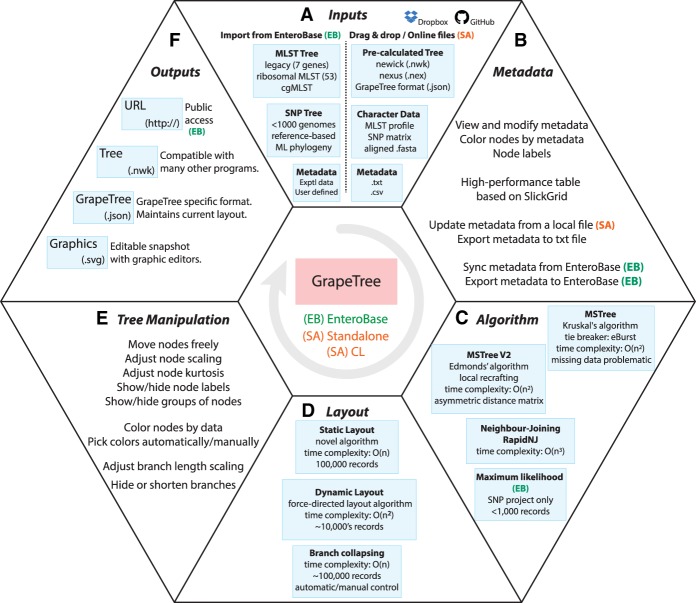
Overview of GrapeTree features. GrapeTree can run within the EnteroBase environment (EB; green), in stand-alone mode (SA; red), or in command line mode (CL), which disables graphic interactions. Options for CL mode are shown by typing “grapetree -h” after installation. All features are common to EB and SA, except where indicated in the figure. A demonstration version of GrapeTree is available for experimentation at https://achtman-lab.github.io/GrapeTree/. (*A*) Inputs for GrapeTree EB (*left*) and GrapeTree SA (*right*). (*B*) Metadata capabilities. (*C*) Algorithms used for tree constructions. (*D*) Static and dynamic layout and branch collapsing. (*E*) Tree manipulation. (*F*) Outputs.

GrapeTree is a fully interactive, tree visualization program that supports facile manipulations of both tree layout and metadata. The visual component of GrapeTree is implemented in HTML/JavaScript and served through a web server based on the Flask web framework (Python 2.7). GrapeTree is available as a stand-alone version (GrapeTree SA), which calculates trees from character data, visualizes precalculated trees, and annotates them with information from metadata (Fig. 2). Calculating trees is handled by an independent module (CL), which calls Python NumPy as well as external C++ programs for efficiency. CL can also be run in command line mode, in which case it terminates after generating the desired tree in Newick format. GrapeTree has also been integrated into larger web services through wrapper functions. The wrappers provide bidirectional communication with database servers containing information from hundreds of thousands of bacterial genomes and their associated metadata (Supplemental Fig. S1). The version of GrapeTree provided by EnteroBase (GrapeTree EB) only displays trees calculated from EnteroBase data because the module for performing those tree calculations is fully integrated into EnteroBase. Jolley has written a separate GrapeTree wrapper specific for the BigsDB website/database environment (Jolley and Maiden 2010) and thereby enabled GrapeTree functionality for all the databases served by PubMLST.

#### Inputs into GrapeTree SA

**Figure 3.**
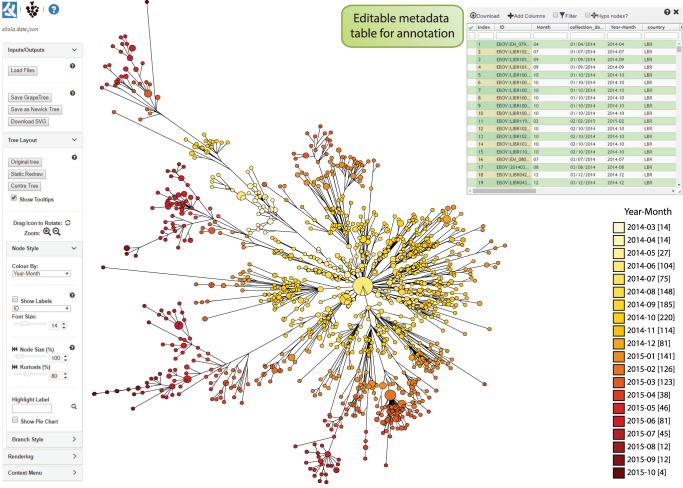
GrapeTree (SA) interface exemplified with a precalculated Newick tree based on 1610 Ebola genomes from the West African epidemic of 2013–2016. The tree and metadata were retrieved from microreact.org (https://microreact.org/project/west-african-ebola-epidemic), including a column designated “collection_date.” A new data column (year-month, *upper right*) was added to the metadata panel that contained the year and month information from “collection_date,” and this column was used to color-code the visualization as a temporal gradient (key, *lower right*). Branches spanning <0.22 substitutions per site were collapsed for clarity. The data indicate progressive radiation from a central source, consistent with published findings (http://www.nextstrain.org/ebola) (Dudas et al. 2017). An interactive version of this figure and metadata can be found at http://bit.ly/2EUkEKp.

GrapeTree SA accepts matrices of character data (MLST allelic profiles or SNPs), aligned multiple-FASTA files, precalculated tree files in standard formats (Newick or NEXUS), and comma or tab-delimited text for metadata (Fig. 2). Such files can be uploaded into GrapeTree SA by dragging and dropping from a user's local workstation, pasting the content into an input box, or from online sources. The GrapeTree SA backend module calculates trees from character data or FASTA files, whereas precalculated tree files are rendered without further modification. To illustrate this flexibility, Figure 3 shows a GrapeTree representation of a phylogenetic tree of 1610 Ebola genomes from the 2013–2016 Ebola epidemic in West Africa (Dudas et al. 2017) which was downloaded together with associated metadata from Microreact (Argimon et al. 2016). Note that this is a topologically correct visualization of a real phylogenetic tree, including internal hypothetical nodes, some of which have been collapsed for clarity.

#### Metadata

GrapeTree implements a high performance spreadsheet based on JavaScript SlickGrid (https://github.com/6pac/SlickGrid) that allows users to view and modify metadata that are associated with the individual entries (Fig. 3, top right). Additional columns from other experimental data or user-defined fields can be imported from EnteroBase into the metadata table in GrapeTree EB. For GrapeTree SA, the metadata can be exported locally, and novel metadata columns can be added to the exported data using a text editor or Microsoft Excel and re-imported. Any column can be used to color and/or label tree nodes. For example, an attractive presentation of a temporal gradient was implemented by reformatting downloaded metadata in Figure 3. The color codes for metadata are assigned automatically but can be changed manually, and the user can specify the number of colors (right click on key legend). In the metadata panel, metadata columns can be sorted and/or filtered at will to select individual entries.

#### Selecting genotypes

Entries that are selected in the metadata panel are immediately highlighted by red circles in the tree. Tree nodes can also be selected by pressing the shift key while opening a selection box over those nodes with a mouse. The ability to select a subset of the displayed nodes facilitates focused attention on individual groups of related genotypes. For example, we re-investigated the global relationships of recently described isolates of *Salmonella* serovar Typhimurium of legacy MLST ST313 and ST302 from Africa and the UK (yellow polygon in Supplemental Fig. S1A; Ashton et al. 2017). Legacy MLST STs were used as metadata to identify and select these genomes among 19,670 Typhimurium genomes in a GrapeTree based on cgMLST STs. The selected genomes were displayed within a new EnteroBase workspace (click EnteroBase\Load Selected), and used to generate a Neighbor Joining (NJ) tree in a second GrapeTree EB window (Supplemental Fig. S1B).

#### Algorithms

GrapeTree implements Kruskal's algorithm (Kruskal 1956) for a classical MSTree, Edmonds’ algorithm (Edmonds 1967) for MSTree V2, as well as the FastME V2 (Lefort et al. 2015) and RapidNJ (Simonsen et al. 2011) implementations of Neighbour-Joining. Maximum likelihood core SNP matrices can be calculated against a selected reference genome within EnteroBase using RAxML (Stamatakis 2014) for SNP projects containing up to 1000 genomes and then visualized by GrapeTree EB.

MSTree V2 is a novel minimum spanning tree which is better suited for handling missing data than are classical MSTrees. The workflow involved in calculating MSTree V2 is summarized in Supplemental Figure S2. First, a directed minimal spanning arborescence (dMST) (Edmonds 1967) is calculated from asymmetric (directional) distances with tie-breaking of coequal branches based on allelic distances from a harmonic mean. Local branch recrafting is subsequently performed to eliminate the spurious branches that can arise within minimum spanning trees. Further details are provided in Methods.

#### Layout and tree manipulation

Complex trees are difficult to visualize with clarity. In order to address this issue, GrapeTree initially collapses branches if there are more than 20,000 nodes and then uses a static layout that splits the tree layout task into a series of sequential node layout tasks in an attempt to prevent overlapping child nodes (Supplemental Fig. S3). Our implementation (Supplemental Material) provides a solution to this task in linear time complexity. The resulting layout can be further adjusted by a dynamic layout on the entire tree or on selected subtrees, using the force-directed algorithm (Dwyer 2009) in the JS D3 library (Supplemental Material). Users can also manually enforce a preferred layout by rotating selected nodes and branches.

Many other visual aspects of a GrapeTree can also be customized by the user (Fig. 2). In particular, complex trees with numerous nodes can be simplified by manually collapsing branches connecting subsets of related nodes or by setting a global threshold of differences below which all related branches are collapsed (Supplemental Material). The relationship between node size and numbers of entries can be adjusted in absolute terms or by adjusting the kurtosis (Supplemental Material).

Trees can be manipulated manually with a mouse by dragging branches, clicking on buttons, entering numbers into text boxes, or choosing settings through sliders. Additional options appear after right clicking. The metadata columns from the metadata table that are used for the presentation of text labels or node colors can be freely chosen from drop-down lists. Right clicking on the key table allows changes in presentation, including color codes. Options under branch length allow branches with lengths above a given threshold to be cropped or hidden, as in Figure 3. It is also possible to toggle the display of branch lengths and/or node labels.

#### Outputs

GrapeTree can export the current state of the browser window as a JSON file for use in future GrapeTree sessions. The JSON file includes both the tree layout and all metadata and facilitates sharing of GrapeTree sessions between collaborators or with the general public. The screen figure can be independently exported for manipulation with other software in Scalable Vector Graphics (SVG) format, and the underlying phylogenetic tree can be exported in Newick tree format. GrapeTree supports saving local metadata in tab-delimited text format. GrapeTree EB can also upload modified trees and metadata to EnteroBase and provide URLs for their public access via EnteroBase.

### Algorithms and performance

#### Comparative analyses with simulated data

We compared the accuracy of MSTree V2 against that of a classical MSTree as implemented by goeBURST (Francisco et al. 2009) on the basis of Kruskal's algorithm. We also compared these results with the intermediate MSTree (dMST) calculated with Edmonds’ algorithm within GrapeTree prior to local branch recrafting. These results were compared with the accuracy of NJ trees as a representative of phylogenetic approaches. All algorithms were tested on pairwise distance matrices calculated from simulated MLST data from 2000 loci of known evolutionary history and spanning a wide variety of genetic diversity (Methods\Data simulations).

**Figure 4.**
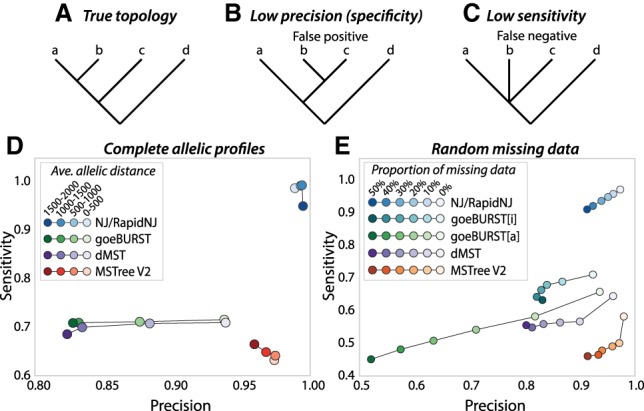
Precision and sensitivity of trees calculated by different algorithms from simulated allelic data. Trees were calculated for 100 replicates from each of 24 simulated phylogenies that differed in substitution rates (0.00001–0.07). (*A*–*C*) Cartoon trees demonstrating the true topology (*A*), low precision due to false positives (*B*), and low sensitivity due to false negatives (*C*). (*D*) Average sensitivity vs. precision in the absence of missing data after quartet analysis of branches calculated by NJ, goeBURST, the dMST intermediate stage prior to local branch recrafting in MSTree V2, and the full MSTree V2 algorithm including local branch recrafting. Values calculated by the quartet analyses were assigned to four bins according to allelic distances as indicated in the key. (*E*) Average sensitivity vs. precision after quartet analysis of branches calculated with different levels of random missing data for substitution rate 0.00005. goeBURST was forced to treat missing values as additional alleles by encoding them as 0 (goeBURST[a]) or to ignore them by encoding them as “–” (goeBURST[i]; defaults in MSTree). Values calculated by the quartet analyses were assigned to six bins according to the proportion of missing data as indicated in the key.

The results were tested for precision/specificity (frequency of true positives) as well as sensitivity (inverse frequency of false negatives) by comparing the calculated topologies against the known history of evolutionary changes in the simulated data (Fig. 4A). Calculated topologies with different branching order were scored as false positives (Fig. 4B). Similarly, quartets in which more than two branches descended from a single node (polytomies) (Fig. 4C) were scored as false negatives because only binary branch splits had been allowed within the simulations.

MSTree V2 was associated with very high precision (>0.95), almost as high as that manifested by NJ (Fig. 4D). Somewhat lower levels of precision were measured for goeBURST and dMST, ranging from 0.93 at low allelic distances down to 0.83 at greater allelic distances. Sensitivity was also very high for NJ (almost 1.0), but much lower for either MSTree V2 (∼0.65) or the classical MSTree algorithms (∼0.7).

We also compared precision and sensitivity with increasing proportions of missing data by modifying the input distance matrix calculated by goeBURST. To this end, missing alleles were replaced with 0, which forces missing values to be treated as an additional allele (designated goeBURST[a]) or encoded as “–”, which excludes the comparison of that locus from pairwise distances between profiles (designated goeBURST[i]). The results showed that MSTree V2 and NJ maintained high precision even up to 50% missing data (Fig. 4E). The precision of goeBURST and dMST was lower at all levels of missing data, ranging down to 0.5 precision at 50% missing data for goeBURST[a]. Sensitivity was slightly reduced by missing data for all algorithms, including NJ, and once again, the lowest levels of sensitivity were observed for MSTree V2.

#### Comparison of NJ, MSTree, and MSTree V2 on real data

**Figure 5.**
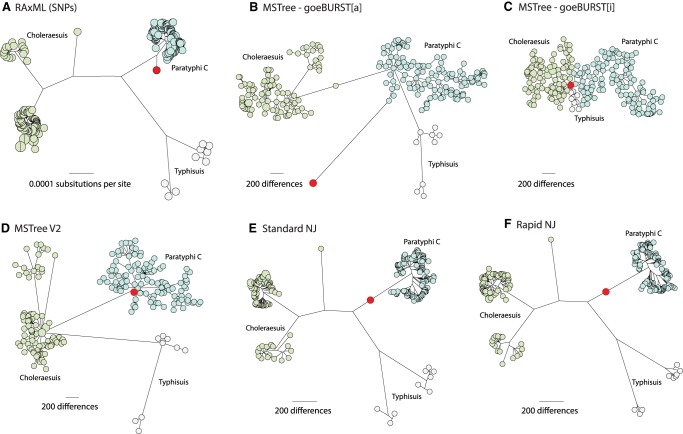
Comparisons of different topologies produced by six algorithms when extensive missing data is present. Trees were calculated from 20,114 nonrecombinant, core genomic SNPs (*A*) or 3002 loci in the cgMLST V2 scheme (Alikhan et al. 2018) (*B*–*F*) that were found in 218 modern genomes from *Salmonella* serovars Paratyphi C, Typhisuis, or Choleraesuis (Zhou et al. 2018). The modern genomes were supplemented by one ancient genome (Ragna; red) that had been reconstructed from an 800-yr-old skeleton. The algorithms used were maximum likelihood (*A*, RAxML), MSTree (*B*, GoeBURST[a]; *C*, GoeBURST[i]), MSTree V2 (*D*, GrapeTree), NJ (*E*, FastMe), or RapidNJ (*F*, RapidNJ), and all trees were visualized in GrapeTree SA. Nodes are color-coded by serovar. Due to fragmentation in the ancient Ragna DNA and intermediate levels of genome coverage, cgMLST alleles in Ragna could only be called for 215 (<10%) of the 3002 cgMLST loci, and the remainder of the cgMLST alleles were scored as missing data. Similarly, only 19,245 (96%) of the SNPs could be called in the Ragna genome. The Ragna genome is on a side-branch that diverged prior to the coalescence of the crown branch leading to modern Paratyphi C and differs from that coalescent by 263 SNPs. The correct position and branch length of the Ragna branches are as shown in *A*. Ragna is on an artificial, long terminal branch in *B* because all missing data count as different alleles. Ragna is central in part *C* because it is ≤215 cgMLST allele differences from all modern genomes and therefore forms an artificial central hub for all the genomes. MSTree V2 (*D*) maps Ragna to a tiny side branch preceding the Paratyphi C coalescent, similar in topology to how it was mapped on the basis of SNPs (*A*). However, NJ (*E*) and RapidNJ (*F*) mapped Ragna incorrectly near the base of the long, main branch leading to the crown group of Paratyphi C. The mapping of Ragna to the main branch rather than on its own side branch resulted because those algorithms calculated a negative distance from Ragna to the main branch. Interactive versions of each tree are available at (*A*) http://bit.ly/2vuFIIb, (*B*) http://bit.ly/2HF5tYt, (*C*) http://bit.ly/2qDD3GT, (*D*) http://bit.ly/2JRBvkQ, (*E*) https://bit.ly/2B6IS7v, and (*F*) https://bit.ly/2z2LWRb.

We also examined the behavior of these algorithms with a relatively uniform group of 222 genomes from related serovars within the *S. enterica* Para C Lineage, including one ancient Paratyphi C genome which contained large amounts of missing data (Zhou et al. 2018). A maximum-likelihood phylogenetic tree of nonrecombinant SNP data (Fig. 5A) placed the 800-yr-old ancient DNA (red node) on an early side branch predating the most recent common ancestor of modern members of serovar Paratyphi C. However, cgMLST data yielded classical MSTrees of different topologies, possibly due to the extent of missing data in the ancient genome. goeBURST[a] assigned the ancient genome to a spurious long branch extending sideways from Paratyphi C (Fig. 5B), while goeBURST[i] collapsed all branch distances, making it difficult to distinguish the individual serovars, and assigned the ancient genome to the center of the entire tree (Fig. 5C). Our general experience is that the classical minimum spanning tree algorithm generally draws faulty topologies when confronted with missing data and usually erroneously places nodes with extensive missing data in central positions.

Although there were subtle differences in branch lengths and detailed topologies between the MSTree V2 tree of cgMLST data (Fig. 5D) and the SNP tree (Fig. 5A), the general clustering into discrete groups was more or less concordant, as was the position of the ancient genome. Phylogenies of cgMLST alleles with NJ (Fig. 5E) or RapidNJ (Fig. 5F) yielded similar topologies for modern genomes to that of the SNP tree but incorrectly placed the aDNA Ragna genome near the base of the branch leading to Paratyphi C, rather than on a more recent side-branch.

## Discussion

Our analyses of simulated data showed higher precision with MSTree V2 than with dMST, which demonstrates the importance of local branch recrafting in MSTree V2 for the accuracy of calls (Fig. 4). Precision was also slightly higher for dMST than for either of the goeBURST algorithms at intermediate levels of missing data, which demonstrates that the directed MST approach adopted in MSTree V2 contributes to improved accuracy. The trade-off is that this high precision is accompanied by a slightly lower sensitivity than is true of classical minimum spanning trees.

### Balanced versus unbalanced quartets

Classical minimal spanning trees and MSTree V2 yielded considerably lower sensitivity (more false negatives) with the simulated data than did NJ. We suspected that this observation might reflect the use of inferred hypothetical ancestral nodes for node clustering by NJ because minimum spanning trees simply join nearest neighbors without calculating a possibly shorter path via hypothetical ancestral nodes. In that case, the low sensitivity of minimum spanning trees might be restricted to particular branching patterns rather than representing a general phenomenon. To test this hypothesis, we compared the accuracy of minimum spanning tree algorithms (and of NJ) between the balanced and unbalanced quartets within the simulated data (Supplemental Fig. S4). All algorithms yielded very high precision and sensitivity with balanced quartets (Supplemental Fig. S4C). NJ also yielded high precision and sensitivity for unbalanced quartets, unlike goeBURST or dMST, where sensitivity was always low and precision decreased with greater allelic diversity. The sensitivity with unbalanced quartets was even (slightly) lower with MSTree V2, but in this case, precision remained quite high (Supplemental Fig. S4D).

We attribute the observed low sensitivity of minimum spanning trees with unbalanced quartets to ambiguities in joining node 4 to the group of nodes 1, 2, and 3 when node 4 is equidistant from all three other nodes (Supplemental Fig. S4A,B). A classical MSTree attempts to connect node 4 to the founder node, which is node 1 or 2 in an unbalanced quartet, due to its reliance on the eBURST heuristic for choosing between equidistant pairs of nodes. At low levels of genetic divergence, most allelic differences reflect single nucleotide changes, and the behavior of a classical MSTree is likely to be correct (higher precision). At higher sequence divergence, the eBURST heuristic is no longer as appropriate, because allelic differences may well result from multiple mutations. Multiple mutations result in lessened consistency between allelic distances and the numbers of mutational events and correspondingly lower precision.

In contrast, MSTree V2 does not use the eBURST heuristic but instead breaks branches representing unbalanced splits during the branch recrafting stage and rejoins them to the centroid nodes in the subtrees (Supplemental Fig. S5), which removes most inaccurate topologies and improves precision. The slightly lower sensitivity of MSTree V2 in comparison to classical MSTree algorithms (Fig. 4) likely reflects the fact that, while MSTree V2 removes erroneous topologies, it simply makes no attempt whatsoever to resolve topologies within unbalanced quartets.

#### Speed and memory requirements

Our observations show that phylogenetic topologies and branch lengths are more accurately depicted by NJ trees or by other true phylogenetic methods than by MSTrees. For GrapeTree users, we would recommend using the maximum likelihood algorithm on SNPs when possible. However, EnteroBase limits such analyses to a maximum of 1000 closely related genomes in order not to hamper its performance for multiple users. We therefore recommend using GrapeTree SA for larger SNP projects. We note, however, that handling SNP distance matrices from more than 5000 genomes remains problematical (Mazariegos-Canellas et al. 2017).

**Figure 6.**
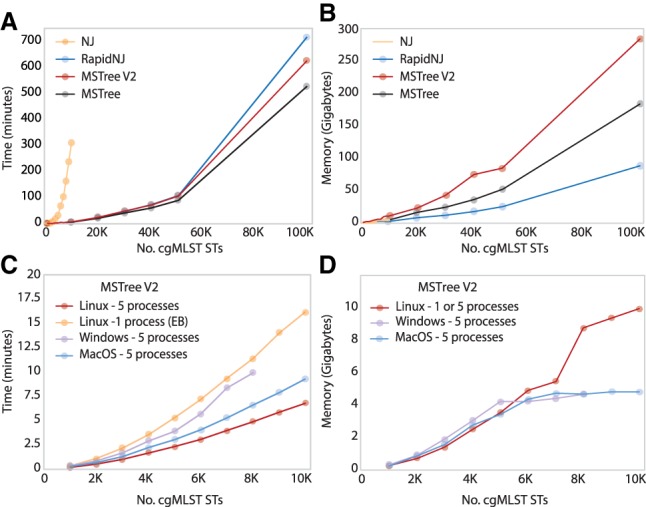
Time and memory required for different algorithms to calculate genetic relationships from cgMLST STs of *Salmonella*. Each point represents the average time and memory (three replicates) for GrapeTree in command line mode to calculate a tree from an independent random subset of 96,108 cgMLST STs from the EnteroBase *Salmonella* database. Exceptionally, the *rightmost* points in *C* and *D* represent only single replicates, and only samples of ≤10,000 cgMLST STs were tested with NJ (FastMeV2 implementation). (*A*,*B*) Time and memory profiles using five processes within a Linux machine. (*C*,*D*) Time and memory profiles of the MSTree V2 algorithm using various OS platforms. The Windows workstation was unable to complete calculations with >8000 cgMLST STs, possibly due to insufficient RAM. The Windows and MacOS workstations each contained four cores and 8 GB of RAM, whereas the Linux workstation contained 40 cores running at 2 GHz and 1 TB of RAM.

GrapeTree can handle large data sets. Its implementations of MSTree and MSTree V2 have a time complexity of O(n^2^), and GrapeTree stores the calculated pairwise genetic differences in a highly efficient data structure (Python NumPy). We quantified the time and memory requirements of multiple algorithms by using GrapeTree SA in command line mode to calculate trees based on increasing numbers of *Salmonella* cgMLST STs, each of which includes 3002 integer values (Fig. 6). An NJ cgMLST tree from 10,000 genomes took over 4 h to calculate (Fig. 6A). In contrast, calculating a distance matrix for up to 10,000 STs required only a few minutes and <10 GB of RAM with the MSTree, MSTree V2, or RapidNJ algorithms (Fig. 6A,B). Laptops running under MacOS or Windows 10 could readily handle 8000 STs (Fig. 6C,D). For larger data sets, we would recommend using multiple parallel processes on a Linux server. With five processes, our server could handle 100,000 STs in less than 700 min and used a maximum of ∼300 GB of RAM (Fig. 6A,B). We would recommend using MLST V2 over RapidNJ for interacting with large data sets because it allows ready visualization of many details which are obscured in phylograms containing large numbers of nodes (Fig. 1). MSTree V2 within GrapeTree EB also provides the ability to rapidly drill down from very large data sets containing missing data to clusters of scientific interest (Supplemental Fig. S1), which is not readily possibly with other approaches.

### Conclusions

Core genome MLST provides a feasible approach for providing public access to hundreds of thousands of bacterial genotypes at the genomic level (Alikhan et al. 2018). Access to such databases will facilitate international collaboration and support the global surveillance of bacterial pathogens. A major current bottleneck has been the lack of tools that can handle such data sets for the elucidation of genetic relationships and the visualization of clusters of related genotypes plus their metadata.

GrapeTree now allows users to explore the fine-grained population structure and phenotypic properties of large numbers of genomes in a web browser. GrapeTree SA is a stand-alone program that provides bioinformaticians with a tool for rapidly investigating the relationships of genomes of interest by NJ or minimal spanning trees of SNPs or MLST data. Customized versions of GrapeTree provide the same graphical front-end functionality to EnteroBase (Alikhan et al. 2018) and BIGSdb, thereby providing access to cgMLST schemes from most of the major bacterial pathogens. GrapeTree supports the input of data from a variety of sources and export to a variety of formats, thus empowering the public exploitation and sharing of genomic data by nonbioinformaticians.

## Methods

### Detailed explanation of novel aspects of MSTree V2

#### Calculation of asymmetric distances

In order to handle missing data correctly, MSTree V2 implements a directional measure based on normalized, asymmetric Hamming-like distances, *d*(*u* → *v*), between pairs of STs. This approach assumes that one of the pair of STs is the ancestor of the other and treats missing data as deletions from the ancestor to the descendant.

Given a set of STs *S* and a profile *π*(*s*) for each ST with a set of loci *L*, we define *d*(*u* → *v*) between an ordered pair of two STs (*u, v*) ∈ *S* as

$$d(u\to v)=\underset{l\;\in \;L}{\sum }\frac{1_{\left\{(\pi_{l}(u)\neq \pi_{l}(v))\wedge (\pi_{l}(v)\neq 0)\right\}}}{N_{v}},$$

with $N_{v}=\sum_{l\in L}1_{\left\{\pi_{l}(v)\neq 0\right\}}$ and assuming 0 to be a missing value in all *π*. All possible values of these distances for each locus in the calculation of *d*(*u* → *v*) are illustrated in Supplemental Figure S2B. Note that these distances do not form a metric, because *d*(*u* → *v*) ≠ *d*(*v* → *u*) when missing values are present. We can then define a fully connected graph *G*(*V, E*) with *V* = *S* and directed edges (*u* → *v*) ∈ *E* weighted by their distance. By analogy to a minimum spanning tree for undirected graphs, we compute a direct minimum spanning tree (dMST, also designated minimal spanning arborescence) on *G* in polynomial time with Tarjan's rapid implementation (Tarjan 1977) of Edmonds’ algorithm (Edmonds 1967), using the Edmonds-alg package (http://edmonds-alg.sourceforge.net/).

#### Harmonic tie-breaking

During the construction of an MSTree, Bionumerics or goeBURST chooses between multiple co-optimal branches by tie-breaking according to the principles of eBURST (Feil et al. 2004) as summarized and extended by Francisco et al. (2009). The eBURST approach presumes that a clonal complex (lineage) is founded by a founder genotype and that genetic variants of that founder reflect the progressive accumulation of additional variations over time. A further implicit belief is that the number of variants decreases with distance from the founder genotype, such that the founder is equated with the central genotype with the greatest number of single locus variants, and edges between nodes are ordered based on their allelic distances. In case of a tie for directionality of connections, the founder status is assigned to the node with the greater number of single locus variants, double locus variants, triple locus variants, and/or number of strains assigned to that ST.

At cgMLST levels of resolution, the founder genotype may not be present in a comparison, which renders the eBURST model inappropriate for tie-breaking. Instead of depending on the preconceived properties of a theoretical founder genotype, MSTree V2 simply chooses central nodes between multiple co-optimal branches on the basis of the harmonic mean of allelic distances.

We define a *centroid* genotype, which is the genotype for any given population that has the smallest average allelic distance to all other genotypes in the same population. The harmonic mean of the allelic distances is used rather than an arithmetic mean in order to give higher weights to variants with smaller allelic distances to other STs. In a fully connected graph *G*(*V*,*E*) as defined above, we define the harmonic mean *ht*(*u*) of allelic distances for any node *u* ∈ *V* to other nodes as

$$ht(u)={\left(\frac{\sum_{\begin{array}{c} v\;\in V,\\ u\neq v \end{array}}d{\left(u\to v\right)}^{-1}}{|V|-1}\right)}^{-1}.$$

All directed edges *d*(*u* → *v*) are ordered in ascending order according to *ht*(*u*), with the frequency of occurrence of *u* as the final tie-break. This ordering results in a unique and optimal dMST with Edmonds’ algorithm. Furthermore, since we have a fully connected graph and *d* satisfies the triangular inequality, the length of the shortest (geodesic) path between any two vertices *u* and *v* is given by *d(u* → *v)*.

We note that *ht*(*u*)^−1^ is also known as ‘closeness centrality’ in network science (Newman 2010). Closeness centrality is usually defined for unweighted graphs as the inverse of the mean distance between vertices. However, some interesting properties arise when it is defined in our sense as the inverse of the harmonic mean distance between vertices: *ht*(*u*)^−1^ gives more weight to vertices that are close to the vertex of interest than to those far away, and it can also naturally deal with disconnected components.

#### Local branch recrafting

Edmonds’ algorithm attempts to minimize the sum of the edge lengths in the tree. However, the resulting dMST does not necessarily represent true phylogenetic relationships between strains because allelic distances do not always correlate with divergence time. We therefore implemented a subsequent branch optimization step that accounts for these discrepancies. Algorithm 1 gives an overview over the local branch recrafting (see also Supplemental Fig. S5), starting from the already computed dMST(*V*,*E*), where *E* is a distance matrix sorted in ascending order of allelic distances, and a forest *F* where each *u* ∈ *V* is a single tree *t*(*u*) ∈ *F*.

**Table:**
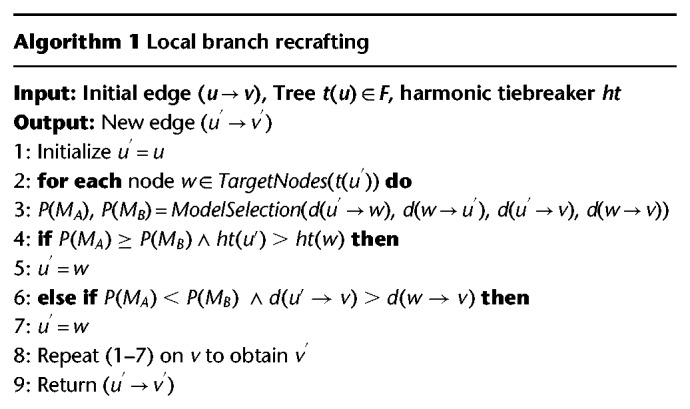


Optimizations are applied to both ends of each edge in the dMST(*V,E*) iteratively as shown in Supplemental Figure S5D. The *TargetNodes*() function picks a subset of the nodes in tree *t*(*u*) which are the centroids and the nodes that are directly connected to *u* (Supplemental Fig. S5D). The *ModelSelection*() function compares the maximum likelihoods of two models *M*_*A*_ and *M*_*B*_ (Supplemental Fig. S5B,C). Here we describe only the model selection process for *u*. Given *d*(*u* → *w*), *d*(*w* → *u*), *d*(*u* → *v*) and *d*(*w* → *v*), when assuming *d*(*u* → *v*) ≥ *d*(*w* → *v*), the proportions of invariable sites in branches *l*_*A*_, *k*_*A*_, *l*_*B*_ and *k*_*B*_ satisfy:

$$\;\underset{0\leq l_{A}\leq 1,0\leq k_{A}\leq 1}{\text{argmax}}\log P(M_{A}|l_{A},k_{A})$$

$$=\underset{0\leq l_{A}\leq 1,0\leq k_{A}\leq 1}{\text{argmax}}\log P(u\to w|l_{A})P(u\to v|l_{A},k_{A})P(w\to v|l_{A},k_{A})$$

$$\;=\underset{0\leq l_{A}\leq 1,0\leq k_{A}\leq 1}{\text{argmax}}|L|d(u\to w)\log(1-l_{A}^{2})+|L|(1-d(u\to w))\log(l_{A}^{2})$$

$$\;+|L|d(u\to v)\log(1-l_{A}k_{A})+|L|(1-d(u\to v))\log(l_{A}k_{A})$$

$$\;\begin{array}{c} +|L|d(w\to v)\log(1-l_{A}k_{A})+|L|(1-d(w\to v))\log(l_{A}k_{A})\;\;\;(1) \end{array}$$

$$\;\underset{0\leq l_{B}\leq 1,0\leq k_{B}\leq 1}{\text{argmax}}\log P(M_{B}|l_{B},k_{B})$$

$$=\underset{0\leq l_{B}\leq 1,0\leq k_{B}\leq 1}{\text{argmax}}\log P(w\to u|l_{B})P(u\to v|l_{B},k_{B})P(w\to v|l_{B},k_{B})$$

$$\;=\underset{0\leq l_{B}\leq 1,0\leq k_{B}\leq 1}{\text{argmax}}|L|d(w\to u)\log(1-l_{B})+|L|(1-d(w\to u))\log(l_{B})$$

$$\;+|L|d(u\to v)\log(1-l_{B}k_{B})+|L|(1-d(u\to v))\log(l_{B}k_{B})$$

$$\;\begin{array}{c} +|L|d(w\to v)\log(1-k_{B})+|L|(1-d(w\to v))\log(k_{B}),\;\;\;(2) \end{array}\;$$

where *L* is a set of loci in an MLST profile. Note that the direction of the distances between *u* and *w* are different in the two equations. Model *A* assumes *u* as the centroid node and adopts *d*(*u* → *w*) in Equation 1, whereas model *B* treats *w* as the centroid and thus uses *d*(*w* → *u*). We further denote

$$x=1-\frac{\left(1-d(w\to u))(1-d(w\to v))+(1-d(u\to v)\right)}{2}.$$

Then, the parameters in Equations 1 and 2 are calculated as

$$l_{A}=\sqrt{1-d(u\to w)}$$

$$k_{A}=\frac{1-(1/2)(d(u\to v)+d(w\to v))}{l_{A}}$$

$$l_{B}=1+\frac{xd(w\to u)}{d(u\to v)-2x}\;$$

$$k_{B}=1+\frac{xd(w\to v)}{d(u\to v)-2x}.$$

These parameters can then be used to calculate *P*(*M*_*A*_) and *P*(*M*_*B*_) using Equations 1 and 2.

### Data simulations

In order to compare various algorithms with MLST data of known evolutionary history, SimBac (Brown et al. 2016) was used to simulate the coalescence of 40 genomes of size 2 Mb. One hundred replicate simulations were performed without homologous recombination and assuming a constant population size for each of 24 different substitution rates ranging from 0.00001 to 0.07. Simulated MLST data were then obtained by splitting each of the final 40 genomic sequences into 2000 loci of 900 bp separated by 100-bp intergenic regions. Each unique locus within the 40 genomes was assigned a unique allelic integer, and these integers were used to generate allelic profiles for the simulated genomes within each replicate. The genetic distances between the 40 allelic profiles were used to compute classical MSTrees using goeBURST (Francisco et al. 2009) and a directed MSTree using GrapeTree MSTree V2. NJ trees were calculated with FastMEV2 (Lefort et al. 2015) and RapidNJ (Simonsen et al. 2011). In order to establish the effects of local branch recrafting, we also extracted the intermediate result (dMST) from MSTree V2 at the state immediately prior to recrafting.

We also tested the effects of missing data by scoring random allelic values from simulated data at a substitution rate of 0.00005 as missing values. Ten replicates were performed, spanning the range from 0 to 40,000 missing values in steps of 8000 of the 80,000 allelic values (0%–50%). These simulated data contained an average allelic distance of 107 (CI 95%: 6–211). In order to score the randomly selected values as missing, they were replaced with 0 (goeBURST[a]), which forces missing values to be treated as an additional allele by goeBURST, or encoded as “–” (goeBURST[i]), which excludes the comparison of that locus from pairwise distances between profiles.

## Data access

Interactive versions of trees in Figure 1: (A) http://bit.ly/2qH06jp; (B) https://bit.ly/2mDOpbS; (C) http://bit.ly/2H69dkG; (D) https://bit.ly/2LG62Tl. An interactive version of Figure 3 can be found at http://bit.ly/2EUkEKp. Trees presented in Figure 5 are available separately: (A) http://bit.ly/2vuFIIb; (B) http://bit.ly/2HF5tYt; (C) http://bit.ly/2qDD3GT; (D) http://bit.ly/2JRBvkQ; (E) https://bit.ly/2B6IS7v; and (F) https://bit.ly/2z2LWRb. Trees presented in Supplemental Figure S1 can be found at http://bit.ly/2vjTn4I and http://bit.ly/2H8py8F. Figure 1, A and B can be reconstructed by uploading the trees and metadata files available in Supplemental Data S1 into iTOL (http://itol.embl.de/). Other interactive figures can be visualized in GrapeTree SA using the source files in Supplemental Data S2. Source code and precompiled binaries for GrapeTree are available as Supplemental Data S3 and also deposited online at https://github.com/achtman-lab/GrapeTree. Simulation and evaluation scripts are also available in Supplemental Data S3 and on GitHub in the folder “simulations.” Online documentation and a live demo are available at http://enterobase.readthedocs.io/en/latest/grapetree/grapetree-about.html. The documentation is also available as Supplemental Data S4.

## Supplementary Material

Supplemental Material

## References

[GR232397ZHOC1] Alikhan N-F, Zhou Z, Sergeant MJ, Achtman M. 2018 A genomic overview of the population structure of *Salmonella*. PLoS Genet 14: e1007261. 29621240 10.1371/journal.pgen.1007261 PMC5886390

[GR232397ZHOC2] Argimon S, Abudahab K, Goater RJ, Fedosejev A, Bhai J, Glasner C, Feil EJ, Holden MT, Yeats CA, Grundmann H, 2016 Microreact: visualizing and sharing data for genomic epidemiology and phylogeography. Microb Genom 2: e000093. 28348833 10.1099/mgen.0.000093 PMC5320705

[GR232397ZHOC3] Ashton PM, Owen SV, Kaindama L, Rowe WPM, Lane CR, Larkin L, Nair S, Jenkins C, de Pinna EM, Feasey NA, 2017 Public health surveillance in the UK revolutionises our understanding of the invasive *Salmonella typhimurium* epidemic in Africa. Genome Med 9: 92. 29084588 10.1186/s13073-017-0480-7 PMC5663059

[GR232397ZHOC4] Brown T, Didelot X, Wilson DJ, De MN. 2016 SimBac: simulation of whole bacterial genomes with homologous recombination. Microb Genom 2 10.1099/mgen.0.000044. PMC5049688 27713837

[GR232397ZHOC5] Chevenet F, Brun C, Banuls AL, Jacq B, Christen R. 2006 TreeDyn: towards dynamic graphics and annotations for analyses of trees. BMC Bioinformatics 7: 439. 17032440 10.1186/1471-2105-7-439 PMC1615880

[GR232397ZHOC6] Dudas G, Carvalho LM, Bedford T, Tatem AJ, Baele G, Faria NR, Park DJ, Ladner JT, Arias A, Asogun D, 2017 Virus genomes reveal factors that spread and sustained the Ebola epidemic. Nature 544: 309–315. 28405027 10.1038/nature22040 PMC5712493

[GR232397ZHOC7] Dwyer T. 2009 Scalable, versatile and simple constrained graph layout. Eurographics 28 10.1111/j.1467-8659.2009.01449.x.

[GR232397ZHOC8] Edmonds J. 1967 Optimum branchings. J Res Nat Bur Standards 71B: 233–240.

[GR232397ZHOC9] Feil EJ, Li BC, Aanensen DM, Hanage WP, Spratt BG. 2004 eBURST: Inferring patterns of evolutionary descent among clusters of related bacterial genotypes from Multilocus Sequence Typing data. J Bacteriol 186: 1518–1530. 14973027 10.1128/JB.186.5.1518-1530.2004 PMC344416

[GR232397ZHOC10] Francisco AP, Bugalho M, Ramirez M, Carrico JA. 2009 Global optimal eBURST analysis of multilocus typing data using a graphic matroid approach. BMC Bioinformatics 10: 152. 19450271 10.1186/1471-2105-10-152 PMC2705362

[GR232397ZHOC11] Francisco AP, Vaz C, Monteiro PT, Melo-Cristino J, Ramirez M, Carrico JA. 2012 PHYLOViZ: phylogenetic inference and data visualization for sequence based typing methods. BMC Bioinformatics 13: 87. 22568821 10.1186/1471-2105-13-87 PMC3403920

[GR232397ZHOC12] Hadfield J, Croucher NJ, Goater RJ, Abudahab K, Aanensen DM, Harris SR. 2018 Phandango: an interactive viewer for bacterial population genomics. Bioinformatics 34: 292–293. 10.1093/bioinformatics/btx610 PMC5860215 29028899

[GR232397ZHOC13] He Z, Zhang H, Gao S, Lercher MJ, Chen WH, Hu S. 2016 Evolview v2: an online visualization and management tool for customized and annotated phylogenetic trees. Nucleic Acids Res 44: W236–W241. 27131786 10.1093/nar/gkw370 PMC4987921

[GR232397ZHOC14] Huson DH, Bryant D. 2006 Application of phylogenetic networks in evolutionary studies. Mol Biol Evol 23: 254–267. 16221896 10.1093/molbev/msj030

[GR232397ZHOC15] Huson DH, Scornavacca C. 2012 Dendroscope 3: an interactive tool for rooted phylogenetic trees and networks. Syst Biol 61: 1061–1067. 22780991 10.1093/sysbio/sys062

[GR232397ZHOC16] Jolley KA, Maiden MC. 2010 BIGSdb: scalable analysis of bacterial genome variation at the population level. BMC Bioinformatics 11: 595. 21143983 10.1186/1471-2105-11-595 PMC3004885

[GR232397ZHOC17] Jolley KA, Maiden MC. 2014 Using multilocus sequence typing to study bacterial variation: prospects in the genomic era. Future Microbiol 9: 623–630. 24957089 10.2217/fmb.14.24

[GR232397ZHOC18] Jolley KA, Bliss CM, Bennett JS, Bratcher HB, Brehony C, Colles FM, Wimalarathna H, Harrison OB, Sheppard SK, Cody AJ, 2012 Ribosomal multilocus sequence typing: universal characterization of bacteria from domain to strain. Microbiology 158: 1005–1015. 22282518 10.1099/mic.0.055459-0 PMC3492749

[GR232397ZHOC19] Kruskal JB. 1956 On the shortest spanning subtree of a graph and the traveling salesman problem. Proc Am Math Soc 7: 48–50.

[GR232397ZHOC20] Lefort V, Desper R, Gascuel O. 2015 FastME 2.0: a comprehensive, accurate, and fast distance-based phylogeny inference program. Mol Biol Evol 32: 2798–2800. 26130081 10.1093/molbev/msv150 PMC4576710

[GR232397ZHOC21] Letunic I, Bork P. 2016 Interactive tree of life (iTOL) v3: an online tool for the display and annotation of phylogenetic and other trees. Nucleic Acids Res 44: W242–W245. 27095192 10.1093/nar/gkw290 PMC4987883

[GR232397ZHOC22] Maiden MCJ, Bygraves JA, Feil E, Morelli G, Russell JE, Urwin R, Zhang Q, Zhou J, Zurth K, Caugant DA, 1998 Multilocus sequence typing: a portable approach to the identification of clones within populations of pathogenic microorganisms. Proc Natl Acad Sci 95: 3140–3145. 9501229 10.1073/pnas.95.6.3140 PMC19708

[GR232397ZHOC23] Maiden MC, van Rensburg MJ, Bray JE, Earle SG, Ford SA, Jolley KA, McCarthy ND. 2013 MLST revisited: the gene-by-gene approach to bacterial genomics. Nat Rev Microbiol 11: 728–736. 23979428 10.1038/nrmicro3093 PMC3980634

[GR232397ZHOC24] Mazariegos-Canellas O, Do T, Peto T, Eyre DW, Underwood A, Crook D, Wyllie DH. 2017 BugMat and FindNeighbour: command line and server applications for investigating bacterial relatedness. BMC Bioinformatics 18: 477. 29132318 10.1186/s12859-017-1907-2 PMC5683244

[GR232397ZHOC25] Mellmann A, Harmsen D, Cummings CA, Zentz EB, Leopold SR, Rico A, Prior K, Szczepanowski R, Ji Y, Zhang W, 2011 Prospective genomic characterization of the German enterohemorrhagic *Escherichia coli* O104:H4 outbreak by rapid next generation sequencing technology. PLoS One 6: e22751. 21799941 10.1371/journal.pone.0022751 PMC3140518

[GR232397ZHOC26] Moura A, Criscuolo A, Pouseele H, Maury MM, Leclercq A, Tarr C, Bjorkman JT, Dallman T, Reimer A, Enouf V, 2016 Whole genome-based population biology and epidemiological surveillance of *Listeria monocytogenes*. Nat Microbiol 2: 16185. 27723724 10.1038/nmicrobiol.2016.185 PMC8903085

[GR232397ZHOC27] Nadon C, Van Walle I, Gerner-Smidt P, Campos J, Chinen I, Concepcion-Acevedo J, Gilpin B, Smith AM, Man KK, Perez E, 2017 PulseNet International: vision for the implementation of whole genome sequencing (WGS) for global food-borne disease surveillance. Euro Surveill 22: 30544. 28662764 10.2807/1560-7917.ES.2017.22.23.30544 PMC5479977

[GR232397ZHOC28] Nascimento M, Sousa A, Ramirez M, Francisco AP, Carrico JA, Vaz C. 2017 PHYLOViZ 2.0: providing scalable data integration and visualization for multiple phylogenetic inference methods. Bioinformatics 33: 128–129. 27605102 10.1093/bioinformatics/btw582

[GR232397ZHOC29] Newman MEJ. 2010 Networks: an introduction. Oxford University Press, Oxford, UK.

[GR232397ZHOC30] Page RD. 1996 TreeView: an application to display phylogenetic trees on personal computers. Comput Appl Biosci 12: 357–358. 8902363 10.1093/bioinformatics/12.4.357

[GR232397ZHOC31] Simonsen M, Mailund T, Pedersen CNS. 2011 Inference of large phylogenies using Neighbour-Joining. In Biomedical Engineering Systems and Technologies: 3rd International Joint Conference, BIOSTEC 2010 Communications in Computer and Information Science, Vol. 127, pp. 334–344. Springer Verlag, New York.

[GR232397ZHOC32] Stamatakis A. 2014 RAxML version 8: a tool for phylogenetic analysis and post-analysis of large phylogenies. Bioinformatics 30: 1312–1313. 24451623 10.1093/bioinformatics/btu033 PMC3998144

[GR232397ZHOC33] Tarjan RE. 1977 Finding optimum branchings. Networks 7: 25–35.

[GR232397ZHOC34] Zhou Z, Lundstrøm I, Tran-Dien A, Duchêne S, Alikhan N-F, Sergeant MJ, Langridge G, Fokatis AK, Nair S, Stenøien HK, 2018 Pan-genome analysis of ancient and modern *Salmonella enterica* demonstrates genomic stability of the invasive para C lineage for millennia. Curr Biol 28: 2420–2428.e10. 30033331 10.1016/j.cub.2018.05.058 PMC6089836

