# Supplementary material for: GrapeTree: visualization of core genomic relationships among 100,000 bacterial pathogens
Source: Genome Res. 2018 Sep;28(9):1395–404. doi: 10.1101/gr.232397.117 (PMC6120633; doi:10.1101/gr.232397.117)
Supplement: Supplemental Material [file supp_28_9_1395__index.html]

GrapeTree: visualization of core genomic relationships among 100,000 bacterial pathogens — Supplemental Material 

# GrapeTree: visualization of core genomic relationships among 100,000 bacterial pathogens

## Supplemental Material

- Supplemental\_material.pdf
- Supplemental\_data\_S1.zip
- Supplemental\_data\_S2.zip
- Supplemental\_data\_S3.zip
- Supplemental\_data\_S4.pdf
