# Supplementary material for: GrapeTree: visualization of core genomic relationships among 100,000 bacterial pathogens
Source: Genome Res. 2018 Sep;28(9):1395–404. doi: 10.1101/gr.232397.117 (PMC6120633; doi:10.1101/gr.232397.117)
Supplement: Supplemental Material [file supp_gr.232397.117_Supplemental_material.pdf]

Supplemental Material

GrapeTree: Visualization of core genomic relationships among 100,000 bacterial pathogens

Authors: Zhemin Zhou[1*], Nabil-Fareed Alikhan[1], Martin J. Sergeant[1], Nina Luhmann[1], Cátia Vaz[2,5], Alexandre P. Francisco[2,4], João André Carriço[3], Mark Achtman[1*]

Affiliations:

[1]Warwick Medical School, University of Warwick, Gibbet Hill Road, Coventry, CV4 7AL, United Kingdom

[2]Instituto de Engenharia de Sistemas e Computadores: Investigação e Desenvolvimento (INESC-ID), Lisboa, Portugal

[3]Universidade de Lisboa, Faculdade de Medicina, Instituto de Microbiologia and Instituto de Medicina Molecular, Lisboa, Portugal

[4]Instituto Superior Técnico, Universidade de Lisboa, Lisboa, Portugal

[5]ADEETC, Instituto Superior de Engenharia de Lisboa, Instituto Politécnico de Lisboa, Lisboa, Portugal

[*]Corresponding authors:
Z.Z.: zhemin.zhou@warwick.ac.uk
M.A.: m.achtman@warwick.ac.uk

Table of Contents

Details of GrapeTree Layout 3

Branch collapsing. 3

Static layout. 4

Dynamic layout. 8

Supplemental Figure S1. Zooming in on a subset of data in GrapeTree EB. 10

Supplemental Figure S2. MSTree V2 algorithm and asymmetric distances. 11

Supplemental Figure S3. Node layout algorithm in GrapeTree. 12

Supplemental Figure S4. Sensitivity *vs* precision for balanced and unbalanced quartets. ... 13

Supplemental Figure S5. Flowchart and decision tree for branch recrafting. 14

Reference List 15

Details of GrapeTree Layout

Branch collapsing. The branch collapsing algorithm in the GrapeTree visualization takes account of both automatic and manual collapsing of branches within a tree. It requires a tree (*T*), constructed of nodes (*V*) and edges (*E*). The algorithm iterates once through a tree, resulting in time complexity of *O*(*v*), where *v* is the number of nodes in the tree (Algorithm 2).

Algorithm 2 Branch collapsing

Input: Tree $T(V, E)$
  sets of descendants $D(v)$ for any node $v \in V$,
  sets of nodes C which are manually collapsed,
  sets of nodes U which are manually expanded,
  automatic collapsing length K,
  length of edge $L(v)$ that connects node v to its parent
Output: collapsed GrapeTree
for each node $v \in U$ **do**
  for each descendant $d \in D(v)$ **do**
    $s(d) = False$
for each node $v \in C$ **do**
  for each descendant $d \in D(v)$ **do**
    $s(d) = True$
for each node $v \in V$ **do**
  if $s(v) = True \vee (s(v) = False \wedge L(v) \leq K)$ **then**
    if parent of v is empty **then**
      Move label of v to its parent
    Move all children of v to its parent
    Move all strains in v to its parent
    Delete v
Redraw GrapeTree with remaining nodes

Node size. GrapeTree has a general method for merging multiple entries into a single node containing indistinguishable genotypes (same ST, missing data), or after branches

are collapsed. The radius of each node, *r,* is dependent on the number of entries, *m*, and the settings in the control panel for “Kurtosis” (*k)* according to

$$r = m^{k/2}$$

Collapsing branches manually will change the relative node size automatically but can be subsequently adjusted for aesthetic improvements through changing Kurtosis.

Static layout. The static layout implemented by GrapeTree is a hybrid between the Bubble Tree algorithm [S1], and its earlier predecessor, the Equal-Daylight algorithm [S2] (Table 1). The node size in GrapeTree is proportional to the number of indistinguishable bacterial strains, and edges are proportional to inter-node distances and cannot be bent in order to ensure that they accurately represent genetic distances.

Table 1: Summary of differences in rendering between the layouts of GrapeTree, Bubble Tree and the Equal-daylight algorithm.

	GrapeTree	Bubble Tree	Equal-Daylight
Node size	Proportional to number of strains	Ignored	Ignored
Edge lengths	Proportional to inter-node distance	Ignored	Proportional to inter-node distance
Bends in edges	No	Allowed once	No

The GrapeTree static layout algorithm aims to optimize two properties: 1) separating all the nodes from each other and 2) distributing all the nodes as evenly as possible for the resolution of the layout across the two-dimensional surface (plane).

A tree *T(V,E)* is a special directed acyclic graph with one root node *r*. There is a unique shortest directed path from *r* to any other node $v \in V$. If a node *u* lies on the path from the root to node *v*, then *u* is an *ancestral* node of *v* and *v* is a *descendant* node of *u*. We denote the set of all ancestors of *v* as $ancestral(v)$ and the set of all descendants of *v*

as $descendant(v)$ respectively. We also denote $children(v)$ as a subset of nodes in $descendant(v)$ directly incident to *v*.

For any node *v* in a GrapeTree visualization, we want to locate all its descendants within an enclosing disk centered on *v* (Supplemental Fig. S3). For each node $c_i \in children(v)$, we draw c_i and $descendant(c_i)$ within a proportion of this enclosing disk, denoted as the child's circular sector, $CCS(c_i)$. In order to provide enough space to place c_i and $descendant(c_i)$, this sector must have a minimal central angle of $a_{CCS}(c_i)$ and a minimal radius of $r_{CCS}(c_i)$. Then it is easy to see that the placement for all $c_i \in children(v)$, do not overlap with each other as long as their *CCS*s are not overlapping. In order to achieve maximum resolution, namely distribute all children of *v* evenly in the enclosing disk, we introduce a separating arc *s*, which is an area that separates any two neighboring *CCS*s. All $children(v)$ are separated more as *s* increases. The value of *s* is limited by the fact that all *CCS*s and the separating arcs between them need to be placed within the enclosing disk centered on *v*, as mentioned above.

We denote *DCS*(*v*) (descendants circular sector) as a minimal circular sector that consists of all $CCS(c_i)$ for node *v*. The central angle of *DCS*(*v*) needs to be smaller than 2π, in order to be placed in the enclosing disk around *v* without overlapping.

We can also show that *T* has a non-overlapping layout if every node $v \in V$ has $a_{DCS}(v) \leq 2\pi$. We already know that *v* does not overlap with any node $d \in descendant(v)$, as described above. Node *v* also does not overlap with any $a \in ancestral(v)$, because *v* is a descendant of *a* and located in an enclosing disk that is centered on *a*. Finally, node *v* does not overlap with any other node *v'* that is neither its ancestor nor its descendant as well, because i) we can always find a node *a* that is the nearest common ancestor of both *v* and *v'* and ii) *v* and *v'* do not overlap because

both nodes are descendants of *a* that are located in different, non-overlapping *CCS*s. The whole layout problem can then be formulated as:

$$\max_{s} \text{ given that } \forall n \in N{:}\, a_{DCS}(v) \leq 2\pi$$

for any node $v \in V$ and a known value for *s*, *DCS*(*v*) can be calculated based on all *DCS*(c_i) for $c_i \in children(v)$. The recursion is illustrated by an example in Supplemental Fig. S3 and can be separated into the following two steps.

1) Calculate *CCS*(c_i). *CCS*(c_i) is a minimal circular sector that has to be large enough to include both node c_i and all its descendants in *DCS*(c_i). The area of *CCS*(c_i) is determined by three factors: the length *l* of the edge from *v* to c_i, the radius *r* of node c_i and the area of *DCS*(c_i), which is determined by both the central angle $a_{DCS}(c_i)$ and the radius $r_{DCS}(c_i)$. We draw two possible circular sectors, where *CS1* is a sector that tries to cover node c_i itself:

$$a_{CS1} = 2 * arcsin\left(\frac{r\ s}{l + r}\right)$$

$$r_{CS1} = l + 2r$$

in which the central angle covers c_i if $s \geq 1$, otherwise some overlapping (normally at the tips of the trees) is allowed. And *CS*2 is a sector that guarantees to cover *DCS*(c_i):

$$a_{CS2} = 2\ max\left(arcsin(\frac{r_{DCS}(c_i)}{l + r}), poleMove([r_{DCS}(c_i), 1/2\ a_{DCS}(c_i)], [(l + r), \pi])\right)$$

$$r_{CS2} = l + r + r_{DCS}(c_i)$$

The function $poleMove(p, o')$ converts the polar coordinates of a point *p* into a new polar system that has a new pole at coordinate *o'* and can be summarized as in Algorithm 3.

Algorithm 3 poleMove

Input: point *p* with polar coordinate $[r_p, a_p]$, new pole *o'* with coordinate $[r_{o'}, a_{o'}]$

Output: new coordinates$[r_p', a_p']$ for point *p*

1: Convert $[r_p, a_p]$ to Cartesian coordinates:

$$[x_p, y_p] = [r_p * \cos(a_p), r_p * \sin(a_p)]$$

2: Convert $[r_{o'}, a_{o'}]$ to Cartesian coordinates:

$$[x_{o'}, y_{o'}] = [r_{o'} * \cos(a_{o'}), r_{o'} * \sin(a_{o'})]$$

3: Compute Cartesian coordinates of *p* with new origin:

$$[x_p', y_p'] = [x_p - x_{o'}, y_p - y_{o'}]$$

4: Convert $[x_p', y_p']$ to Polar Coordinates:

$$[r_p', a_p'] = \left[\sqrt{(x_p')^2 + (y_p')^2}, \tan^{-1}\left(\frac{y_p'}{x_p'}\right)\right]$$

Finally, $CCS(c_i)$ is a combination of both $CS1$ and $CS2$ and is determined by:

$$a_{CCS}(c_i) = max(a_{CS1}, a_{CS2})$$

$$r_{CCS}(c_i) = max(r_{CS1}, r_{CS2})$$

2) Compute the static layout. We can then calculate the shape of $DCS(v)$, which has the following radius and central angle:

$$r_{DCS}(v) = \max_{c_i \in children(v)} r_{CCS}(c_i)$$

$$a_{DCS}(v) = \sum_{c_i \in children(v)} \left(a_{CCS}(c_i) + \frac{s}{r_{DCS}(n)}\right)$$

For any given separating arc *s*, we can obtain the $DCS(v)$ for any node *v* in the GrapeTree recursively from tips to the root and estimate a new separating arc by comparing the maximum $a_{DCS}(v)$ to 2π. Although the optimal value of *s* can be obtained using dedicated solvers, the time consumption of those methods is intractable. Instead, we use an iteration method, which takes linear time in practice, to obtain an acceptable approximation for *s*.

The algorithm is written in pseudo-codes as:

Algorithm 4 static GrapeTree layout

Input: Tree $T(V, E)$, maximum number of trial X

Output: Optimal separating arc *s*

$s_0 = 2\pi/|V|$

$S = []$

for each trial *t* in $[0..X]$ **do**

    for each node v in $V_{preorder}$ **do**

        Compute $DCS(v)$

    $A_t = \max_{n \in N} a_{DCS(v)}$

    if $A_t \leq 2\pi$ **then**

        Append (s_t, A_t) to S

    $S_{t+1} = \frac{s_t * 2\pi}{A_t}$

$s = s_t$ in S that gives maximum value of A_t

Dynamic layout. The dynamic layout uses the force-directed layout in JavaScript D3 [S3] to implement a velocity Verlet integrator for simulating physical forces on particles [S4]. This algorithm sets the repulsion/attraction forces on nodes and an optimum distance (branch length) between nodes. However, along with other force-directed algorithms, repulsion between the nodes is favored, and hence the true branch lengths between nodes are rarely honored. As a result, identifying the true grouping of nodes can be difficult because two clusters may appear more distant than they should be according to their branch length. In addition, the force-directed algorithm rarely reaches an optimum, resulting in repeated screen updates after minor rotations of branch location. These features can result in poor performance, especially when a large number of nodes are involved. Once node positions are fixed after halting the force-directed algorithm, subsequent manual re-positioning of individual nodes is independent of the other node positions, and results in a distorted graph.

In order to overcome these limitations, we implemented an algorithm that corrects the branch lengths such that they are always true to their original values. Manual movements

of nodes are then restricted to those that do not falsify branch lengths, and child nodes are moved together with the parent node.

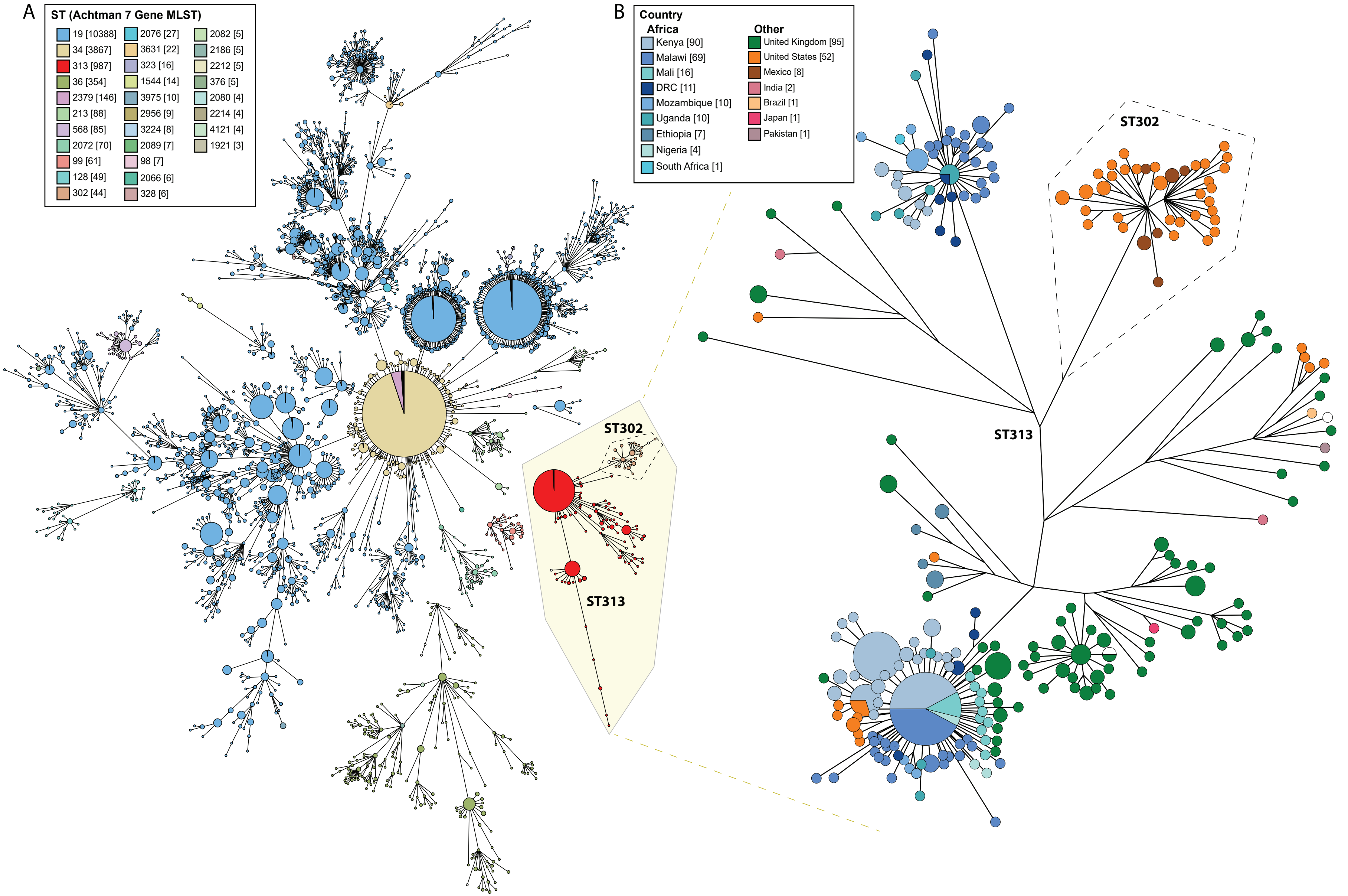

Supplemental Figure S1. Zooming in on a subset of data in GrapeTree EB. (A) MSTree V2 of cgMLST (3,002 loci) from 19,670 *Salmonella enterica* serovar Typhimurium genomes in EnteroBase. Nodes are colored by legacy 7-gene MLST ST. The yellow polygon highlights genomes from ST131 and ST302 which were selected for further analysis. An interactive version of this tree is available at http://bit.ly/2vjTn4I. All genomes, metadata and genotyping results are available to registered EnteroBase users at http://bit.ly/2IZSW1q. (B) Neighbour-Joining tree based on cgMLST of the selected genomes from part A, color-coded by country of isolation. An interactive version of this tree is available at http://bit.ly/2H8py8F. All genomes, metadata and genotyping results are available at http://bit.ly/2HrlwM1.

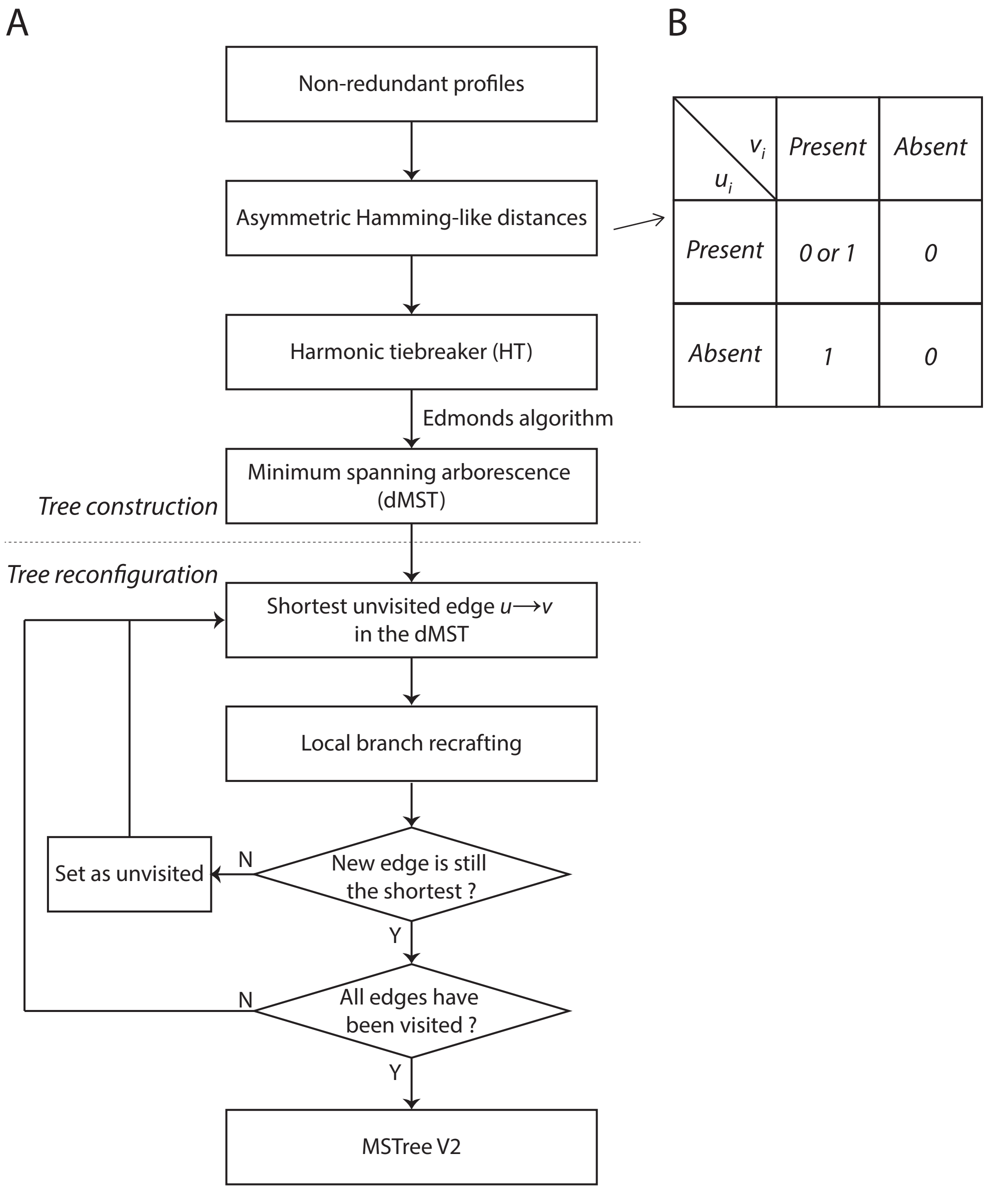

Supplemental Figure S2. MSTree V2 algorithm and asymmetric distances. A) Workflow involved in the MSTree V2 algorithm. Edmonds' algorithm (Edmonds 1967) was calculated using the Tarjan rapid implementation (Tarjan 1977) provided in the C++ Edmonds-alg open-source package by A. Tofigh (Tofigh 2009). B) Calculation of normalized, asymmetric Hamming-like distance for all possible combinations of allelic values in the calculation of the edge distance *d(u⟶ v)*. The genetic distance for each locus within an ST profile is 0 when the locus contains the same allele in *u* and *v*, and 1 when that locus contains distinct alleles. It is 1 when *v* contains an existing allele at that locus but the allele is missing in *u*, and is otherwise 0.

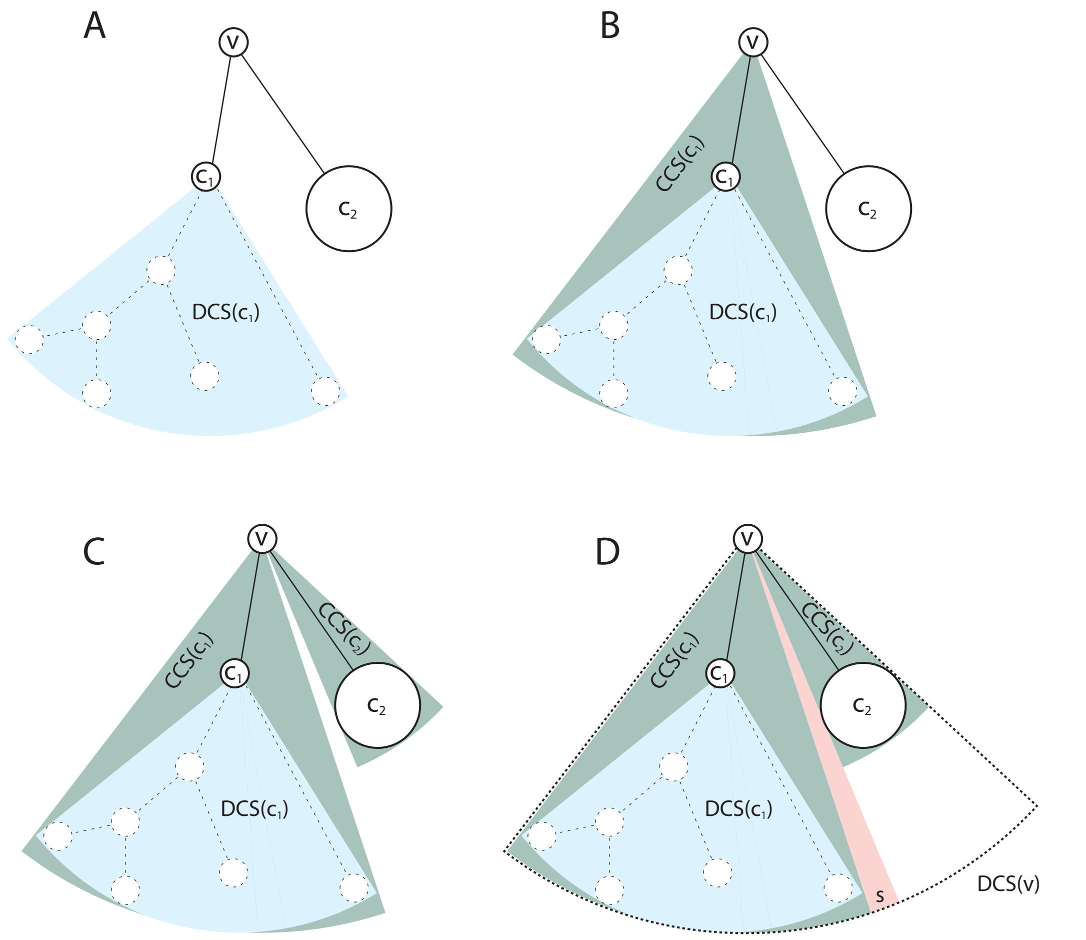

Supplemental Figure S3. Node layout algorithm in GrapeTree. The node layout depends on the calculation of descendent circular sectors (DCS) for each node. Node *v* has two children, nodes c_1 and c_2. All the descendants (dotted circles) of c_1 are encompassed within DCS(c_1) (cyan), whereas c_2 has no descendants. Node c_2 is larger than either *v* or c_1 because it contains multiple entries. A) Initial calculation. B) Child's circular sector CSS(c_1) (green, left) is drawn to include both c_1 and all its descendants. C) CSS(c_2) (green, right) is drawn to include large node c_2. D) DCS(*v*) is drawn as a summarized circular sector (dotted lines) that includes both CSS(c_1) and CSS(c_2) plus a separating arc *s* (pink) between them.

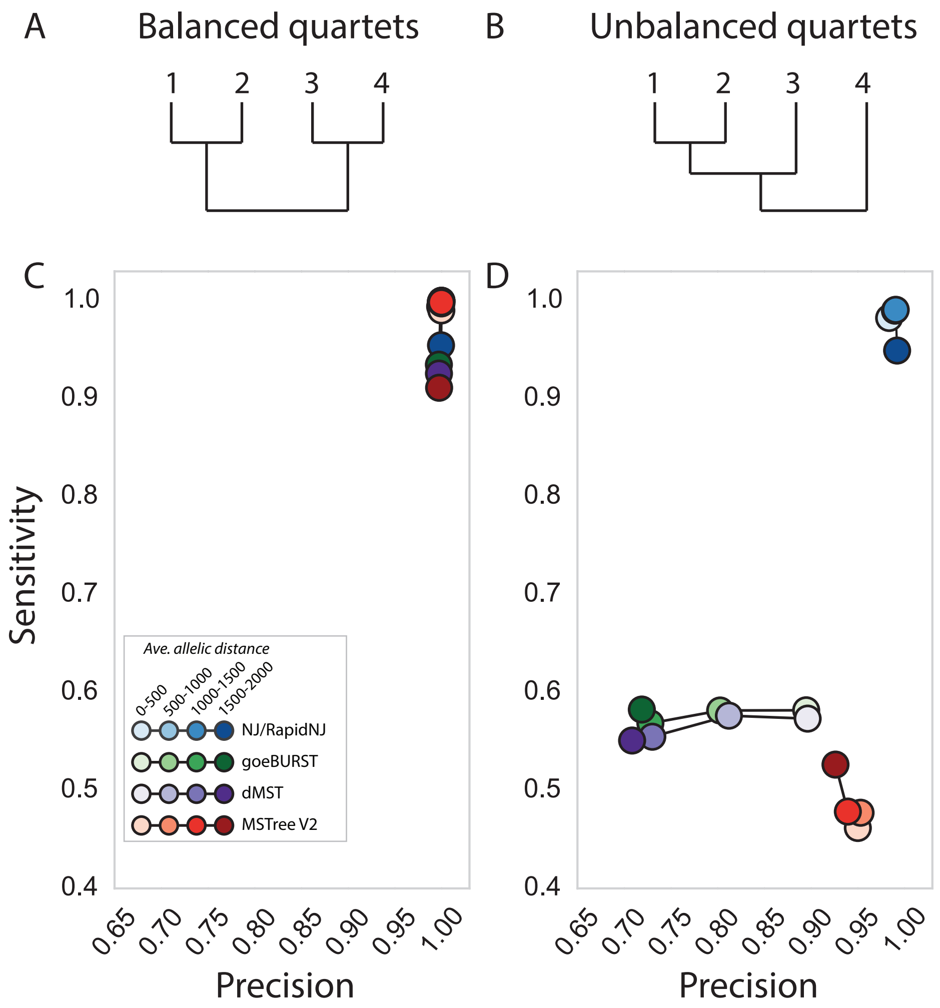

Supplemental Figure S4. Sensitivity *vs* precision for balanced and unbalanced quartets. Balanced and unbalanced quartets have identical topologies ((1,2),(3,4)). A quartet was scored as a balanced quartet if all pairwise distances satisfied $d_e > d_i$, where di denotes intra-group distances $d(1,2)$ and $d(3,4)$ and d_e denotes external distances between any other pair of nodes. All quartets that did not satisfy $d_e > d_i$ were scored as unbalanced quartets. A) Cartoon of balanced quartet. B) Cartoon of unbalanced quartet. C-D) Quartets were binned according to their average allelic distance as in Fig. 4. C) Performance for balanced quartets. All algorithms performed well. D) Performance for unbalanced quartets. NJ resolves unbalanced quartets accurately, but the sensitivity was <0.6 for all minimum spanning tree algorithms. Precision was low for goeBURST and dMST but remained high with MSTree V2 because local branch recrafting removes most inaccurate splits.

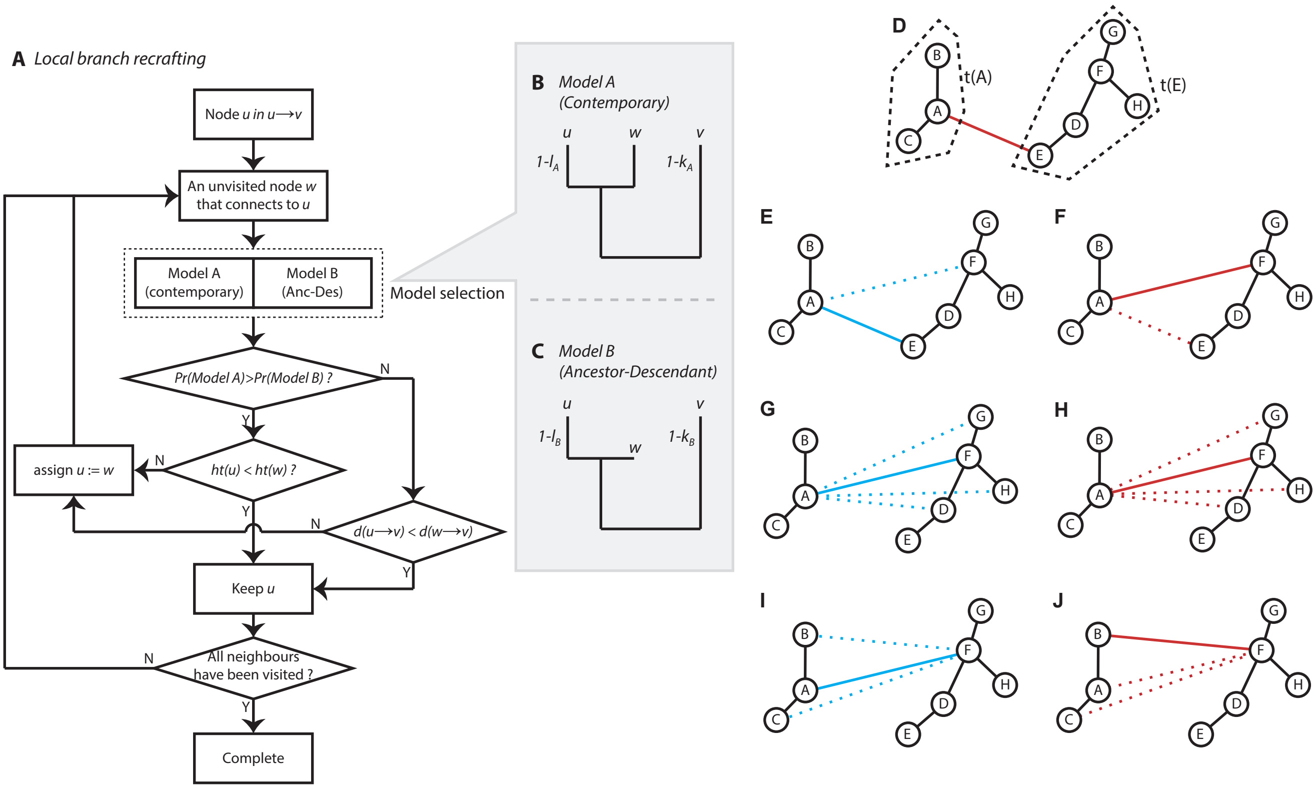

Supplemental Figure S5. **Flowchart and decision tree for branch recrafting.** A) Detailed workflow for the local branch recrafting of edge *u*⟶*v* dependent on the probabilities of models A and B for each previously unvisited node *w*. B) Cartoon of *Model A* in which nodes *u* and *w* are contemporary sisters that diverged from a hypothetical common ancestor. C) Cartoon of *Model B* in which node *w* is the direct ancestor of *u*. The probabilities of each model and the branch parameters l_A, k_A, l_B and k_B are calculated using equations 1 and 2 in Methods. (D-J) Cartoon of local branch recrafting. Solid lines indicate currently connected nodes while dotted branches indicate possible alternative connections. Blue lines in (E, G, I) are the branches involved in the model comparisons: Red lines in (F, H, J) show the outputs of the model comparisons. (D) The shortest branch (*A*⟶*E*) that connects two trees *t(A)* and *t(E)* according to Edmonds' algorithm. (E) (*A*⟶*E*) is compared with branch (*A*⟶*F*), where node F has the lowest harmonic average distance to other nodes. (F) (*A*⟶*F*) has higher probability. (G) (*A*⟶*F*) is compared with all the nodes that are directly connected with *F*. (H) (*A*⟶*F*) is still the most probable branch. (I-J) The same process is performed for tree *t(A)*, which results in (*B*⟶*F*) becoming the most probable branch.

Reference List

S1. Grivet S., Auber D., Domenger P.J., and Melancon G. (2006). Bubble tree drawing algorithm. In Computer Vision and Graphics, Wojciechowski K, Smolka B, Palus H, Kozera RS, Skarbek W, Noakes L, eds. Springer), pp. 633-641.

S2. Felsenstein J. (2004). Drawing trees. In Inferring phylogenies, (Sunderland, Massachusetts, US: Sinauer Associates, Inc.), pp. 573-584.

S3. Bostock M., Ogievetsky V., and Heer J. (2011). D3: Data-driven documents. IEEE Transactions on Visualization and Computer Graphics *17*: 2301-2309.

S4. Dwyer T. (2009). Scalable, versatile and simple constrained graph layout. Eurographics *28*.

S5. Edmonds J. (1967). Optimum branchings. J. Res. Nat. Bur. Standards *71B*: 233-240.

S6. Tarjan R.E. (1977). Finding optimum branchings. Networks *7*: 25-35.

S7. Tofigh, A. (2009). Optimum branchings and spanning aborescences. http://edmonds-alg.sourceforge.net/.
