# Supplementary material for: GrapeTree: visualization of core genomic relationships among 100,000 bacterial pathogens
Source: Genome Res. 2018 Sep;28(9):1395–404. doi: 10.1101/gr.232397.117 (PMC6120633; doi:10.1101/gr.232397.117)
Supplement: Supplemental Material [file supp_gr.232397.117_Supplemental_data_S3.zip › Supplemental_data/GrapeTree-codes/MSTree_holder.html]

×

Method

MSTreeV2
MSTree
Standard Neighbour Joining
RapidNJ
 Check memory usage

Close

|  
  |  

### GrapeTree

  

**Inputs/Outputs**

Load Files


---

Save GrapeTree
  
Save as Newick Tree  
Download SVG

**Tree Layout**

Original tree

  
 Static Redraw 
  
Centre Tree
  
 Show Tooltips


---

Drag Icon to Rotate: 

  
Zoom: 

**Node Style**

Colour By:
  

No category


---

 Show Labels


  
Font Size:
  
  


---


Node Size (%)

Kurtosis (%)

---

Highlight Label
  

  
 Show Pie Chart

**Branch Style**

Show Labels

  
Font Size:
  
  

Scaling (%)

Collapse Branches

Log Scale


---


For branches   
 longer than:

|  |  |  |
| --- | --- | --- |
| Display | Hide | Shorten |

**Rendering**

**Dynamic**

Selected Only

**Static**

Real Branch Length

**Context Menu**

GrapeTree

Metadata

Figure Legend
