# Supplementary material for: GrapeTree: visualization of core genomic relationships among 100,000 bacterial pathogens
Source: Genome Res. 2018 Sep;28(9):1395–404. doi: 10.1101/gr.232397.117 (PMC6120633; doi:10.1101/gr.232397.117)
Supplement: Supplemental Material [file supp_gr.232397.117_Supplemental_data_S3.zip › Supplemental_data/GrapeTree-codes/documentation/asymmetricDistances.pdf]

Calculation of asymmetric distances. In order to handle missing data correctly, MSTree V2 implements a directional measure based on normalized, asymmetric Hamming-like distances, $d(u \rightarrow v)$, between pairs of STs. This approach assumes that one of the pair of STs is the ancestor of the other, and treats missing data as deletions from the ancestor to the descendant.

Given a set of STs S and a profile $\pi(s)$ for each ST with a set of loci L, we define $d(u \rightarrow v)$ between an ordered pair of two STs (u, v) □ S as

$$d(u \rightarrow v) = \sum_{l \in L} \frac{\mathbb{1}_{\{(\pi_l(u) \neq \pi_l(v)) \wedge (\pi_l(v) \neq 0)\}}}{N_v}$$

with $N_v = \sum_{l \in L} 1_{\{\pi_l(v) \neq 0\}}$ and assuming 0 to be a missing value in all π. All possible values of these distances for each locus in the calculation of $d(u \rightarrow v)$ are illustrated in Supplemental Fig. S2B. Note that these distances do not form a metric, because $d(u \rightarrow v) \neq d(v \rightarrow u)$ when missing values are present. We can then define a fully connected graph $G(V, E)$ with $V = S$ and directed edges $(u \rightarrow v)$ □ E weighted by their distance. By analogy to a minimum spanning tree for undirected graphs, we compute a direct minimum spanning tree (dMST, also designated minimal spanning arborescence) on G in polynomial time with the Tarjan's rapid implementation (Tarjan 1977) of Edmond's algorithm (Edmonds 1967), using the Edmonds-alg package by A. Tofigh (Tofigh 2009).
