# Supplementary material for: GrapeTree: visualization of core genomic relationships among 100,000 bacterial pathogens
Source: Genome Res. 2018 Sep;28(9):1395–404. doi: 10.1101/gr.232397.117 (PMC6120633; doi:10.1101/gr.232397.117)
Supplement: Supplemental Material [file supp_gr.232397.117_Supplemental_data_S3.zip › Supplemental_data/GrapeTree-codes/documentation/branchRecrafting.pdf]

Local branch recrafting. Edmonds' algorithm attempts to minimize the sum of the edge lengths in the tree. However, the resulting dMST does not necessarily represent true phylogenetic relationships between strains because allelic distances do not always correlate with divergence time. We therefore implemented a subsequent branch optimization step that accounts for these discrepancies. Algorithm 1 gives an overview over the local branch recrafting (see also Supplemental Fig. S5), starting from the already computed dMST(*V*,*E*), where *E* is a distance matrix sorted in ascending order of allelic distances, and a forest *F* where each $u \in V$ is a single tree $t(u) \in F$.

Algorithm 1 Local branch recrafting

Input: Initial edge $(u \rightarrow v)$, Tree $t(u) \in F$, harmonic tiebreaker ht

Output: New edge $(u' \rightarrow v')$

1: Initialize $u' = u$

2: **for each** node $w \in TargetNodes\big(t(u')\big)$ **do**

3: $\quad P(M_A), P(M_B) = ModelSelection\big(d(u' \rightarrow w), d(w \rightarrow u'), d(u' \rightarrow v), d(w \rightarrow v)\big)$

4: $\quad$ **if** $P(M_A) \geq P(M_B) \wedge ht(u') > ht(w)$ **then**

5: $\quad\quad u' = w$

6: $\quad$ **else if** $P(M_A) < P(M_B) \wedge d(u' \rightarrow v) > d(w \rightarrow v)$ **then**

7: $\quad\quad u' = w$

8: Repeat (1 – 7) on v to obtain v'

9: Return $(u' \rightarrow v')$

Optimizations are applied to both ends of each edge iteratively as shown in Supplemental Fig. S5D. The $TargetNodes()$ function picks a subset of the nodes in tree $t(u)$ which are the centroids and the nodes that are directly connected to *u* (Supplemental Fig. S5D). The $ModelSelection()$ function compares the maximum likelihoods of two models M_A and M_B (Supplemental Fig. S5B,C). Here we describe only the model selection process for *u*. Given $d(u \rightarrow w)$, $d(w \rightarrow u)$, $d(u \rightarrow v)$ and $d(w \rightarrow v)$, when assuming $d(u \rightarrow v) \geq d(w \rightarrow v)$, the proportions of invariable sites in branches l_A, k_A, l_B and k_B satisfy:

$$\underset{0 \leq l_A \leq 1, 0 \leq k_A \leq 1}{\operatorname{argmax}} \log P(M_A | l_A, k_A)$$

$$= \underset{0 \leq l_A \leq 1, 0 \leq k_A \leq 1}{\operatorname{argmax}} \log P(u \rightarrow w | l_A) P(u \rightarrow v | l_A, k_A) P(w \rightarrow v | l_A, k_A)$$

$$= \underset{0 \leq l_A \leq 1, 0 \leq k_A \leq 1}{\operatorname{argmax}} |L| d(u \rightarrow w) \log(1 - l_A^2) + |L| \big(1 - d(u \rightarrow w)\big) \log(l_A^2)$$

$$+|L|d(u \to v)\log(1 - l_A k_A) + |L|\big(1 - d(u \to v)\big)\log(l_A k_A)$$

$$+|L|d(w \to v)\log(1 - l_A k_A) + |L|\big(1 - d(w \to v)\big)\log(l_A k_A) \quad (1)$$

$$\begin{aligned}
&\underset{0 \le l_B \le 1, 0 \le k_B \le 1}{\operatorname{argmax}} \log P(M_B | l_B, k_B) \\
&= \underset{0 \le l_B \le 1, 0 \le k_B \le 1}{\operatorname{argmax}} \log P(w \to u | l_B) P(u \to v | l_B, k_B) P(w \to v | l_B, k_B) \\
&= \underset{0 \le l_B \le 1, 0 \le k_B \le 1}{\operatorname{argmax}} |L| d(w \to u)\log(1 - l_B) + |L|\big(1 - d(w \to u)\big)\log(l_B) \\
&\quad +|L|d(u \to v)\log(1 - l_B k_B) + |L|\big(1 - d(u \to v)\big)\log(l_B k_B) \\
&\quad +|L|d(w \to v)\log(1 - k_B) + |L|\big(1 - d(w \to v)\big)\log(k_B) \quad (2)
\end{aligned}$$

where *L* is a set of loci in an MLST profile. Note that the direction of the distances between *u* and *w* are different in the two equations. Model *A* assumes u as the centroid node and adopts $d(u \to w)$ in equation (1) whereas model *B* treats w as the centroid and thus uses $d(w \to u)$. We further denote

$$x = 1 - \frac{\big(1 - d(w \to u)\big)\big(1 - d(w \to v)\big) + \big(1 - d(u \to v)\big)}{2}$$

Then the parameters in equations (1) and (2) are calculated as:

$$\begin{aligned}
l_A &= \sqrt{1 - d(u \to w)} \\
k_A &= \frac{1 - \frac{1}{2}\big(d(u \to v) + d(w \to v)\big)}{l_A} \\
l_B &= 1 + \frac{x d(w \to u)}{d(u \to v) - 2x} \\
k_B &= 1 + \frac{x d(w \to v)}{d(u \to v) - 2x}
\end{aligned}$$

These parameters can then be used to calculate $P(M_A)$ and $P(M_B)$ using equations (1) and (2).
