# Supplementary material for: GrapeTree: visualization of core genomic relationships among 100,000 bacterial pathogens
Source: Genome Res. 2018 Sep;28(9):1395–404. doi: 10.1101/gr.232397.117 (PMC6120633; doi:10.1101/gr.232397.117)
Supplement: Supplemental Material [file supp_gr.232397.117_Supplemental_data_S3.zip › Supplemental_data/GrapeTree-codes/documentation/tiebreak.pdf]

Harmonic tie-breaking. During the construction of an MSTree, Bionumerics or goeBURST choose between multiple co-optimal branches by tie-breaking according to the principles of eBURST (Feil *et al.* 2004) as summarized and extended by (Francisco *et al.* 2009). The eBURST approach presumes that a clonal complex (lineage) is founded by a founder genotype, and that genetic variants of that founder reflect the progressive accumulation of additional variations over time. A further implicit belief is that the number of variants decreases with distance from the founder genotype, such that the founder is equated with the central genotype with the greatest number of single locus variants, and edges between nodes are ordered based on their allelic distances. In case of a tie for directionality of connections, the founder status is assigned to the node with the greater number of single locus variants, double locus variants, triple locus variants, and/or number of strains assigned to that ST.

At cgMLST levels of resolution, the founder genotype may not be present in a comparison, which renders the eBURST model inappropriate for tie-breaking. Instead of depending on the preconceived properties of a theoretical founder genotype, MSTree V2 simply chooses central nodes between multiple co-optimal branches on the basis of the harmonic mean of allelic distances.

We define a *centroid* genotype, which is the genotype for any given population that has the smallest average allelic distance to all other genotypes in the same population. The harmonic mean of the allelic distances is used rather than an arithmetic mean in order to give higher weights to variants with smaller allelic distances to other STs. In a fully connected graph *G*(*V,E*) as defined above, we define the harmonic mean *ht*(*u*) of allelic distances for any node $u \in V$ to other nodes as:

$$ht(u) = \left(\frac{\sum_{\substack{v \in V, \\ u \neq v}} d(u \rightarrow v)^{-1}}{|V| - 1} \right)^{-1}$$

All directed edges $d(u \rightarrow v)$ are ordered in ascending order according to *ht*(*u*), with the frequency of occurrence of *u* as the final tie-break. This ordering results in a unique and optimal dMST with Edmonds' algorithm. Furthermore, since we have a fully connected graph and *d* satisfies the triangular inequality, the length of the shortest (geodesic) path between any two vertices *u* and *v* is given by $d(u \rightarrow v)$.

We note that $ht(u)^{-1}$ is also known as 'closeness centrality' in network science (Newman 2010). Closeness centrality is usually defined for unweighted graphs as the inverse of the mean distance between vertices. But some interesting properties arise when it is defined in

our sense as the inverse of the harmonic mean distance between vertices: $ht(u)^{-1}$ gives more weight to vertices that are close to the vertex of interest than to those far away, and it can also naturally deal with disconnected components.
