# Supplementary material for: GrapeTree: visualization of core genomic relationships among 100,000 bacterial pathogens
Source: Genome Res. 2018 Sep;28(9):1395–404. doi: 10.1101/gr.232397.117 (PMC6120633; doi:10.1101/gr.232397.117)
Supplement: Supplemental Material [file supp_gr.232397.117_Supplemental_data_S3.zip › Supplemental_data/GrapeTree-codes/documentation/developer/classes.list.html]

Documentation Classes


Documentation

- Classes
  - D3BaseTree
  - D3MSTree
- Global
  - Global

# Classes

## 

### Classes

D3BaseTree


D3MSTree

×

#### Search results

Close

Documentation generated by JSDoc 3.4.3
on 2017-06-01T10:03:07+01:00
using the DocStrap template.
