# Supplementary material for: GrapeTree: visualization of core genomic relationships among 100,000 bacterial pathogens
Source: Genome Res. 2018 Sep;28(9):1395–404. doi: 10.1101/gr.232397.117 (PMC6120633; doi:10.1101/gr.232397.117)
Supplement: Supplemental Material [file supp_gr.232397.117_Supplemental_data_S3.zip › Supplemental_data/GrapeTree-codes/documentation/developer/D3BaseTree.html]

Documentation Class: D3BaseTree


Documentation

- Classes
  - D3BaseTree
  - D3MSTree
- Global
  - Global

# Class: D3BaseTree

## D3BaseTree

---

#### new D3BaseTree(element\_id, metadata, height, width)
:   The base class for trees

    ##### Parameters:

    | Name | Type | Description |
    | --- | --- | --- |
    | `element_id` | string | The id of the container for the tree |
    | `metadata` | object | (optional ) An object describing the trees metadata see D3BaseTree#addMetadata |
    | `height` | integer | the initial height. The container will be reisized to this height. If absent, the height of the container will be used |
    | `width` | integer | the initial width. The container will be reisized to this width If absent, the width of the container will be used |

### Methods

---

#### addMetadata(metadata)
:   Adds metadata to the tree

    ##### Parameters:

    | Name | Type | Description |
    | --- | --- | --- |
    | `metadata` | object | An object containing id to a list of key value pairs.If there is a one to one relationship beteween the nodes and metadata, then the id should correspond to the node id e,g,  ``` { 	node_a:{year:"1987",color:"red"}, 	node_b:{.....} 	,.... } ```  If a node reprsents several entities e.g.an ST has several strins, then an ID property is required, which is the ID of node e.g.  ``` { 	strain_a:{year:"1988",virulence:"high",ID:"ST27"}, 	strain_b:{year:"1987",virulence:"low",ID:"ST27"}, 	strain_c:{year:"1989",virulence:"medium",ID:"ST28"}, 	.... } ```  If the id already exists, than new properties will be added or existing ones altered e.g.  ``` { strain_a:{year:"1999",new_category:"value1"} } ``` |

---

#### downloadSVG()
:   Downloads the current tree in svg format

---

#### getMetadata()
:   Retreives metadata

    ##### Returns:

    An object containing id to a list of key value pairs see D3BaseTree#addMetadata

    Type
    :   object

---

#### resize()
:   Resizes the tree components based on the size of the container
    This method is automtically called if the window is resized,
    but should be called if the container is resized manually

---

#### searchMetadata(keyword)
:   Searches the node names (ids) and all metadata values associated
    with the node for the keyword

    ##### Parameters:

    | Name | Type | Description |
    | --- | --- | --- |
    | `keyword` | string | The word to use for the search |

    ##### Returns:

    All the node ids where the keyword was found

    Type
    :   list

---

#### setColour(category, value, colour)
:   Sets the colour for a value in a category e.g. setColour("Country","France","blue")

    ##### Parameters:

    | Name | Type | Description |
    | --- | --- | --- |
    | `category` | string | The name of the field (category) |
    | `value` | string | The name of the value |
    | `colour` | string | The colour to set (usual fomration) |

---

#### setScale(scale, relative)
:   Sets the scale (size of the tree)

    ##### Parameters:

    | Name | Type | Description |
    | --- | --- | --- |
    | `scale` | float | The scale to set e.g 2 |
    | `relative` | boolean | If true than the current scale will be multiplied by the scale parameter e.g 0.5,true would halve the current size of the tree |

---

#### setTranslate(scale)
:   Sets the translate (offset of the tree)

    ##### Parameters:

    | Name | Type | Description |
    | --- | --- | --- |
    | `scale` | array | An array containing the x,y offsets eg [30,-100] |

---

#### showLegend(show)
:   Hide/Show the menu

    ##### Parameters:

    | Name | Type | Description |
    | --- | --- | --- |
    | `show` | boolean | If true the menu will be shown |

×

#### Search results

Close

Documentation generated by JSDoc 3.4.3
on 2017-06-01T10:03:07+01:00
using the DocStrap template.
