# Supplementary material for: GrapeTree: visualization of core genomic relationships among 100,000 bacterial pathogens
Source: Genome Res. 2018 Sep;28(9):1395–404. doi: 10.1101/gr.232397.117 (PMC6120633; doi:10.1101/gr.232397.117)
Supplement: Supplemental Material [file supp_gr.232397.117_Supplemental_data_S3.zip › Supplemental_data/GrapeTree-codes/documentation/developer/D3MSTree.html]

Documentation Class: D3MSTree


Documentation

- Classes
  - D3BaseTree
  - D3MSTree
- Global
  - Global

# Class: D3MSTree

## D3MSTree

---

#### new D3MSTree(element\_id, data, callback, height, width)
:   ##### Parameters:

    | Name | Type | Description |
    | --- | --- | --- |
    | `element_id` | string | The id of the container for the tree |
    | `data` | InitialData | An object containing the tree's data |
    | `callback` | function | The function to be called when the set up is finished (optional). The callback is passed the tree object and a message describing the state of initialisation. The message will be 'complete' when the tree is finished |
    | `height` | integer | the initial height (optional) |
    | `width` | integer | the initial width (optional) |

### Extends

- D3BaseTree

### Methods

---

#### addLinkClickedListener(func)
:   Adds a listener to the link, which is called when the link is clicked

    ##### Parameters:

    | Name | Type | Description |
    | --- | --- | --- |
    | `func` | function | A callback which is called when a link is clicked The functions is supplied the link e.g. addLinkClickedListener(function(link){...}); |

---

#### addLinkOutListener(func)
:   Adds a listener to the node, which is called when the mouse leaves the link

    ##### Parameters:

    | Name | Type | Description |
    | --- | --- | --- |
    | `func` | function | A callback which is called when the mouse leaves the link The functions is supplied the link e.g. addLinkOverListener(function(link){...}); |

---

#### addLinkOverListener(func)
:   Adds a listener to the node, which is called when the mouse is over the link

    ##### Parameters:

    | Name | Type | Description |
    | --- | --- | --- |
    | `func` | function | A callback which is called when a link is mouse overed The functions is supplied the link e.g. addLinkOverListener(function(link){...}); |

---

#### addMetadata(metadata)
:   Adds metadata to the tree

    ##### Parameters:

    | Name | Type | Description |
    | --- | --- | --- |
    | `metadata` | object | An object containing id to a list of key value pairs.If there is a one to one relationship beteween the nodes and metadata, then the id should correspond to the node id e,g,  ``` { 	node_a:{year:"1987",color:"red"}, 	node_b:{.....} 	,.... } ```  If a node reprsents several entities e.g.an ST has several strins, then an ID property is required, which is the ID of node e.g.  ``` { 	strain_a:{year:"1988",virulence:"high",ID:"ST27"}, 	strain_b:{year:"1987",virulence:"low",ID:"ST27"}, 	strain_c:{year:"1989",virulence:"medium",ID:"ST28"}, 	.... } ```  If the id already exists, than new properties will be added or existing ones altered e.g.  ``` { strain_a:{year:"1999",new_category:"value1"} } ``` |

    Inherited From:
    :   - D3BaseTree#addMetadata

    Overrides:
    :   - D3BaseTree#addMetadata

---

#### addNodeClickedListener(func)
:   Adds a listener to the node, which is called when the node is clicked

    ##### Parameters:

    | Name | Type | Description |
    | --- | --- | --- |
    | `func` | function | A callback which is called when a node is clicked The functions is supplied the actual node and a list of IDs associated with the node e.g. addNodeClickedListener(function(node,ids){...}); |

---

#### addSegmentOutListener(func)
:   Adds a listener to the node segment, which is called when the mouse leaves the segment

    ##### Parameters:

    | Name | Type | Description |
    | --- | --- | --- |
    | `func` | function | A callback which is called when the mouse leaves the segment The functions is supplied the segment e.g. addLinkOverListener(function(segment){...}); |

---

#### addSegmentOverListener(func)
:   Adds a listener to the node segment, which is called when the mouse is over the segment

    ##### Parameters:

    | Name | Type | Description |
    | --- | --- | --- |
    | `func` | function | A callback which is called when a segment is mouse overed The functions is supplied the segment e.g. addLinkOverListener(function(segment){...}); |

---

#### alterCharge(amount)
:   If nodes are unfixed (i.e. they are being positioned by the force algorithm)
    the supplied value specifies the repelling force between each node

    ##### Parameters:

    | Name | Type | Description |
    | --- | --- | --- |
    | `amount` | number | the repelling force between each node (or attraction if amount is posotive) |

---

#### changeCategory(category)
:   Changes the category displayed

    ##### Parameters:

    | Name | Type | Description |
    | --- | --- | --- |
    | `category` | string | The category to display. If no category is given then the node IDs will be displayed |

---

#### collapseNodes(max\_distance)
:   Collapses the nodes in the tree. If any link between two nodes is less than or
    equal to max\_distance, the nodes will be collapsed into one.

    ##### Parameters:

    | Name | Type | Description |
    | --- | --- | --- |
    | `max_distance` | number | All nodes equal or below this distance apart will be collapsed |

---

#### downloadSVG()
:   Downloads the current tree in svg format

    Inherited From:
    :   - D3BaseTree#downloadSVG

---

#### fixAllNodes()
:   This fixes all nodes and stops the 'force' algorithm from updating
    their position

---

#### getLayout()
:   Returns the data describing the current tree's layout

    ##### Returns:

    layout \_data Data describing the layout of the tree

    Type
    :   LayoutData

---

#### getMetadata()
:   Retreives metadata

    Inherited From:
    :   - D3BaseTree#getMetadata

    ##### Returns:

    An object containing id to a list of key value pairs see D3BaseTree#addMetadata

    Type
    :   object

---

#### getSelectedIDs()
:   Returns all the selected IDs of the selected node

    ##### Returns:

    All the selected IDs, these may be the ids of the nodes or
    if the node is assocaited with items, the ID of all the items in the node

    Type
    :   list

---

#### getTreeAsObject()
:   Returns the tree as an object InitialData, suitiable for use in the trees constructor

    ##### Returns:

    An object descibing the tree

    Type
    :   InitialData

---

#### highlightIDs(IDs, color)
:   All nodes which contain metadata that has ID in is the supplied list
    will have a large halo around them.

    ##### Parameters:

    | Name | Type | Description |
    | --- | --- | --- |
    | `IDs` | list | A list of IDs to higlight, the IDs will be either be the IDs of items associated with a node or the id of the node (if a one to one relationship) Even if only one ID is present in the node, then the whole node will be highlighted. |
    | `color` | string | The color of the halo (default yellow) |

---

#### highlightNodes(IDs, color)
:   All nodes with the id in supplied list will have a large yellow
    halo around them.

    ##### Parameters:

    | Name | Type | Description |
    | --- | --- | --- |
    | `IDs` | list | A list of nodes IDs to higlight, |
    | `color` | string | The color of the halo (default yellow) |

---

#### resetLinkLengths()
:   Resets all link lenghts to accurately reflect the original value.

---

#### resize()
:   Resizes the tree components based on the size of the container
    This method is automtically called if the window is resized,
    but should be called if the container is resized manually

    Inherited From:
    :   - D3BaseTree#resize

---

#### searchMetadata(keyword)
:   Searches the node names (ids) and all metadata values associated
    with the node for the keyword

    ##### Parameters:

    | Name | Type | Description |
    | --- | --- | --- |
    | `keyword` | string | The word to use for the search |

    Inherited From:
    :   - D3BaseTree#searchMetadata

    ##### Returns:

    All the node ids where the keyword was found

    Type
    :   list

---

#### setColour(category, value, colour)
:   Sets the colour for a value in a category e.g. setColour("Country","France","blue")

    ##### Parameters:

    | Name | Type | Description |
    | --- | --- | --- |
    | `category` | string | The name of the field (category) |
    | `value` | string | The name of the value |
    | `colour` | string | The colour to set (usual fomration) |

    Inherited From:
    :   - D3BaseTree#setColour

---

#### setHideLinkLength(max\_length)
:   Hides all links which have a length above the value supplied

    ##### Parameters:

    | Name | Type | Description |
    | --- | --- | --- |
    | `max_length` | number | The length above which links will be hidden |

---

#### setIndividualLinkLength(link, length)
:   Alters the length of the link to the supplied value

    ##### Parameters:

    | Name | Type | Description |
    | --- | --- | --- |
    | `link` | object | The link object to alter |
    | `length` | number | The new length of thr link |

---

#### setLayout(layout)
:   Updates the tree with the supplied data. Any paramater not supplied will be default

    ##### Parameters:

    | Name | Type | Description |
    | --- | --- | --- |
    | `layout` | LayoutData | \_data - An object describing the layout of the tree |

---

#### setLinkFontSize(The)
:   Sets the font size of the links

    ##### Parameters:

    | Name | Type | Description |
    | --- | --- | --- |
    | `The` | integer | font size (in pixels) |

---

#### setLinkLength(max\_length)
:   Sets the relative length of the all links.

    ##### Parameters:

    | Name | Type | Description |
    | --- | --- | --- |
    | `max_length` | integer | This specifies length of longest link in pixels All other links will be scaled between this value and 1 |

---

#### setMaxLinkLength(amount)
:   Sets the maximum length of any link. Any links over the value supplied
    will be reduced to the maximum length and displayed as dotted lines

    ##### Parameters:

    | Name | Type | Description |
    | --- | --- | --- |
    | `amount` | number | The maximum link length |

---

#### setNodeFontSize(node)
:   Sets the size of node labels in pixels

    ##### Parameters:

    | Name | Type | Description |
    | --- | --- | --- |
    | `node` | number | label font size in pixels |

---

#### setNodeSize(node\_size)
:   Sets the base node size. The radius of the node is calculated by the
    log of the number of items represented by the node multipled by base node size

    ##### Parameters:

    | Name | Type | Description |
    | --- | --- | --- |
    | `node_size` | number | The base node size |

---

#### setNodeText(value)
:   Sets the label to display on the node.

    ##### Parameters:

    | Name | Type | Description |
    | --- | --- | --- |
    | `value` | string | The name of a the category whose values will be displayed. if 'node\_id' is given then the node id will be displayed. |

---

#### setRelativeNodeSize(factor)
:   Sets the relative node size. Default is 0.5, The smaller the number, the smaller
    nodes will be that represent many items

    ##### Parameters:

    | Name | Type | Description |
    | --- | --- | --- |
    | `factor` | number | The size\_power value used in node size calculation |

---

#### setScale(scale, relative)
:   Sets the scale (size of the tree)

    ##### Parameters:

    | Name | Type | Description |
    | --- | --- | --- |
    | `scale` | float | The scale to set e.g 2 |
    | `relative` | boolean | If true than the current scale will be multiplied by the scale parameter e.g 0.5,true would halve the current size of the tree |

    Inherited From:
    :   - D3BaseTree#setScale

---

#### setTranslate(scale)
:   Sets the translate (offset of the tree)

    ##### Parameters:

    | Name | Type | Description |
    | --- | --- | --- |
    | `scale` | array | An array containing the x,y offsets eg [30,-100] |

    Inherited From:
    :   - D3BaseTree#setTranslate

---

#### showIndividualSegments(true)
:   If true each individual item in the node will have its own 'wedge' even if it
    is the same category (although it is the same colour). This useful to guage the
    size of the node, but if nodes represent 100's of items, will slow down the
    rendering of the tree

    ##### Parameters:

    | Name | Type | Description |
    | --- | --- | --- |
    | `true` | boolean | or false |

---

#### showLegend(show)
:   Hide/Show the menu

    ##### Parameters:

    | Name | Type | Description |
    | --- | --- | --- |
    | `show` | boolean | If true the menu will be shown |

    Inherited From:
    :   - D3BaseTree#showLegend

---

#### showLinkLabels(show)
:   Determines whether to show distance labels on links

    ##### Parameters:

    | Name | Type | Description |
    | --- | --- | --- |
    | `show` | boolean | true or false |

---

#### showNodeLabels(show)
:   Determines wheteher node labels will be present

    ##### Parameters:

    | Name | Type | Description |
    | --- | --- | --- |
    | `show` | boolean | Either true or false |

---

#### unfixSelectedNodes(all)
:   This will cause all nodes to be acted upon by the 'force' algorithm
    and will alter their position. Nodes will spread out and link lengths
    may no longer be accurate

    ##### Parameters:

    | Name | Type | Description |
    | --- | --- | --- |
    | `all` | boolean | If true all nodes will be released, otherwise just the selected ones |

×

#### Search results

Close

Documentation generated by JSDoc 3.4.3
on 2017-06-01T10:03:07+01:00
using the DocStrap template.
