# Supplementary material for: GrapeTree: visualization of core genomic relationships among 100,000 bacterial pathogens
Source: Genome Res. 2018 Sep;28(9):1395–404. doi: 10.1101/gr.232397.117 (PMC6120633; doi:10.1101/gr.232397.117)
Supplement: Supplemental Material [file supp_gr.232397.117_Supplemental_data_S3.zip › Supplemental_data/GrapeTree-codes/documentation/developer/global.html]

Documentation Global


Documentation

- Classes
  - D3BaseTree
  - D3MSTree
- Global
  - Global

# Global

## 

### Type Definitions

---

#### InitialData
:   An object describing the tree. Either nodes and links or nwk or nexus
    are the only required properties,the rest will be set as default

    ##### Type:

    - Object

    ##### Properties:

    | Name | Type | Description |
    | --- | --- | --- |
    | `nodes` | list | A list of node names ['ST131','ST11'] |
    | `links` | list | A list of objects containing source,target and distance , where source and targets are indexes to the nodes list e.g. [{source:0,target:1,distance:10},...] |
    | `nwk` | string | A tree in newick format |
    | `nexus` | string | A tree in nexus format |
    | `layout_algorithm` | string | The initial algorithm to work out node positions. Can be either 'force' or 'greedy'. Default is 'greedy' |
    | `layout_data` | LayoutData | Data describing the layout. If none is supplied default values will be supplied and the specified algorithm will calculate the initial layout |
    | `metadata` | object | The metadata for the tree - see D3MSTree#addMetadata |
    | `initial_category` | string | The initial category to display in the tree - see D3MSTree#changeCategory |

---

#### LayoutData
:   ##### Type:

    - Object

    ##### Properties:

    | Name | Type | Description |
    | --- | --- | --- |
    | `node_positions` | object | A dictionary of node id to an array of x,y co-ordinate e.g. {node\_a:[23,76],node\_b:[65,75]} |
    | `node_links` | object | a dictionary of the following  - **max\_link\_length** The maxiumum length of a link . Any link over this distance   will be corrected to this length and displayed as dotted. - **max\_link\_scale** Controls the length that each link is displayed. The links will be   scaled (in pixels) between this value and 0 - **log\_link\_scale** If true - link length will be altered to the power of 0.8. Default is false - **link\_font\_size** The size in pixels of link labels. Default is 10 - **show\_link\_labels** Determines whether the distance labels on links are present. Default is false - **hide\_link\_length** All links over this length will not be displayed. Default is infinity - **base\_node\_size**  The base size of the nodes. Default is 10 - **show\_node\_labels** Determines whether node labels are present. Default true - **node\_font\_size** Controls the size (in pixels) of node labels. Default is 14 - **node\_text\_value** The category to display on the node label. Default is none (node id is shown) - **size\_power** Controls the size of the nodes. Nodes, will have a radius which equal to number   of items associated with the node to the power of size\_power multiplied by base\_node\_size   Default value is 0.5 - **show\_individual\_segments** If true then every single component of the node will have its own 'wedge'   even those in the same category (although they will be the same colour). Default is false. - **node\_collapsed\_value** Nodes connecting by links with a distance equal or below this value   will be collapsed. Default is 0 - **custom\_colours** A dictionary of categories to value/colour pairs e.g.   {category1:{value\_1:"blue",value\_2:"red",..},"category\_2":{....},...}- **scale** The scale factor (1.0 being normal size)   - **translate** The offset of the tree an array of x.y co-ordinate e.g. [30.-20] |

×

#### Search results

Close

Documentation generated by JSDoc 3.4.3
on 2017-06-01T10:03:07+01:00
using the DocStrap template.
