# Supplementary material for: GrapeTree: visualization of core genomic relationships among 100,000 bacterial pathogens
Source: Genome Res. 2018 Sep;28(9):1395–404. doi: 10.1101/gr.232397.117 (PMC6120633; doi:10.1101/gr.232397.117)
Supplement: Supplemental Material [file supp_gr.232397.117_Supplemental_data_S3.zip › Supplemental_data/GrapeTree-codes/documentation/developer/index.html]

Documentation Index


Documentation

- Classes
  - D3BaseTree
  - D3MSTree
- Global
  - Global

# D3MSTree (GrapeTree)

### For the Impatient

```
<html>
     
     
     
     

    <div id ='tree-holder' style='height:300px;width:400px;border-style:solid'></div>

     
</html>
```

×

#### Search results

Close

Documentation generated by JSDoc 3.4.3
on 2017-06-01T10:03:07+01:00
using the DocStrap template.
