# Supplementary material for: GrapeTree: visualization of core genomic relationships among 100,000 bacterial pathogens
Source: Genome Res. 2018 Sep;28(9):1395–404. doi: 10.1101/gr.232397.117 (PMC6120633; doi:10.1101/gr.232397.117)
Supplement: Supplemental Material [file supp_gr.232397.117_Supplemental_data_S3.zip › Supplemental_data/GrapeTree-codes/static/js/SlickGrid/examples/example-autotooltips.html]

SlickGrid plugin example: AutoTooltips


|  |  |
| --- | --- |
|  | Use jQuery UI tooltips  Demonstrates:  - AutoTooltips plugin - Optional stylable jQueryUI tooltips.  Instructions: Resize the columns until see ellipsis in column or header. Hover over cell to see tooltip.  For jQueryUI tooltips, call the .tooltips() function on the document to convert all tooltips to jQueryUI. To style jQueryUI tooltips, use the generic jQueryUI class 'ui-tooltip', or pass in a class name as an option. Usage:  ``` plugin = new Slick.AutoTooltips(pluginOptions); grid.registerPlugin(plugin); grid.render(); ```   jQueryUI Tooltips:   ``` $( document ).tooltip( { tooltipClass: "my-tooltip-class" } ); ```  View Source:  - View the source for this example on Github |
