# Supplementary material for: GrapeTree: visualization of core genomic relationships among 100,000 bacterial pathogens
Source: Genome Res. 2018 Sep;28(9):1395–404. doi: 10.1101/gr.232397.117 (PMC6120633; doi:10.1101/gr.232397.117)
Supplement: Supplemental Material [file supp_gr.232397.117_Supplemental_data_S3.zip › Supplemental_data/GrapeTree-codes/static/js/SlickGrid/examples/example-bootstrap-3-header.html]

SlickGrid example: Basic Grid with Bootstrap 3 css


|  |  |
| --- | --- |
|  | Demonstrates:  - Slickgrid with Bootstrap 3 css (bootstrap.css). This imposes 'box-sizing: border-box;' on all elements in the page.  View Source:  - View the source for this example on Github |
