# Supplementary material for: GrapeTree: visualization of core genomic relationships among 100,000 bacterial pathogens
Source: Genome Res. 2018 Sep;28(9):1395–404. doi: 10.1101/gr.232397.117 (PMC6120633; doi:10.1101/gr.232397.117)
Supplement: Supplemental Material [file supp_gr.232397.117_Supplemental_data_S3.zip › Supplemental_data/GrapeTree-codes/static/js/SlickGrid/examples/example-explicit-initialization.html]

SlickGrid example: Explicit grid initialization


|  |  |
| --- | --- |
|  | Demonstrates: The container which the grid is being created in needs to be in the DOM and participate in layout (can be 'visibility:hidden' but not 'display:none') in order for SlickGrid to be able to make certain measurements and initialize event listeners. Normally, this is done when a SlickGrid instance is being created. Optionally, you can defer the initialization until the above condition is met and call the **grid.init()** method explicitly. To use explicit initialization, set the **explicitInitialization** option to true.    This example demonstrates creating a SlickGrid inside a detached element and calling **init()** explicitly when the element is added to the DOM. View Source:  - View the source for this example on Github |
