# Supplementary material for: GrapeTree: visualization of core genomic relationships among 100,000 bacterial pathogens
Source: Genome Res. 2018 Sep;28(9):1395–404. doi: 10.1101/gr.232397.117 (PMC6120633; doi:10.1101/gr.232397.117)
Supplement: Supplemental Material [file supp_gr.232397.117_Supplemental_data_S3.zip › Supplemental_data/GrapeTree-codes/static/js/SlickGrid/examples/example-external-headersort.html]

External Header Sort


|  |  |
| --- | --- |
|  | Demonstrates:  - external column sort initialisation   - uses the options hook of passing a function (rather than true/false/undefined) as the `enableColumnReorder` option. This function will be used to set up the column reordering.  - the function prototype is: `function(grid, $headers, headerColumnWidthDiff, setColumns, setupColumnResize, columns, getColumnIndex, uid, trigger)` |
