# Supplementary material for: GrapeTree: visualization of core genomic relationships among 100,000 bacterial pathogens
Source: Genome Res. 2018 Sep;28(9):1395–404. doi: 10.1101/gr.232397.117 (PMC6120633; doi:10.1101/gr.232397.117)
Supplement: Supplemental Material [file supp_gr.232397.117_Supplemental_data_S3.zip › Supplemental_data/GrapeTree-codes/static/js/SlickGrid/examples/example-grouping.html]

SlickGrid example: Grouping


SlickGrid

**Options:**


---

Show tasks with % at least:   
  
50 rows
50k rows
500k rows


---

Clear grouping
  
Group by duration & sort groups by value
  
Group by duration & sort groups by count
  
Group by duration & sort groups by count, aggregate
collapsed
  
  
Group by duration then effort-driven
  
Group by duration then effort-driven then percent.
  
  
Collapse all groups
  
Expand all groups

---

## Demonstrates:

- Fully dynamic and interactive multi-level grouping with filtering and aggregates over **50'000** items  
  Each grouping level can have its own aggregates (over child rows, child groups, or all descendant rows).  
  Personally, this is just the coolest slickest thing I've ever seen done with DHTML grids!

## View Source:

- View the source for this example on Github
