# Supplementary material for: GrapeTree: visualization of core genomic relationships among 100,000 bacterial pathogens
Source: Genome Res. 2018 Sep;28(9):1395–404. doi: 10.1101/gr.232397.117 (PMC6120633; doi:10.1101/gr.232397.117)
Supplement: Supplemental Material [file supp_gr.232397.117_Supplemental_data_S3.zip › Supplemental_data/GrapeTree-codes/static/js/SlickGrid/examples/example-jquery-accordion.html]

SlickGrid example: jQuery Accordion with a grid in each pane


|  |  |
| --- | --- |
| Section 1  Section 2 | Demonstrates:  - jQuery Accordion containing a grid in each pane  View Source:  - View the source for this example on Github |
