# Supplementary material for: GrapeTree: visualization of core genomic relationships among 100,000 bacterial pathogens
Source: Genome Res. 2018 Sep;28(9):1395–404. doi: 10.1101/gr.232397.117 (PMC6120633; doi:10.1101/gr.232397.117)
Supplement: Supplemental Material [file supp_gr.232397.117_Supplemental_data_S3.zip › Supplemental_data/GrapeTree-codes/static/js/SlickGrid/examples/example-optimizing-dataview.html]

SlickGrid example: Optimizing DataView


SlickGrid

**Search:**


---

Show tasks with % at least:

  

This page demonstrates various techniques for optimizing DataView performance
for large client-side datasets. This page displays an interactive grid with
500'000 rows with real-time filtering.  
This is achieved by:

- Inlining filter function to cut down on the cost of function calls.
- Providing hints to indicate whether a filtering operation will result in
  narrowing or expanding scope or whether the scope is unchanged.
- Providing a range of rows for which onRowsChanged even should be fired.

## View Source:

- View the source for this example on Github
