# Supplementary material for: GrapeTree: visualization of core genomic relationships among 100,000 bacterial pathogens
Source: Genome Res. 2018 Sep;28(9):1395–404. doi: 10.1101/gr.232397.117 (PMC6120633; doi:10.1101/gr.232397.117)
Supplement: Supplemental Material [file supp_gr.232397.117_Supplemental_data_S3.zip › Supplemental_data/GrapeTree-codes/static/js/SlickGrid/examples/example10-async-post-render.html]

SlickGrid example 10: Async post render


Scores:

## Demonstrates:

With SlickGrid, you can still have rich, complex cells rendered against the actual DOM nodes while still preserving
the speed and responsiveness.
This is achieved through async background post-rendering.
SlickGrid exposes a asyncPostRender property on a column which you can use to set your own function that will
manipulate the cell DOM node directly.
The event is fired one by one for all visible rows in the viewport on a timer so it doesn't impact the UI
responsiveness.
You should still make sure that post-processing one row doesn't take too long though.
SlickGrid will figure out what and when needs to be updated for you.

The example below is a list of 500 rows with a title and 5 integer cells followed by graphical representation of
these integers.
The graph is drawn using a CANVAS element in the background.
The grid is editable, so you can edit the numbers and see the changes reflected (almost) immediately in the graph.
The graph cell behaves just like an ordinary cell and can be resized/reordered.
The graphs themselves are created using the excellent jQuery Sparklines library.  
  
nb: sparkline 2.x will cause a memory leak in the current architecture, so we use sparkline 1.x and jquery-migrate for now.

## View Source:

- View the source for this example on Github
