# Supplementary material for: GrapeTree: visualization of core genomic relationships among 100,000 bacterial pathogens
Source: Genome Res. 2018 Sep;28(9):1395–404. doi: 10.1101/gr.232397.117 (PMC6120633; doi:10.1101/gr.232397.117)
Supplement: Supplemental Material [file supp_gr.232397.117_Supplemental_data_S3.zip › Supplemental_data/GrapeTree-codes/static/js/SlickGrid/examples/example14-highlighting.html]

SlickGrid example 14: Highlighting and Flashing cells


## About

This example simulates a real-time display of CPU utilization in a web farm.
Data is updated in real-time, and cells with changed data are highlighted.
You can also click "Find current server" to scroll the row displaying data for the current
server into view and flash it.

## Demonstrates

- setHighlightedCells()
- flashCell()

## Controls

Start simulation
Find current server

## View Source:

- View the source for this example on Github
