# Supplementary material for: GrapeTree: visualization of core genomic relationships among 100,000 bacterial pathogens
Source: Genome Res. 2018 Sep;28(9):1395–404. doi: 10.1101/gr.232397.117 (PMC6120633; doi:10.1101/gr.232397.117)
Supplement: Supplemental Material [file supp_gr.232397.117_Supplemental_data_S3.zip › Supplemental_data/GrapeTree-codes/static/js/SlickGrid/examples/example2-formatters-event.html]

SlickGrid example 2a: Formatters with onBeforeAppendCell event


|  |  |
| --- | --- |
|  | Demonstrates:  - width, minWidth, maxWidth, resizable, cssClass column attributes - custom column formatters - use of onBeforeAppendCell to format cell classes (deprecated in preference of the extended formatter)  View Source:  - View the source for this example on Github |
