# Supplementary material for: GrapeTree: visualization of core genomic relationships among 100,000 bacterial pathogens
Source: Genome Res. 2018 Sep;28(9):1395–404. doi: 10.1101/gr.232397.117 (PMC6120633; doi:10.1101/gr.232397.117)
Supplement: Supplemental Material [file supp_gr.232397.117_Supplemental_data_S3.zip › Supplemental_data/GrapeTree-codes/static/js/SlickGrid/examples/example5-collapsing.html]

SlickGrid example 5: Collapsing


|  |  |
| --- | --- |
| Show tasks with % at least:     And title including: | Demonstrates:  - implementing expand/collapse as a filter for DataView  View Source:  - View the source for this example on Github |
