# Supplementary material for: GrapeTree: visualization of core genomic relationships among 100,000 bacterial pathogens
Source: Genome Res. 2018 Sep;28(9):1395–404. doi: 10.1101/gr.232397.117 (PMC6120633; doi:10.1101/gr.232397.117)
Supplement: Supplemental Material [file supp_gr.232397.117_Supplemental_data_S3.zip › Supplemental_data/GrapeTree-codes/static/js/SlickGrid/examples/example6-ajax-loading-yahoo.html]

SlickGrid example 6: AJAX Load


Yahoo Latest News stories

## Demonstrates:

- loading data through AJAX
- custom row height

## WARNING:

- Yahoo API access can be unreliable. Please be patient.
- The Yahoo API is primitive and does not correctly support keyword search. Ordering has not been implemented.

## View Source:

- View the source for this example on Github
