# Supplementary material for: GrapeTree: visualization of core genomic relationships among 100,000 bacterial pathogens
Source: Genome Res. 2018 Sep;28(9):1395–404. doi: 10.1101/gr.232397.117 (PMC6120633; doi:10.1101/gr.232397.117)
Supplement: Supplemental Material [file supp_gr.232397.117_Supplemental_data_S3.zip › Supplemental_data/GrapeTree-codes/static/js/SlickGrid/examples/example6-ajax-loading.html]

SlickGrid example 6: AJAX Load


Octopart Catalog Search

Search:

## Demonstrates:

- loading data through AJAX

## WARNING:

- API access through ThriftDB has some latency when paging through results. Be patient.

## View Source:

- View the source for this example on Github
