# Supplementary material for: GrapeTree: visualization of core genomic relationships among 100,000 bacterial pathogens
Source: Genome Res. 2018 Sep;28(9):1395–404. doi: 10.1101/gr.232397.117 (PMC6120633; doi:10.1101/gr.232397.117)
Supplement: Supplemental Material [file supp_gr.232397.117_Supplemental_data_S3.zip › Supplemental_data/GrapeTree-codes/static/js/SlickGrid/examples/example7-events.html]

SlickGrid example 7: Events


|  |  |
| --- | --- |
|  | Demonstrates:  - handling events from the grid: - Right-click the row to open the context menu - Click the priority cell to toggle values  View Source:  - View the source for this example on Github |

**Set priority:**- Low
- Medium
- High
