# Supplementary material for: GrapeTree: visualization of core genomic relationships among 100,000 bacterial pathogens
Source: Genome Res. 2018 Sep;28(9):1395–404. doi: 10.1101/gr.232397.117 (PMC6120633; doi:10.1101/gr.232397.117)
Supplement: Supplemental Material [file supp_gr.232397.117_Supplemental_data_S3.zip › Supplemental_data/GrapeTree-codes/static/js/SlickGrid/examples/example9-row-reordering-simple.html]

SlickGrid example 9: Row reordering


Santa's TODO list:

**Tips:**


---

Click to select, Ctrl-click to toggle selection, Shift-click to select a range.  
Drag one or more rows by the handle to reorder.
  
  

## View Source:

- View the source for this example on Github
