# Supplementary material for: GrapeTree: visualization of core genomic relationships among 100,000 bacterial pathogens
Source: Genome Res. 2018 Sep;28(9):1395–404. doi: 10.1101/gr.232397.117 (PMC6120633; doi:10.1101/gr.232397.117)
Supplement: Supplemental Material [file supp_gr.232397.117_Supplemental_data_S3.zip › Supplemental_data/GrapeTree-codes/static/js/SlickGrid/examples/index.html]

SlickGrid Examples


# Examples

This repo has significantly expanded the set of Examples from the mleibman repo

## Basic Use

- Basic use with minimal configuration
- Adding some formatting
- Handling events and context menu
- Highlighting and flashing cells

## Editing

- Making it editable
- Writing compound editors
- Implementing Undo
- Using a CompositeEditor to implement detached item edit form

## Layout

- Using pre-compiled micro-templates to render cells
- Spreadsheet: cell range selection, copy’n’paste and Excel-style formula editor
- No vertical scrolling
- Filling the whole window
- Colspan

## Data-Centric

- Implementing a totals row via a data provider
- (most comprehensive) Using a filtered data view to drive the grid
- Optimizing DataView for 500’000 rows
- AJAX-loading data with search
- AJAX-loading data, second example
- Sorting by an index, getItem method
- Using fixed header row for quick filters
- Multi-column sorting
- Using dataItemColumnValueExtractor option to specify a custom column value extractor

## Grouping

- Interactive grouping and aggregates.
- Adding tree functionality (expand/collapse) to the grid

## Other Features

- Explicit initialization
- Row selection & reordering
- Using background post-rendering to add graphs
- Background post-rendering with async cleanup

## Bootstrap, Dynamic Grids and Third Party component editors

- Putting multiple grids on a page
- With Bootstrap 3 header
- SlickGrid inside jQuery Accordion
- Dynamically created SlickGrid inside jQuery tabs
- Dynamically created SlickGrid with DataView and filtering, inside jQuery tabs
- jQuery Autocomplete editor
- Select2 javascript drop-down editor

## Plugins

- Plugin: Checkbox row selectors with CheckboxSelectColumn plugin
- Plugin: Column header buttons
- Plugin: Column header menu
- Plugin: Auto tooltips
