# Supplementary material for: GrapeTree: visualization of core genomic relationships among 100,000 bacterial pathogens
Source: Genome Res. 2018 Sep;28(9):1395–404. doi: 10.1101/gr.232397.117 (PMC6120633; doi:10.1101/gr.232397.117)

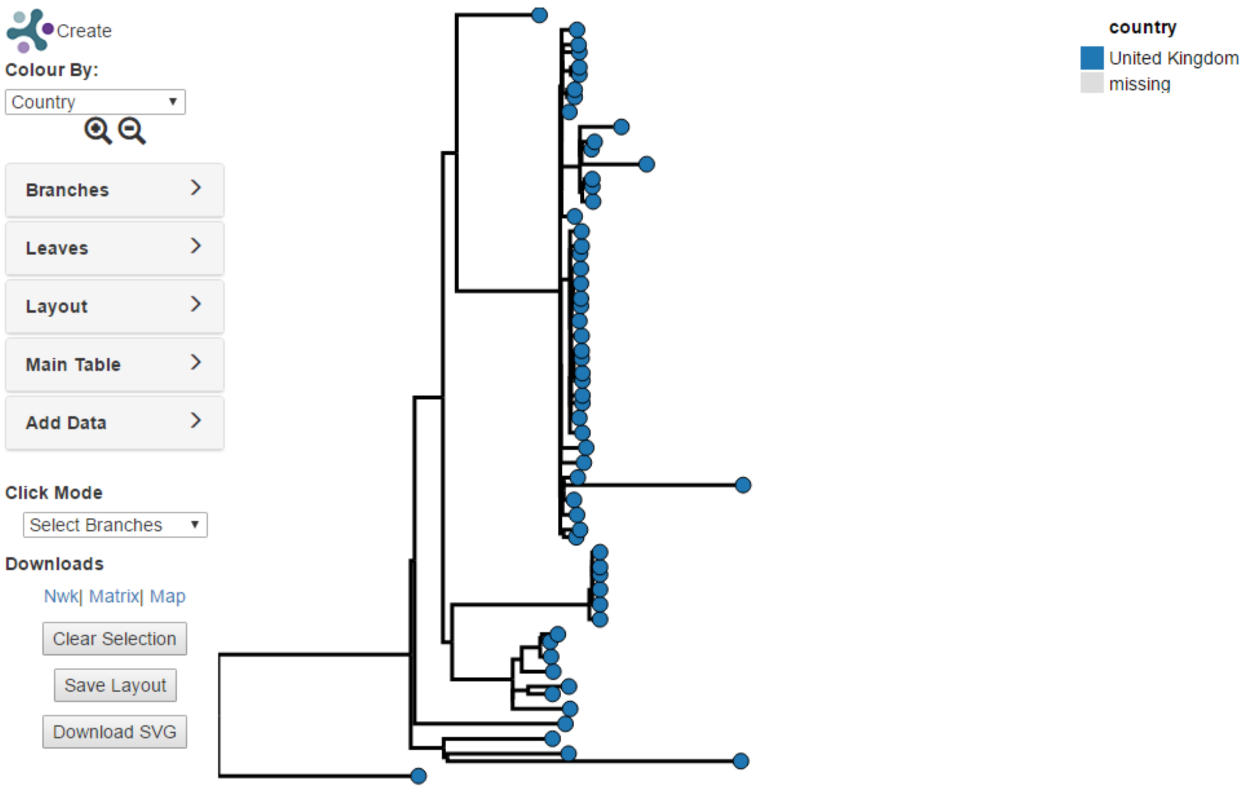

1.7 GrapeTree Documentation

GrapeTree is a fully interactive, tree visualization program within EnteroBase, which supports facile manipulations of both tree layout and metadata. It generates GrapeTree figures using the neighbor-joining (NJ) algorithm, the classical minimal spanning tree algorithm (MSTree) similar to PhyloViz, or an improved minimal spanning tree algorithm which we call MSTree V2.

- GrapeTree is also available as a stand-alone version.
- *Installation instructions*, *Manuals* and *Tutorials* are available on this site
- The source code for GrapeTree is available
- GrapeTree is also available as a live online demo

Here are materials to help you use GrapeTree:

1.7.1 Installing stand-alone GrapeTree

The stand-alone version emulates the EnteroBase version through a lightweight webserver running on your local computer. You will be interacting with the program as you would in EnteroBase; through a web browser. We recommend Google Chrome for best results. There are number of different ways to interact with GrapeTree, the easiest is to install via pip, or download the ready-built software here: https://github.com/achtman-lab/GrapeTree/releases

Installing and Running GrapeTree

Install via pip

```
pip install grapetree
grapetree
```

For more information see: https://pypi.org/project/GrapeTree/

Running on Mac: Download GrapeTree_mac.zip

You will need to unzip GrapeTree_mac.zip (just double click). Inside there will be a directory. From this directory launch grapetree_mac. You may be warned about Security settings, if you right click on the grapetree_mac and then click "Open" it should be fine.

Running on Windows: Download GrapeTree_win.zip

Once downloaded, you will need to unzip GrapeTree_win.zip and then open the extracted folder and run GrapeTree_win.exe. When you run it the first time on windows you might get a prompt about security. On Windows 10, click the small text: "More info", and then the button "Run Anyway".

Running from Source code

EnteroMSTree - GrapeTree requires Python 2.7 and some additional python modules (listed in requirements.txt). The easiest way to install these modules is with pip:

```
pip install -r requirements.txt
chmod +x binaries/
```

On Linux or MacOSX you need to make sure the binaries in binaries/ can be executed. To run GrapeTree;

1. Navigate to the directory where you installed GrapeTree.
2. Run it through python as below.

```
\GrapeTree>python main.py
 * Running on http://127.0.0.1:8000/ (Press CTRL+C to quit)
```

Running GrapeTree with no installation

If you just want to view and manipulate trees, without processing profile/FASTA files, it can simply be run by opening *MSTree_holder.html* in your web browser. It requires the 'static folder'. This is essentially what happens if you visit https://achtman-lab.github.io/GrapeTree/MSTree_holder.html

First look at GrapeTree

The program will automatically open your web browser and you will see the GrapeTree Splash Screen, as shown below.

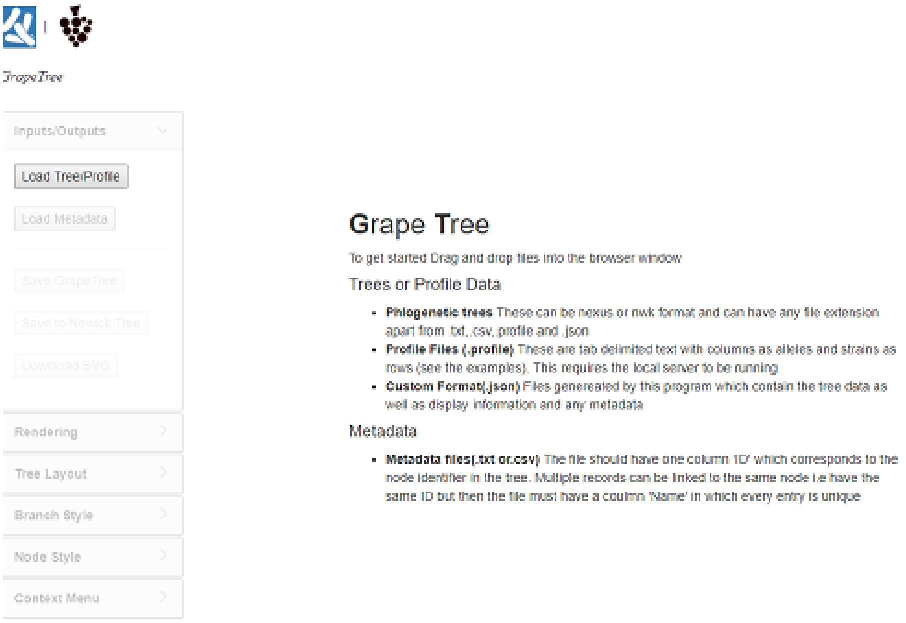

If at anytime you want to restart the page you can visit http://localhost:8000 in your web browser, as shown below.

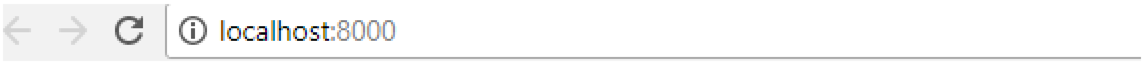

Let's apply these concepts with data from a previously published study of *Salmonella enterica* serovar Agona. *Click here for the first tutorial*

1.7.2 Tutorial 1A: Basic Usage of GrapeTree (EnteroBase version)

The procedure for working with GrapeTree in EnteroBase and stand-alone are identical, once you have loaded your data. This tutorial assumes you are using EnteroBase. There are other tutorials specifically for the Stand-alone version.

You will need to be a registered user of EnteroBase, see *Getting Started - Registering and logging in*.

About this dataset

To learn the basic usage of GrapeTree we will be using data presented in Zhou et al. "Neutral genomic microevolution of a recently emerged pathogen, Salmonella enterica serovar Agona". PLoS genetics 9.4 (2013): e1003471. We will try to replicate the tree presented in Figure 1, which shows phylogeny of serovar Agona including a number of outbreaks (green diamonds) across the world.

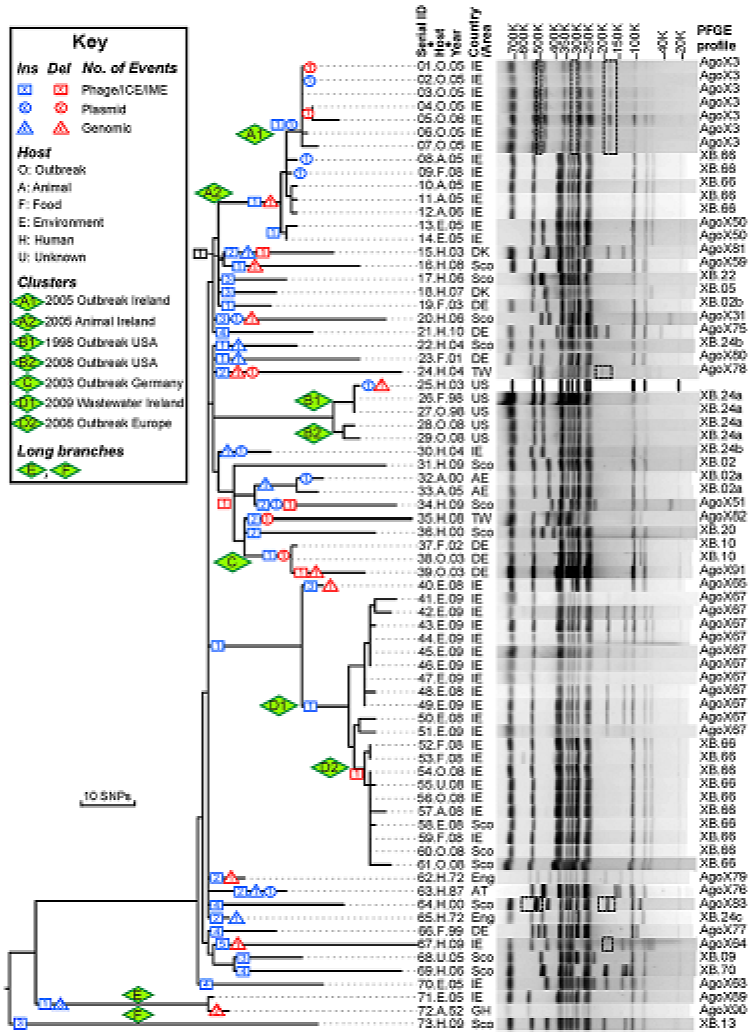

1. Searching EnteroBase

This tutorial assumes you are already familiar with finding records in EnteroBase. If you are not, please read *Searching EnteroBase*. In the case of this example, the genomes are listed under Bio Project ID PRJEB1944.

1. Perform the search in EnteroBase. This will load 71 records.

    (a) Show sub strains box should be checked

    (b) Search terms should be Bio Project ID (Field) contains (Operator) PRJEB1944 (Value)

1. From the Experimental Data dropdown on the right select cgMLST
2. Click the Create MSTree button, once the cgMLST data has loaded.
3. Leave the Algorithm option as MSTreeV2 and give the tree a title and click Submit.

Make sure your browser allows pop-ups for EnteroBase! You should now have a new window open showing a tree similar to the one below.

Before we get to far ahead, there is some custom metadata that describes the outbreak clusters from the paper. To load it, Open the EnteroBase panel:

1. Click import fields

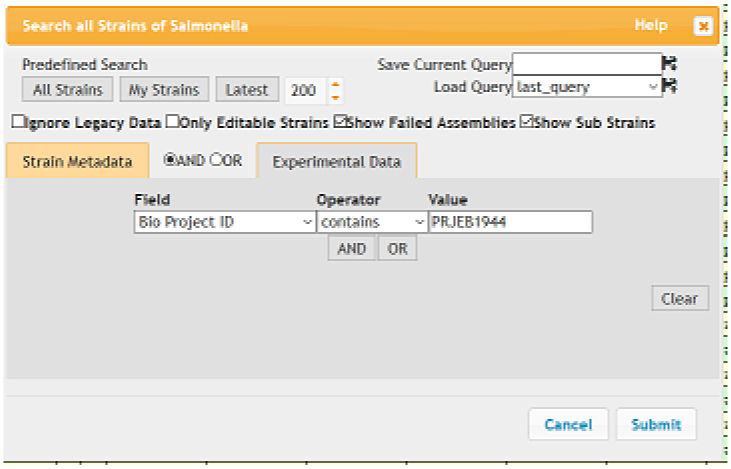
Search all Strains of Salmonella
Help
Predefined Search
All Strains
My Strains
Latest
200
Save Current Query
Load Query
last_query
Ignore Legacy Data
Only Editable Strains
Show Failed Assemblies
Show Sub Strains
Strain Metadata
AND
OR
Experimental Data
Field
Operator
Value
Bio Project ID
contains
PRJEB1944
AND
OR
Clear
Cancel
Submit

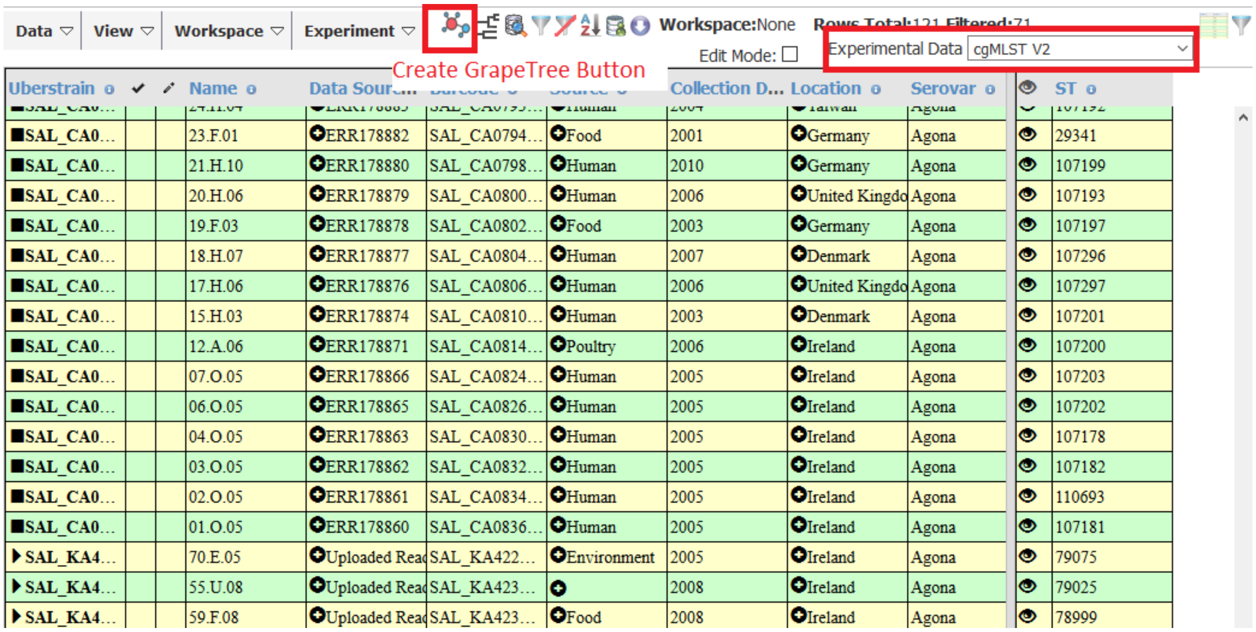
Data
View
Workspace
Experiment
Workspace:None
Edit Mode:
Experimental Data
cgMLST V2
Create GrapeTree Button
Uberstrain
Name
Collection D...
Location
Serovar
ST
SAL_CA0... 23.F.01 ERR178882 SAL_CA0794... Food 2001 Germany Agona 29341
SAL_CA0... 21.H.10 ERR178880 SAL_CA0798... Human 2010 Germany Agona 107199
SAL_CA0... 20.H.06 ERR178879 SAL_CA0800... Human 2006 United Kingdo Agona 107193
SAL_CA0... 19.F.03 ERR178878 SAL_CA0802... Food 2003 Germany Agona 107197
SAL_CA0... 18.H.07 ERR178877 SAL_CA0804... Human 2007 Denmark Agona 107296
SAL_CA0... 17.H.06 ERR178876 SAL_CA0806... Human 2006 United Kingdo Agona 107297
SAL_CA0... 15.H.03 ERR178874 SAL_CA0810... Human 2003 Denmark Agona 107201
SAL_CA0... 12.A.06 ERR178871 SAL_CA0814... Poultry 2006 Ireland Agona 107200
SAL_CA0... 07.O.05 ERR178866 SAL_CA0824... Human 2005 Ireland Agona 107203
SAL_CA0... 06.O.05 ERR178865 SAL_CA0826... Human 2005 Ireland Agona 107202
SAL_CA0... 04.O.05 ERR178863 SAL_CA0830... Human 2005 Ireland Agona 107178
SAL_CA0... 03.O.05 ERR178862 SAL_CA0832... Human 2005 Ireland Agona 107182
SAL_CA0... 02.O.05 ERR178861 SAL_CA0834... Human 2005 Ireland Agona 110693
SAL_CA0... 01.O.05 ERR178860 SAL_CA0836... Human 2005 Ireland Agona 107181
SAL_KA4... 70.E.05 Uploaded Read SAL_KA422... Environment 2005 Ireland Agona 79075
SAL_KA4... 55.U.08 Uploaded Read SAL_KA423... 2008 Ireland Agona 79025
SAL_KA4... 59.F.08 Uploaded Read SAL_KA423... Food 2008 Ireland Agona 78999

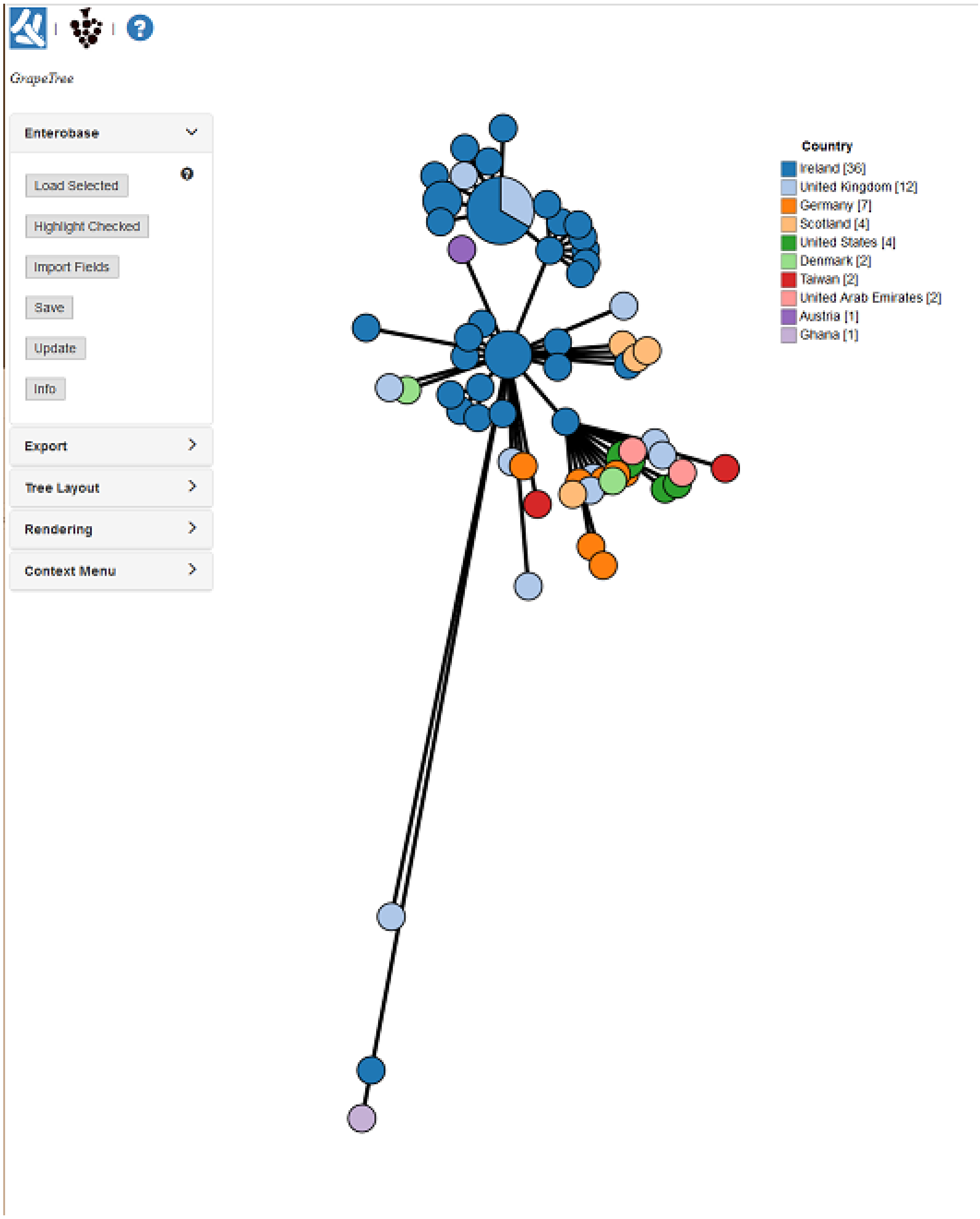
GrapeTree
Enterobase
Load Selected
Highlight Checked
Import Fields
Save
Update
Info
Export
Tree Layout
Rendering
Context Menu
Country
Ireland [36]
United Kingdom [12]
Germany [7]
Scotland [4]
United States [4]
Denmark [2]
Taiwan [2]
United Arab Emirates [2]
Austria [1]
Ghana [1]

2. Under "add custom column" select 'agona_cluster' and click add
3. agona_cluster should show up in the column list on the right. Click OK

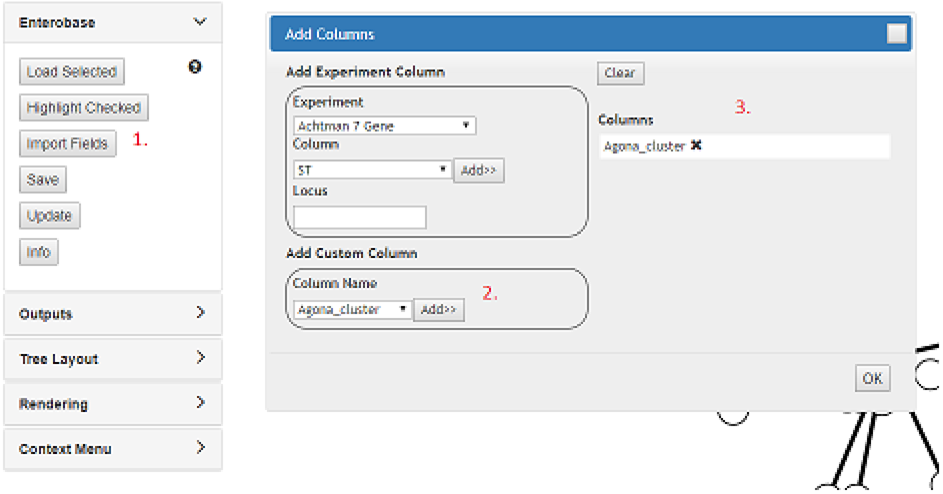

2. Basic Orientation

Let's stop and orientate ourselves with the GrapeTree interface:

1. These are links to important webpages (Top left). The Left icon opens a new browser page to EnteroBase, while the right opens the GrapeTree GitHub page.
2. The text here (Top left) shows may show the filename of the file we just loaded in.
3. This set of panels (Left) contains all the options for customizing our tree. Currently the Input/output panel is open and gives options to load trees/profiles, load metadata, and options to save our work.
4. The GrapeTree Tree itself (Centre), the figure is interactive. Each circle is a Node and each line is a branch. Node size is dependent on the number of strains within that node. Branch length varies on the distance between nodes.
5. The Key/Legend (Right) for the colour coding. You can change some settings by right clicking on it.

5. Basic Navigation

GrapeTree has a rich suite of tools to help you navigate and manipulate your tree. Try these out!

1. **If you get lost** click *Centre Tree* under *Tree Layout*. Click *Tree Layout* to open or close the Layout panel.
2. **If you've messed up the tree** Click *Static Redraw* under *Tree Layout* to reset the layout.
3. **Zoom in/out** using the mousewheel or the Zoom buttons under *Tree Layout*. Click *Tree Layout* to open up the Layout panel and then click the magnifying glass (+) to zoom in or magnifying glass (-) to zoom out.
4. **Move the Tree** by click & hold on any of the whitespace around the tree, and then drag.
5. **Move a node and its children** by Click and holding the left mouse button down on a Node and then drag.
6. **Move the key/legend** by Click and holding the legend and then drag to move it around.
7. **Rotate the entire tree** by Click and holding the root node and then drag.
8. **Select some nodes** by holding SHIFT key and dragging over some nodes in the tree. You can also select individual nodes by holding the SHIFT key and clicking on nodes one-by-one.

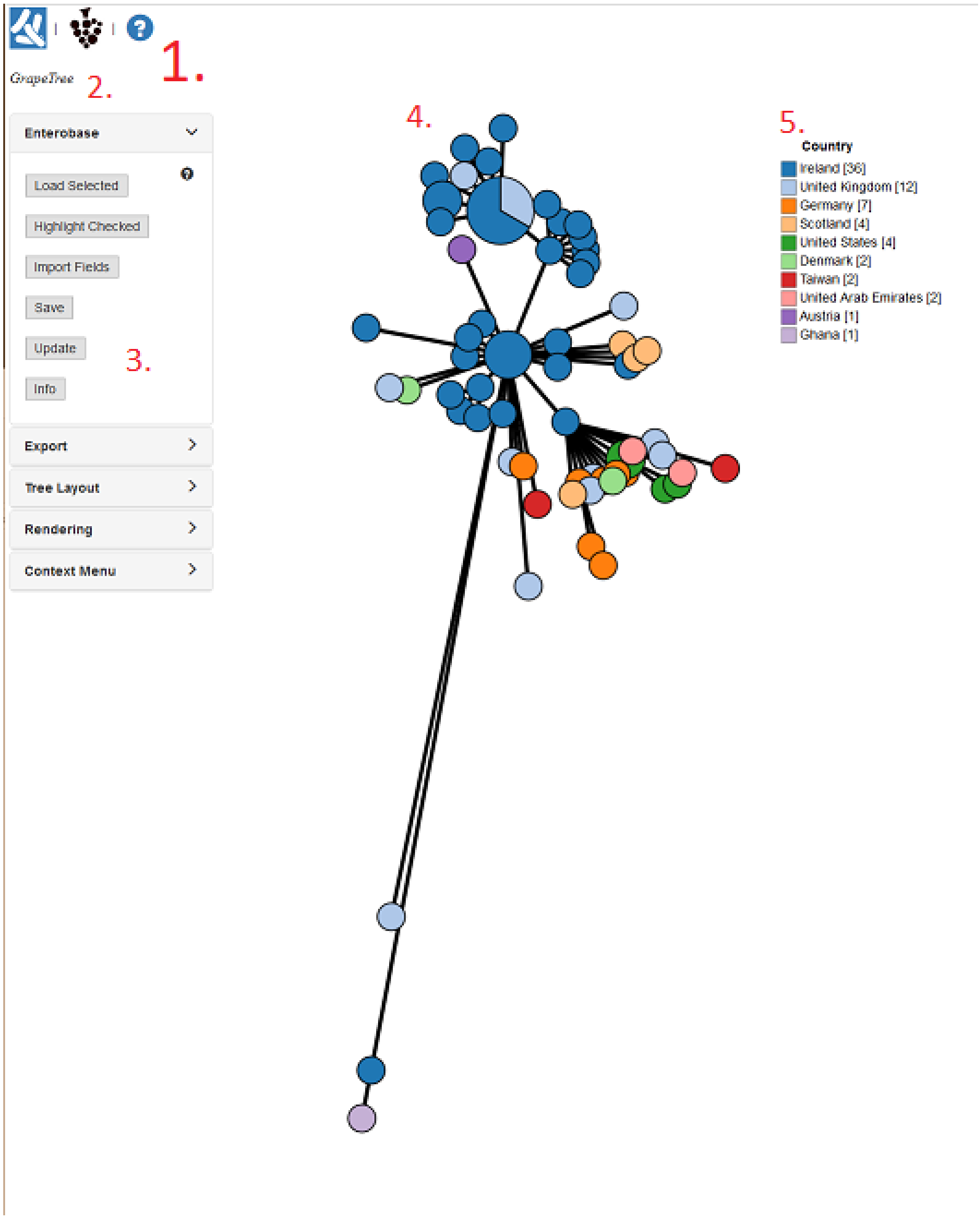
GrapeTree
1.
2.
Enterobase
Load Selected
Highlight Checked
Import Fields
Save
Update
Info
3.
Export
Tree Layout
Rendering
Context Menu
4.
5.
Country
Ireland [36]
United Kingdom [12]
Germany [7]
Scotland [4]
United States [4]
Denmark [2]
Taiwan [2]
United Arab Emirates [2]
Austria [1]
Ghana [1]

9. **Add more nodes to your selection** by holding SHIFT key and dragging over other nodes in the tree.
10. **Deselect some nodes** by holding SHIFT key and dragging over some already selected nodes. Try removing nodes from your current selection.
11. **Deselect all selected Nodes** by double-clicking any whitespace around the tree, or by right-clicking and choosing *Unselect all* from the contextual menu.

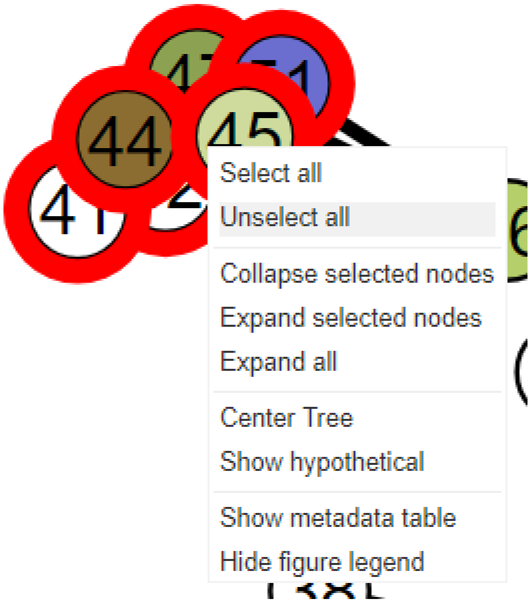

This is enough to get started, let's tidy up our tree.

6. Modifying the Tree Layout

The Tree Layout panel allows global changes to tree layout, nodes and branches and has some important navigation features. Try playing around with each of the settings to see what they do. Generally:

- You can drag the sliders to change the value.
- You can also directly modify the value by clicking on the value box, typing in a new value, and pressing enter or clicking out of the box.
- Click the refresh icon (the rewind icon) to reset the value to default.

Specifically under *Tree Layout* > *Branch Style*:

- **Scaling:** will uniformly increase the scaling for all branches. For instance, setting it to 200% will double the length of all branches relative to the default setting (100%); whereas 50% would halve it.
- **Collapse Branches:** will collapse all branches under a certain length. The length value shown is the real branch length for the tree. To see the lengths for all branches, check the *Branch Labels* under *Branch Style*. The slider is scaled relatively, so moving it all the way to the right will collapse all the nodes giving you a pie graph.
- **Log Scale:** if this is checked (has a tick in the box), all branches will be Log-scaled. This is useful for trees with a wide variety of branch lengths.

Specifically under *Tree Layout* > *Node Style*:

- **Node Size:** will uniformly increase the scaling for all Nodes. For instance, setting it to 200% will double the size of all Nodes relative to the default setting (100%); whereas 50% would halve it.
- **Node Scaling:** This will exaggerate differences in node size. In the case of the of the Agona dataset, all nodes include only one strain so there will be no effect.

For this tutorial set the Branch length to 150% (as shown below) and we shall continue.

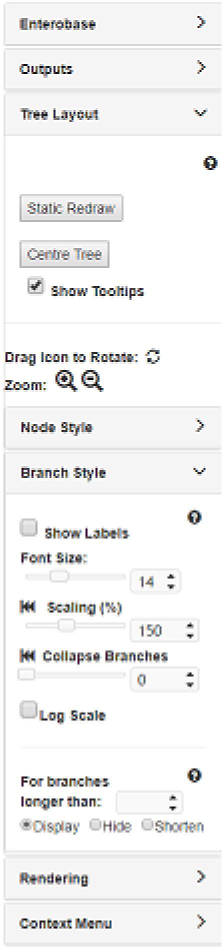

7. Styling the Branches

Under *Branch Style* we can also modify the look of the Branches in our Tree. We can show branch labels by checking the *Branch Labels* option and change the font size with the *Font Size* slider or by entering a new value in the box. If the *Mouseover info* box is checked we can see the branch length when we have the mouse cursor over a particular branch.

The tree of the Agona dataset has very long branches. Enter 100 in the box next to *For branches longer than* and set it to ‘Shorten’. This will shorten the branch length and change the line to be dashed, which indicates the branch length is not to scale.

8. Node settings

Under *Tree Layout* > *Node Style* we can modify the look of the Nodes in our Tree. We can show nodes labels by checking the *Node Labels* option and change the font size with the *Font Size* slider or by entering a new value in the box. If the *Mouseover info* box is checked we can see details for that node when we mouse over.

We can also set the colour coding of the Nodes. For the Agona dataset, Set *Colour by* to "Agona_Clusters" to show the outbreaks as defined the original paper. See if you can your figure to look like mine.

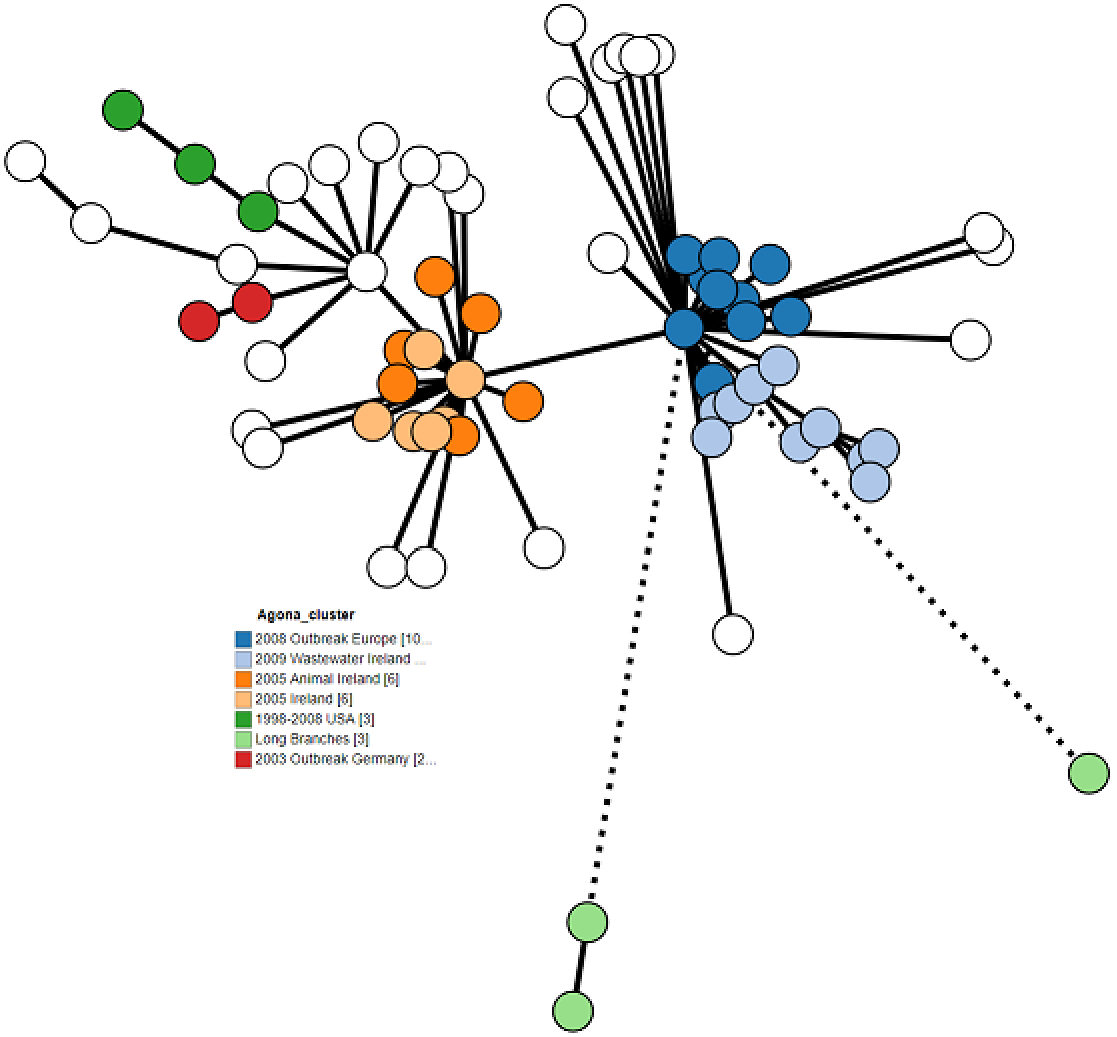

9. Final modifications

The tree is looking pretty good, but we can make it a bit clearer. Try playing around with all of the different options to come up with the best looking tree. Here's what I came up with:

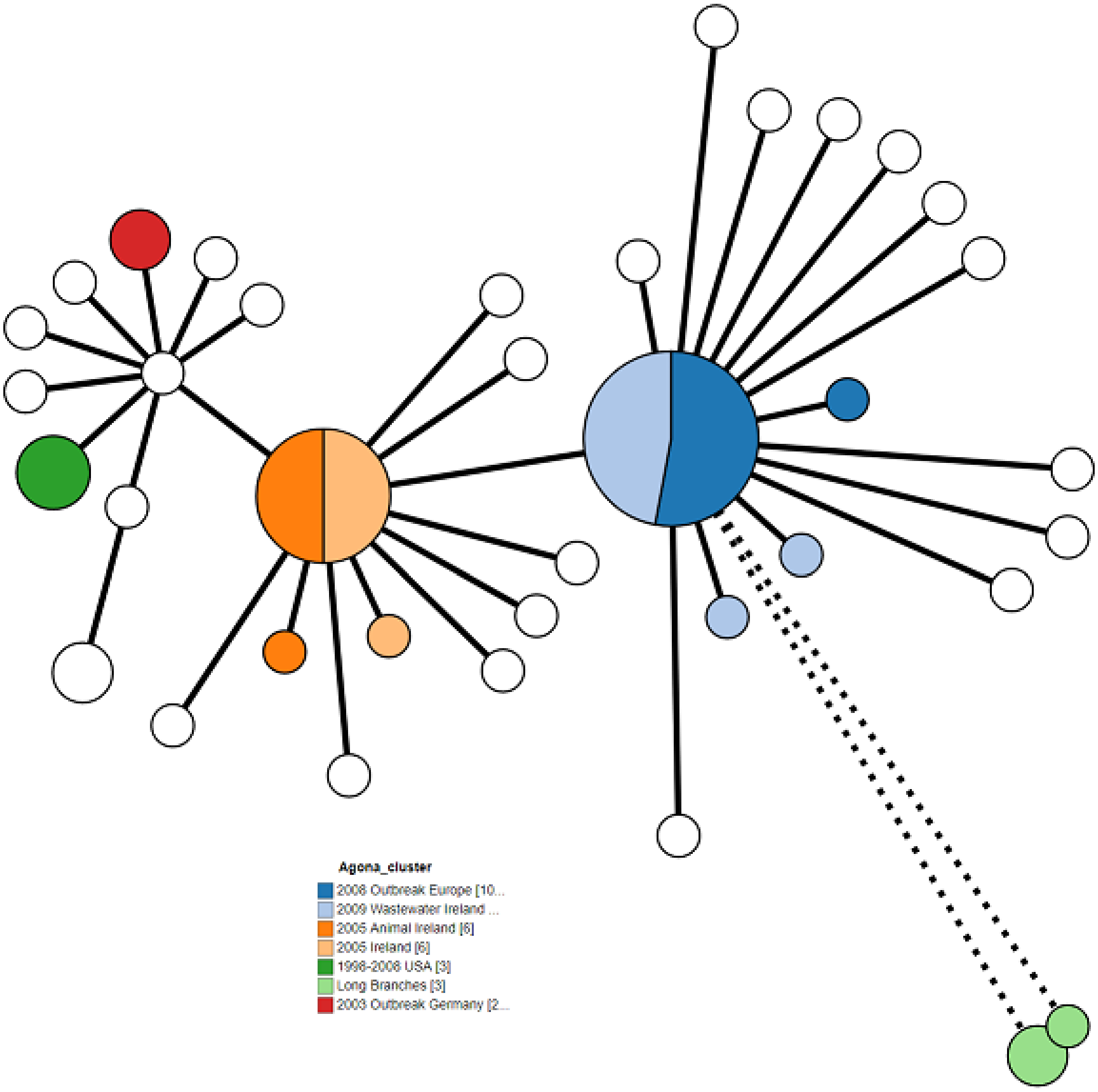

These are my settings:

- **Tree Layout > Branch Length**: 150%
- **Tree Layout > Collapse Branches**: 10
- **Branch Style > Shorten branches longer than**: 100
- **Node Style > Colour by:** Clusters
- **Node Style > Node Labels:** Unchecked/Off

10. Exporting our work

Your tree can be save in either GrapeTree's JSON format, as a Newick tree that can be loaded into other phylogeny programs and as a Scalar Vector Graphic (SVG), which is an image format that you can edit in publishing software like

Inkscape or Adobe Illustrator. If you would like a raster image (JPEG, PNG, BMP) of the Tree, just use the screenshot feature of your computer.

1.7.3 Tutorial 1B: Basic Usage of GrapeTree (Stand-alone)

The procedure for working with GrapeTree in EnteroBase and stand-alone are identical, once you have loaded your data. This tutorial assumes you are using the stand-alone version. There are other tutorials specifically for the EnteroBase version.

You will need to install GrapeTree, *Installing stand-alone GrapeTree*.

About this dataset

To learn the basic usage of GrapeTree we will be using data presented in Zhou et al. "Neutral genomic microevolution of a recently emerged pathogen, Salmonella enterica serovar Agona". PLoS genetics 9.4 (2013): e1003471. We will try to replicate the tree presented in Figure 1, which shows phylogeny of serovar Agona including a number of outbreaks (green diamonds) across the world.

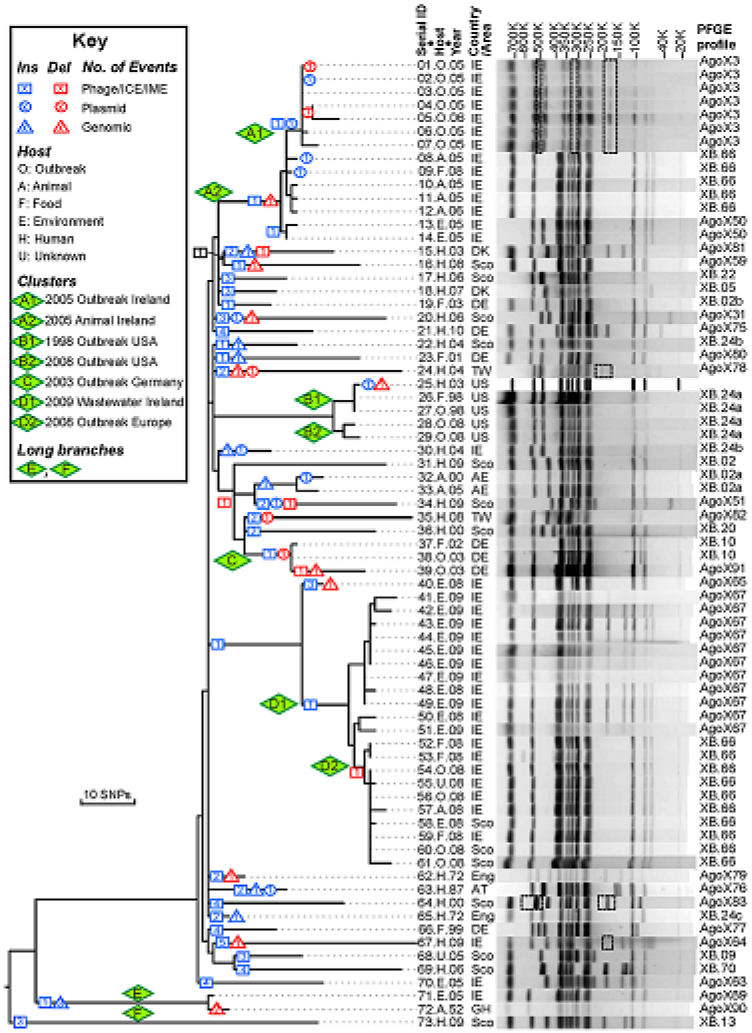

1. Loading data into GrapeTree with the stand-alone version

Download the sample data as shown below.

https://raw.githubusercontent.com/martinSergeant/EnteroMSTree/master/examples/Grapetree_Agona.profile

https://raw.githubusercontent.com/martinSergeant/EnteroMSTree/master/examples/Grapetree.Agona.meta.tsv

The profile file includes *Salmonella* cgMLST data from EnteroBase for the strains described in the paper. The first row contains the headers for each column "Name", "ST" (Sequence Type), and each subsequent column are the names of locus within the cgMLST scheme.

If you wish to use your own profile:

- Profiles must be tab or comma delimited.
- You MUST include a "#" symbol as the start of the row. Note in the example below that "Name" is in fact "#Name".
- You may use a SNP matrix, which is the same format as the Agona example, with single nucleotides (A,T,G or C) substituting for the numbers.

	A	B	C	D	E	F	G	H	I	J	K	L	M	N	O
1	#Name	ST	STMMW_08711	STMMW_08241	STMMW_23011	STMMW_07941	STMMW_	STMMW_	STMMW_	STMMW_	STMMW_	STMMW_	STMMW_(	STMMW_	STMMW_
2	1	29306	34	34	43	35	35	39	29	33	18	15	25	37	2
3	3	29307	34	34	43	35	35	39	29	33	18	15	25	37	2
4	4	29308	34	34	43	35	35	39	29	33	18	15	25	37	2
5	5	29310	34	34	43	35	35	39	29	33	18	15	25	37	2
6	6	29312	34	34	43	35	35	39	29	33	18	15	25	37	2
7	7	29314	34	34	43	35	35	39	29	33	18	15	25	37	2
8	8	29316	34	34	43	35	35	39	29	692	18	15	25	37	2
9	9	29317	34	34	43	35	35	39	29	33	18	15	25	37	2
10	10	29319	34	34	43	35	35	39	29	33	18	15	25	37	2
11	11	29321	34	34	43	35	35	39	29	33	18	15	25	37	2
12	12	29323	34	34	43	35	35	39	29	33	18	15	25	37	2
13	14	29325	34	34	43	35	35	39	29	33	18	15	25	37	2
14	15	29327	34	34	43	35	35	39	29	33	18	15	25	37	2
15	16	29328	34	34	43	35	35	39	29	33	18	15	25	37	2
16	17	29330	34	34	43	35	35	39	29	33	18	15	25	37	2
17	18	29332	34	34	43	35	35	39	29	33	18	15	25	37	2
18	19	29333	34	34	43	35	35	39	29	33	18	15	25	37	2
19	20	29335	34	34	43	35	35	39	29	33	18	15	25	37	2
20	21	29337	34	34	43	35	35	39	29	33	18	15	25	37	2
21	22	29339	34	34	43	35	35	39	29	33	18	15	25	37	2
22	23	29341	34	34	43	35	35	39	29	33	18	15	25	37	2
23	24	29342	34	34	43	35	35	39	29	33	18	15	25	484	2
24	27	29346	34	34	43	35	35	39	29	33	18	15	25	37	2

The metadata file is slightly modified version of what is available in EnteroBase.

If you wish to use your own metadata:

- Metadata must be tab or comma delimited.
- One column must be labelled ID and these values should correspond to the names in the profile file.

2. Install and Start GrapeTree

Start GrapeTree as described here, *Installing stand-alone GrapeTree*.

3. Load in the Profile file and Metadata

You should now see the GrapeTree interface with the splash screen. You can either drag-and-drop the profile file into the window or click *Load Files* and navigate to the file through the file browser.

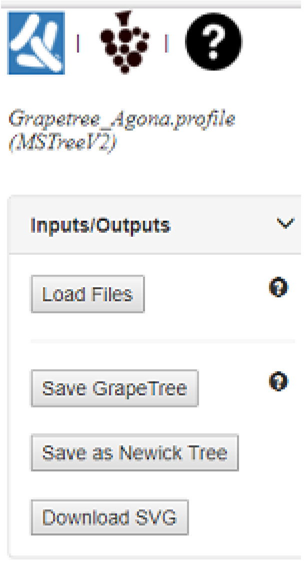

You will be prompted to select the *Parameters For Tree Creation*. The method should be set to MSTTreeV2 in the dropdown and then click OK.

Repeat the process with the metadata file. Either drag-and-drop the file into the window or click *Load Files* and navigate to the file. You should now see the tree colored with a metadata field as shown below.

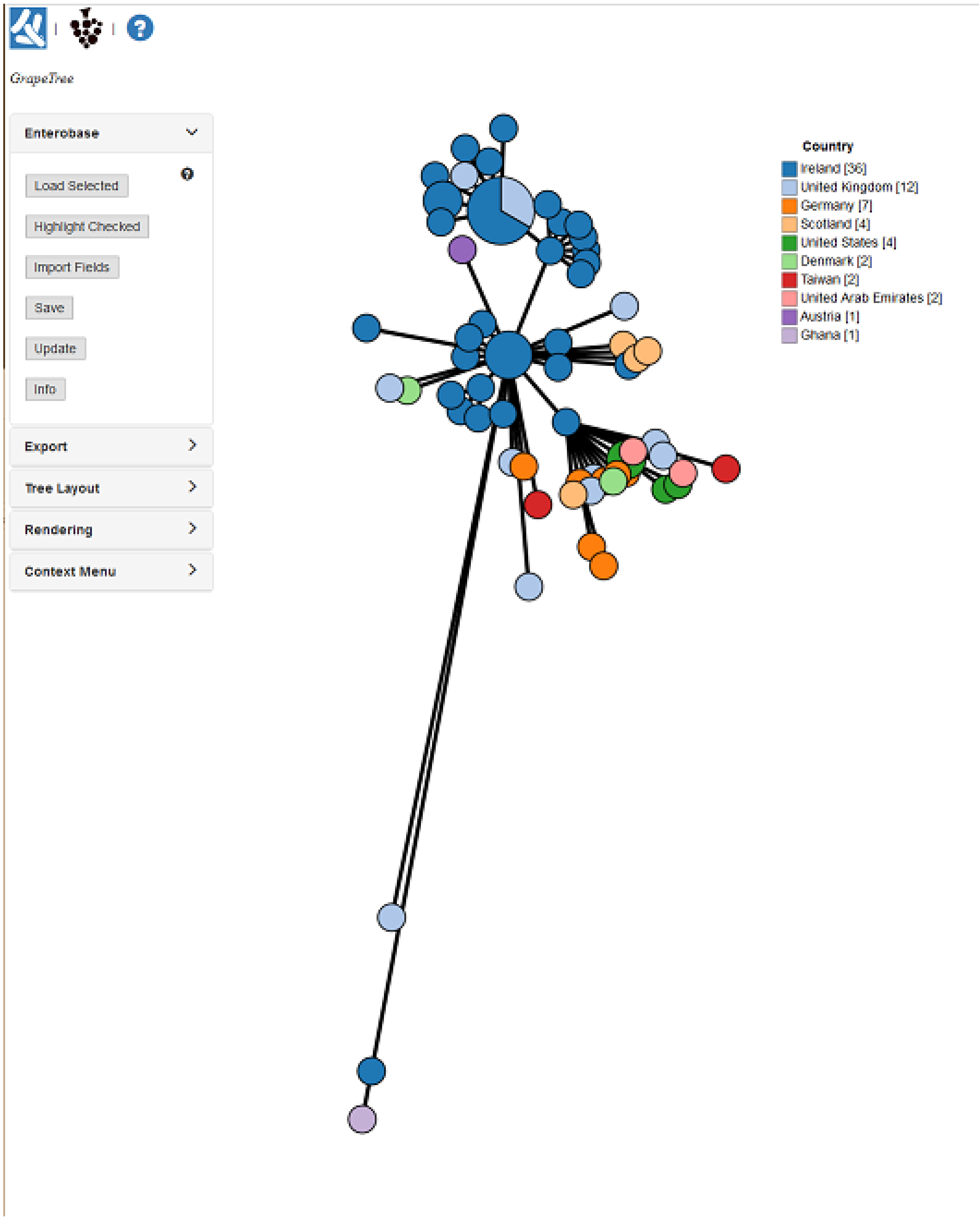

2. Basic Orientation

Let's stop and orientate ourselves with the GrapeTree interface:

1. These are links to important webpages (Top left). The Left icon opens a new browser page to EnteroBase, while the right opens the GrapeTree GitHub page.
2. The text here (Top left) shows may show the filename of the file we just loaded in.
3. This set of panels (Left) contains all the options for customizing our tree. Currently the Input/output panel is open and gives options to load trees/profiles, load metadata, and options to save our work.
4. The GrapeTree Tree itself (Centre), the figure is interactive. Each circle is a Node and each line is a branch.

Node size is dependent on the number of strains within that node. Branch length varies on the distance between nodes.

5. The Key/Legend (Right) for the colour coding. You can change some settings by right clicking on it.

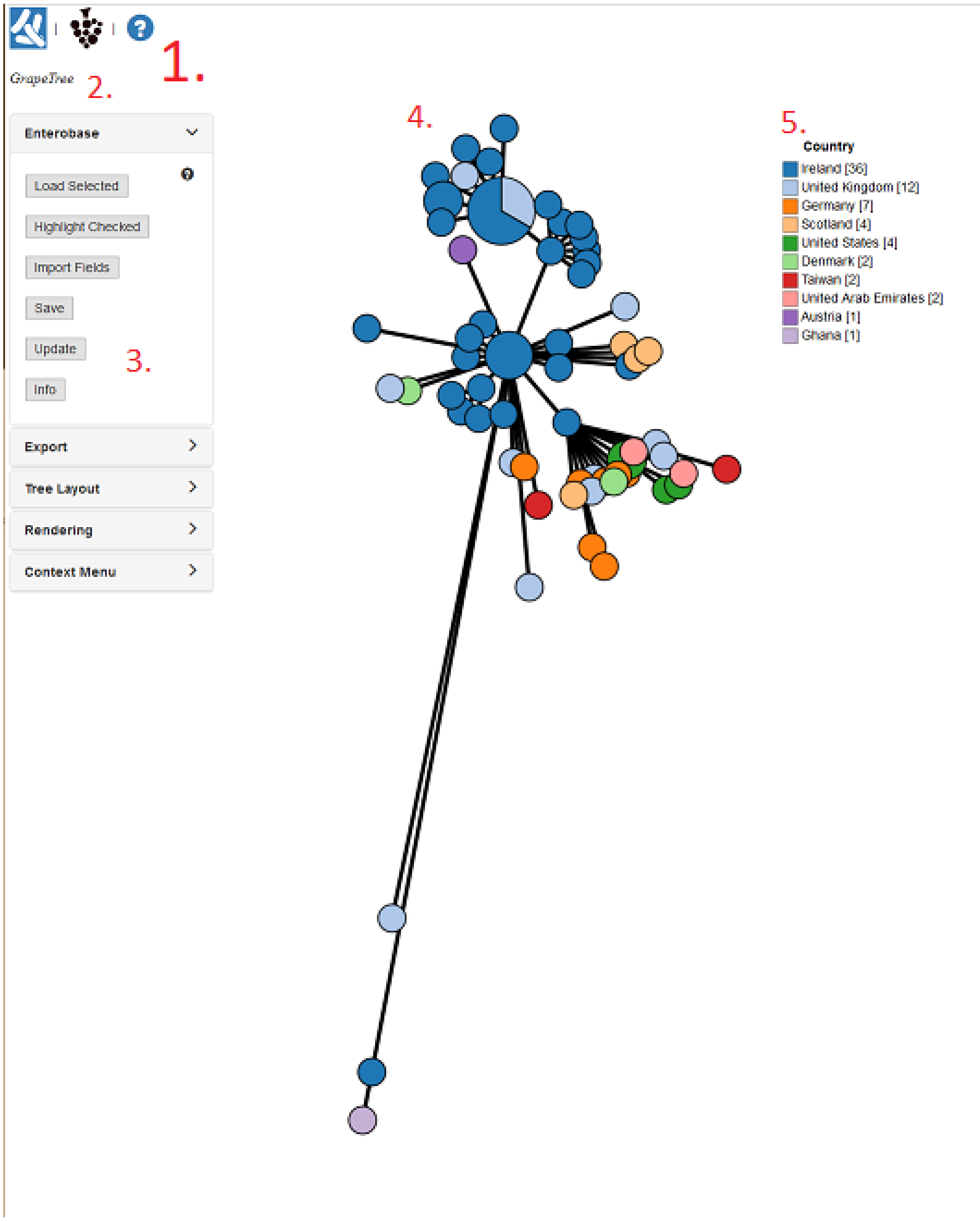

5. Basic Navigation

GrapeTree has a rich suite of tools to help you navigate and manipulate your tree. Try these out!

1. **If you get lost** click *Centre Tree* under *Tree Layout*. Click *Tree Layout* to open or close the Layout panel.
2. **If you've messed up the tree** Click *Static Redraw* under *Tree Layout* to reset the layout.

3. **Zoom in/out** using the mousewheel or the Zoom buttons under *Tree Layout*. Click *Tree Layout* to open up the Layout panel and then click the magnifying glass (+) to zoom in or magnifying glass (-) to zoom out.
4. **Move the Tree** by click & hold on any of the whitespace around the tree, and then drag.
5. **Move a node and its children** by Click and holding the left mouse button down on a Node and then drag.
6. **Move the key/legend** by Click and holding the legend and then drag to move it around.
7. **Rotate the entire tree** by Click and holding the root node and then drag.
8. **Select some nodes** by holding SHIFT key and dragging over some nodes in the tree. You can also select individual nodes by holding the SHIFT key and clicking on nodes one-by-one.
9. **Add more nodes to your selection** by holding SHIFT key and dragging over other nodes in the tree.
10. **Deselect some nodes** by holding SHIFT key and dragging over some already selected nodes. Try removing nodes from your current selection.
11. **Deselect all selected Nodes** by double-clicking any whitespace around the tree, or by right-clicking and choosing *Unselect all* from the contextual menu.

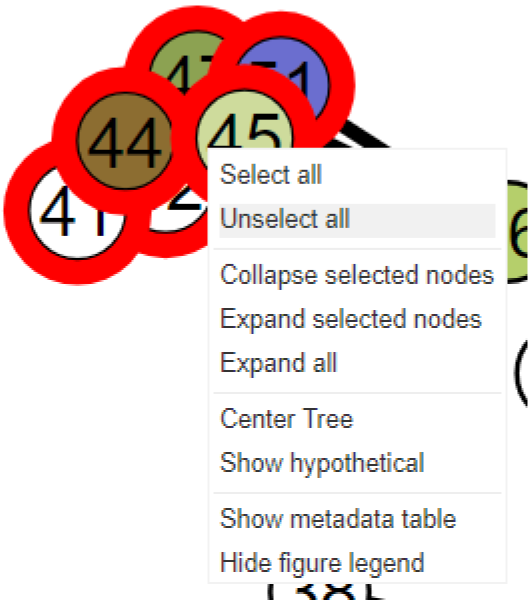

This is enough to get started, let's tidy up our tree.

6. Modifying the Tree Layout

The Tree Layout panel allows global changes to tree layout, nodes and branches and has some important navigation features. Try playing around with each of the settings to see what they do. Generally:

- You can drag the sliders to change the value.
- You can also directly modify the value by clicking on the value box, typing in a new value, and pressing enter or clicking out of the box.
- Click the refresh icon (the rewind icon) to reset the value to default.

Specifically under *Tree Layout* > *Branch Style*:

- **Scaling:** will uniformly increase the scaling for all branches. For instance, setting it to 200% will double the length of all branches relative to the default setting (100%); whereas 50% would halve it.
- **Collapse Branches:** will collapse all branches under a certain length. The length value shown is the real branch length for the tree. To see the lengths for all branches, check the *Branch Labels* under *Branch Style*. The slider is scaled relatively, so moving it all the way to the right will collapse all the nodes giving you a pie graph.
- **Log Scale:** if this is checked (has a tick in the box), all branches will be Log-scaled. This is useful for trees with a wide variety of branch lengths.

Specifically under *Tree Layout* > *Node Style*:

- **Node Size:** will uniformly increase the scaling for all Nodes. For instance, setting it to 200% will double the size of all Nodes relative to the default setting (100%); whereas 50% would halve it.
- **Node Scaling:** This will exaggerate differences in node size. In the case of the of the Agona dataset, all nodes include only one strain so there will be no effect.

For this tutorial set the Branch length to 150% (as shown below) and we shall continue.

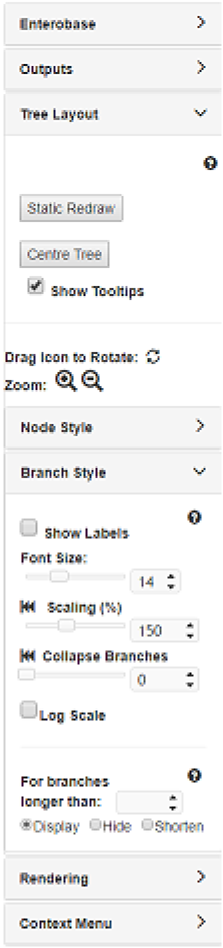

7. Styling the Branches

Under *Branch Style* we can also modify the look of the Branches in our Tree. We can show branch labels by checking the *Branch Labels* option and change the font size with the *Font Size* slider or by entering a new value in the box. If the

Mouseover info box is checked we can see the branch length when we have the mouse cursor over a particular branch.

The tree of the Agona dataset has very long branches. Enter 100 in the box next to *For branches longer than* and set it to 'Shorten'. This will shorten the branch length and change the line to be dashed, which indicates the branch length is not to scale.

8. Node settings

Under *Tree Layout* > *Node Style* we can modify the look of the Nodes in our Tree. We can show nodes labels by checking the *Node Labels* option and change the font size with the *Font Size* slider or by entering a new value in the box. If the *Mouseover info* box is checked we can see details for that node when we mouse over.

We can also set the colour coding of the Nodes. For the Agona dataset, Set *Colour by* to "Agona_Clusters" to show the outbreaks as defined the original paper. See if you can your figure to look like mine.

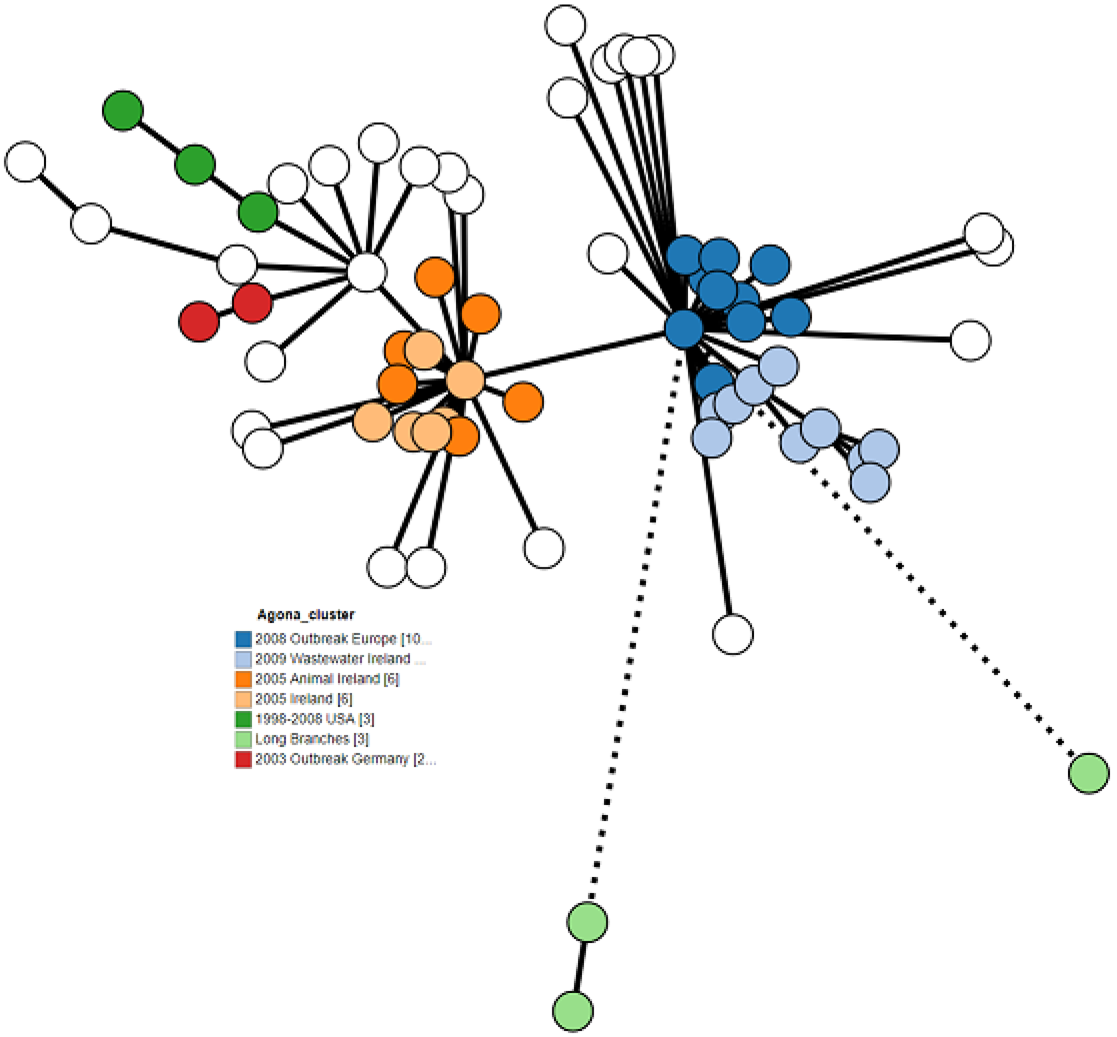

9. Final modifications

The tree is looking pretty good, but we can make it a bit clearer. Try playing around with all of the different options to come up with the best looking tree. Here's what I came up with:

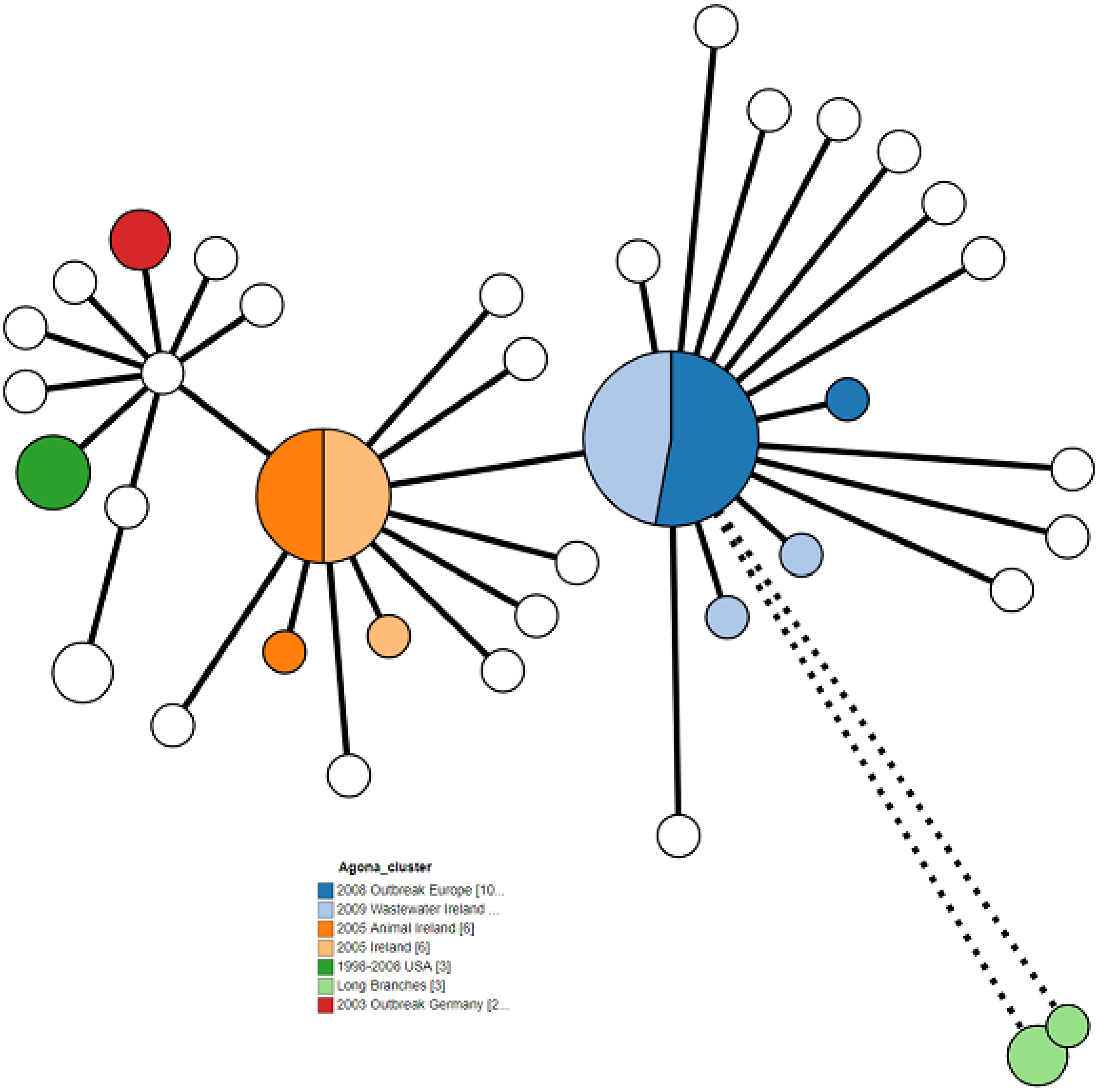

These are my settings:

- **Tree Layout > Branch Length**: 150%
- **Tree Layout > Collapse Branches**: 10
- **Branch Style > Shorten branches longer than**: 100
- **Node Style > Colour by:** Clusters
- **Node Style > Node Labels:** Unchecked/Off

10. Exporting our work

Your tree can be save in either GrapeTree's JSON format, as a Newick tree that can be loaded into other phylogeny programs and as a Scalar Vector Graphic (SVG), which is an image format that you can edit in publishing software like Inkscape or Adobe Illustrator. If you would like a raster image (JPEG, PNG, BMP) of the Tree, just use the screenshot feature of your computer.

1.7.4 Tutorial: Making your own GrapeTree Links

GrapeTree offers a choice to publish your interactive GrapeTree analysis online. This can be done by using two URL parameters.

Parameters:

- **tree** = <*online file for newick tree or json saved GrapeTree*>
- **metadata** = <*tab-delimited or comma-delimited table*>

Due to the CORS restrictions in jscript codes, only three sources have been tested as working:

- Files from the same domain as the GrapeTree server.
- Files in a GitHub public repository.
- DropBox files that have been publicly shared via links.

There are different ways of publishing data.

1. For normal users without a website.

You can publish your analysis online via GitHub preview version. For example:

- https://achtman-lab.github.io/GrapeTree/MSTree_holder.html?tree=https://raw.githubusercontent.com/achtman-lab/EnteroMSTree/master/examples/ebola.date.json

You can shorten these links by google URL shortener or bitly. i.e.:

- http://bit.ly/2H8py8F

2. Owners of websites.

Please run the standalone version of GrapeTree and serve the URL under the same domain as your main website. GrapeTree can read links from your website, as long as they were under the same domain.

You can either host the tree files in a different local link, or fetch them from external link at the backend and re-distribute them via a proxy link.

1.7.5 GrapeTree Reference Manual

This page explains each of the features in GrapeTree.

Interaction With Enterobase

- **Load Selected**: Any strains selected in the tree will be loaded into the main search page of Enterobase. If The main search page is not open, then a new page will open in the browser.
- **Highlight Checked**: Any strains checked (selected) in the Enterobase main search page will become highlighted (large yellow halo) in the tree.
- **Import Fields**: Shows a dialog box which allows the selection of experimental fields and custom fields (columns) to be imported into the tree.
- **Save**:Saves the tree layout and any metadata in the tree. Changed metadata is only associated with tree and will not be updated in Enterobase. Data in custom columns, however, which you have permission to edit, will be updated in Enterobase and you will be notified if this is the case.
- **Update**: Will update the tree with any metadata that has changed in Enterobase since the tree was created or the last update. Also any data from custom columns, which has changed in Enterobase will also be updated in ther tree.
- **Info**: Shows information about the tree such as the parameters used for construction, number of strains, last modified etc.

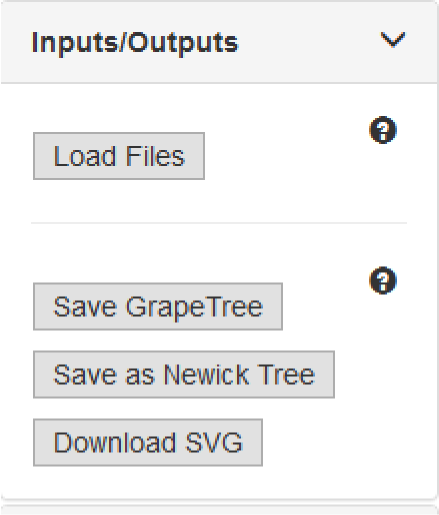

Loading data

To get started, Drag and drop files into the browser window

Trees or Profile Data

- **Phylogenetic trees**: These can be Nexus or Newick (nwk) format.
- **Profile Files**: These are tab delimited text with columns as alleles and strains as rows see *Tutorial 1B: Basic Usage of GrapeTree (Stand-alone)*. header line is required, in which the strain columns and metadata columns need to start with a '#'. This requires the local server to be running.
- **Custom Format(.json)**: Files generated by this program which contain the tree data exactly as displayed and any metadata.

Metadata

- **Metadata files** The first column in the file corresponds to the node identifier in the tree.

Saving in GrapeTree

- **Save GrapeTree**: Save the tree to a JSON format file, which contains the tree data exactly as displayed and any metadata. Only compatible with GrapeTree.
- **Save as Newick Tree**: Save the tree as a Newick (nwk) file, which contains tree topology, branch lengths and tip names. Compatible with most tree visualisation tools.
- **Download SVG**: Save the tree as a Scalar Vector Graphic (SVG) file, a vector image that can be loaded into image publishing software such as Inkscape or Abobe llustrator.

Modifying the Tree Layout

- **Original Tree**: Reverts the tree to the original state when it was loaded. You will lose all your changes!
- **Static redraw**: Redraws the tree using the static layout. You will lose any manual adjustments to node positioning!
- **Centre Tree**: Adjusts view settings to place entire tree in the centre of the window.
- **Show Tooltips**: Shows tooltips for branches and nodes.

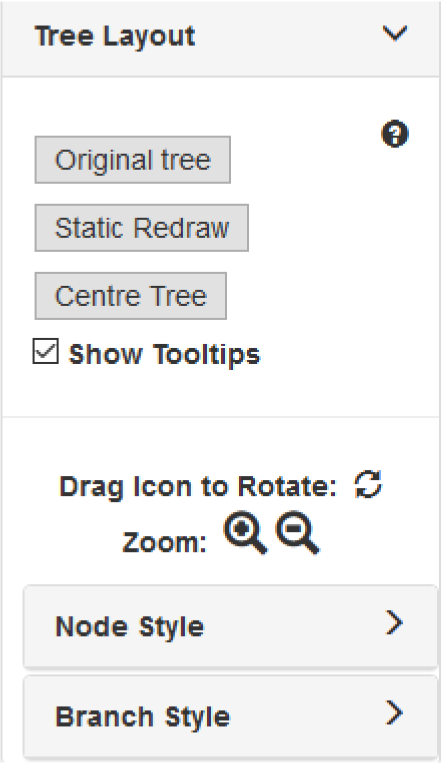

Node Style

- **Show Labels**: Check to show node labels and use dropdown to choose a label category.

- **Font Size**: Choose font size of node labels. Use the slider to change the value, or enter a specific value into the box
- **Node Size**: Increase/Decrease size of all nodes. Click rewind icon to revert to default value. Use the slider to change the value, or enter a specific value into the box
- **Kurtosis**: Increase/Decrease relative size of all nodes. Nodes with large number of members will look more distinct. Click rewind icon to revert to default value. Use the slider to change the value, or enter a specific value into the box
- **Show Pie Chart**: Shows breakdown of members contained within a node, categorized on "Colour by" setting

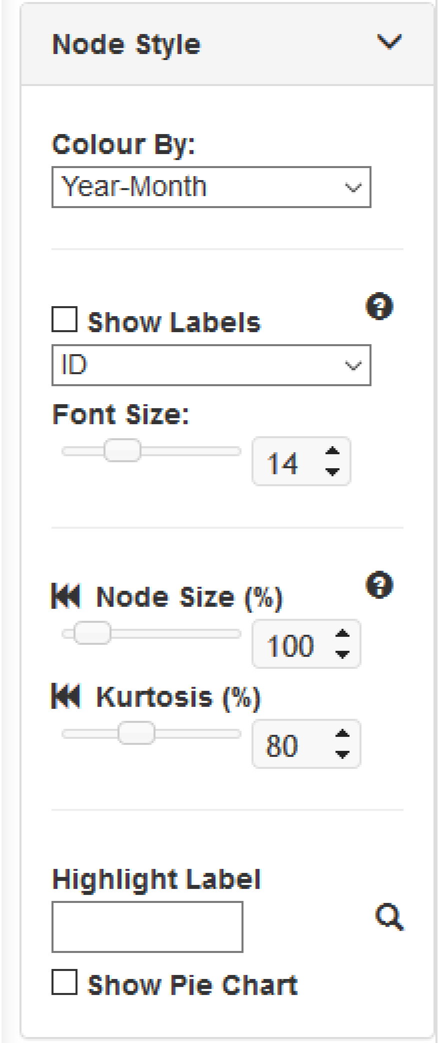

Branch Style

- **Show Labels**: Check to show node labels
- **Font Size**: Choose font size of node labels. Use the slider to change the value, or enter a specific value into the box.
- **Scaling**: Increase/Decrease length of all branches. Click rewind icon to revert to default value. Use the slider to change the value, or enter a specific value into the box.

- **Collapse Branches**: All branches shorter than specified length will be collapses and nodes will merged together. Branch length value is scaled to the branch lengths defined in the original tree data. Use the slider to change the value, or enter a specific value into the box.
- **Log Scale**: All length of all branches will be scaled logarithmically.

Branches that are over the specified length can be rendered in a particular way based on settings in this panel. Branch length value is scaled to the branch lengths defined in the original tree data. Enter a specific value into the box or use the arrows.

- **Display**: Long Branches will be show as normal
- **Hide**: Long branches will be transparent. They are interactive, but will not be shown on the tree.
- **Shorten**: Long branches will be cropped back to the specified branch length cutoff. Lines will be dashed to indicate affected branches.

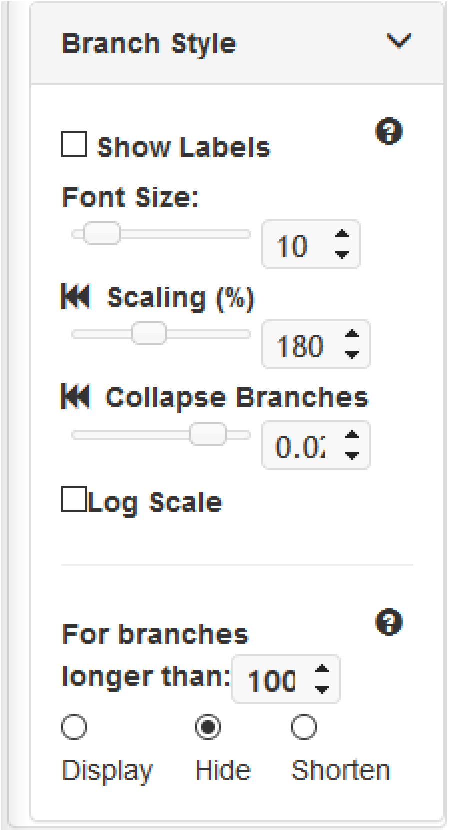

Layout Rendering options

Layout Rendering gives options on how nodes are positioned on the tree.

- **Dynamic**: Nodes are positioned dynamically similar to a Force Directed Layout. Nodes will try to fan out and distance themselves from neighbours. This may improve the aesthetic of the tree but will modify branch length scaling. Branch lengths are NOT to scale when this is used. The dynamic positioning can applied only to selected nodes if the "Selected Only" option is checked.
- **Static**: Tree layout is calculated when the tree is initially created and remains static. Relative branch length scaling (as specified in the original tree data) will be maintained if "Real Branch Length" option is checked.

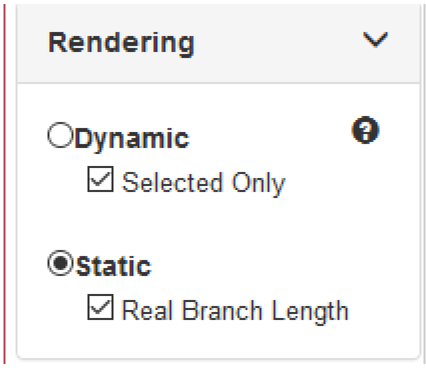

Context menu

Provides quick links to contextual menus, which are usually accessed by right click; this is for devices that do not have an easy right-click option such as tablets and mobile devices.

- **GrapeTree**: Presents the same menu as when right-clicking on the tree itself.
- **Metadata**: Presents the same menu as when right-clicking on the metadata table.
- **Figure Legend**: Presents the same menu as when right-clicking on the Legend.

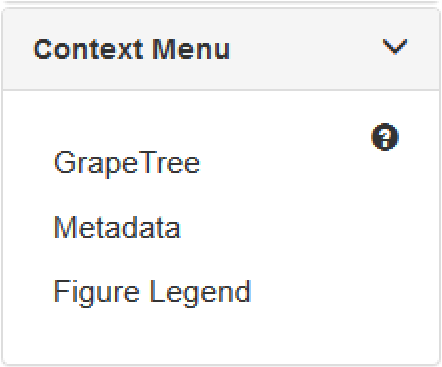

Metadata window

Provides a table that showing loaded metadata.

- **Download**: Export the metadata as a tab delimited file.
- **Add Metadata**: Click this to add a new column, specify the field name in the column.
- **Filter**: Shows filtering text boxes below each column header, when checked.
- **Hypo Nodes?**: Shows hypothetical nodes in the metadata table, when checked.

1.7.6 About GrapeTree

GrapeTree is named after the clusters of related bacterial strains that tends to be presented in minimal spanning trees. Our GrapeTree GUI is available within EnteroBase once you have created a workspace or connected to somebody else's workspace. fire It is also available here as a stand-alone version. The integrated EnteroBase version interacts directly with EnteroBase data. The stand alone version calculates trees from character data, visualizes pre-calculated trees and annotates them with information from supplied metadata.

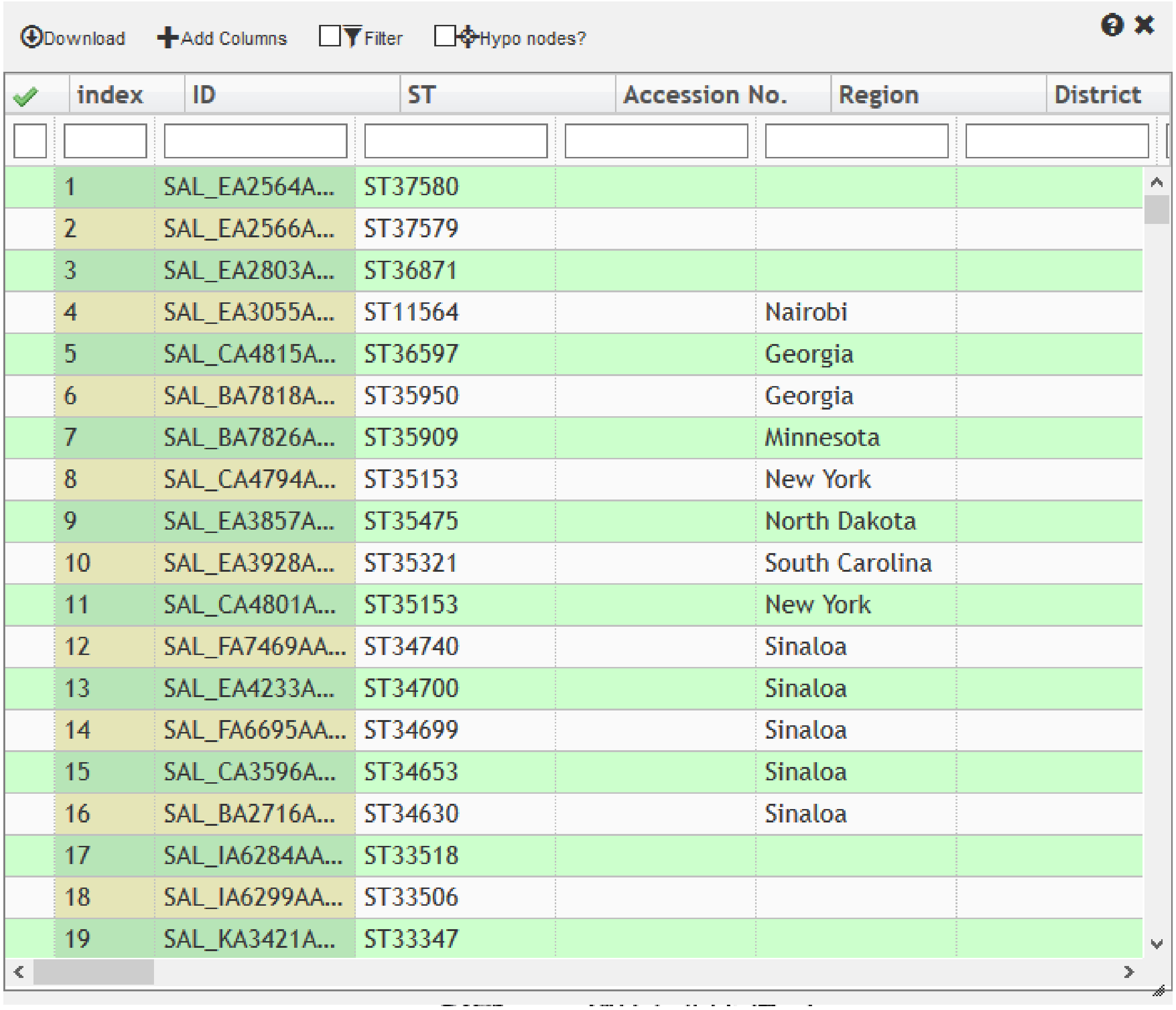

Download | Add Columns | Filter | Hypo nodes?

index	ID	ST	Accession No.	Region	District
1	SAL_EA2564A...	ST37580			
2	SAL_EA2566A...	ST37579			
3	SAL_EA2803A...	ST36871			
4	SAL_EA3055A...	ST11564		Nairobi	
5	SAL_CA4815A...	ST36597		Georgia	
6	SAL_BA7818A...	ST35950		Georgia	
7	SAL_BA7826A...	ST35909		Minnesota	
8	SAL_CA4794A...	ST35153		New York	
9	SAL_EA3857A...	ST35475		North Dakota	
10	SAL_EA3928A...	ST35321		South Carolina	
11	SAL_CA4801A...	ST35153		New York	
12	SAL_FA7469AA...	ST34740		Sinaloa	
13	SAL_EA4233A...	ST34700		Sinaloa	
14	SAL_FA6695AA...	ST34699		Sinaloa	
15	SAL_CA3596A...	ST34653		Sinaloa	
16	SAL_BA2716A...	ST34630		Sinaloa	
17	SAL_IA6284AA...	ST33518			
18	SAL_IA6299AA...	ST33506			
19	SAL_KA3421A...	ST33347			

GrapeTree aims to address two central issues:

- It is difficult to infer clusters from classical phylograms when showing large numbers of taxa.
- Tree construction methods based on Multi Locus Sequence Type (MLST) data (and extensions such as core genome MLST) do not correctly handle missing alleic information (See MSTreeV2 below).

GrapeTree is easy to integrate into existing web services. Since the release of our source code, Keith Jolley has written a GrapeTree wrapper specific for the BigsDB website/database environment, and thereby enabled GrapeTree functionality for all the databases served by PubMLST.

1.7.7 About MSTreeV2

MSTree V2 is a novel minimum spanning tree which is better suited for handling missing data than are classical MSTrees. First, a directed minimal spanning arborescence (dMST) (Edmond's algorithm) is calculated from asymmetric (directional) distances with tiebreaking of coequal branches based on allelic distances from a harmonic mean. Local branch recrafting is subsequently performed to eliminate the spurious branches that can arise within minimum spanning trees.

1.7.8 Citation

If you use GrapeTree please cite the preprint:

Z Zhou, NF Alikhan, MJ Sergeant, N Luhmann, C Vaz, AP Francisco, JA Carrico, M Achtman (2017) "GrapeTree: Visualization of core genomic relationships among 100,000 bacterial pathogens", bioRxiv 216788; doi: https://doi.org/10.1101/216788

1.8 Key features in Enterobase

1.8.1 Database homepage

From the main page, click on a database home link to be brought the dashboard for that database. This page provides a detailed overview of each genera and there are links to a number of tasks you can perform in EnteroBase.

- *Search strains* will allow you to query the database for records of interest (red).
- *Upload Reads* will allow you to upload your own sequence reads for analysis (green).
- *Show my Jobs* will show analysis jobs related to your data (blue).
- Manage your account through the dropdown in the top right.

These links on the side bar and along the top (grey) will always be present as you navigate deeper into the website so you can easily jump to another task.

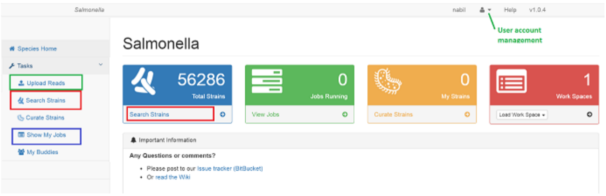

Supplement: Supplemental Material [file supp_gr.232397.117_Supplemental_data_S4.pdf]
