# Supplementary material for: GrapeTree: visualization of core genomic relationships among 100,000 bacterial pathogens
Source: Genome Res. 2018 Sep;28(9):1395–404. doi: 10.1101/gr.232397.117 (PMC6120633; doi:10.1101/gr.232397.117)
Supplement: Supplemental Material [file supp_gr.232397.117_Supplemental_data_S3.zip › Supplemental_data/GrapeTree-codes/static/js/SlickGrid/tests/40 grids on a page test.html]

SlickGrid example: Two Basic grids on page


|  |  |
| --- | --- |
| Grid 01   Grid 02   Grid 03   Grid 04   Grid 05   Grid 06   Grid 07   Grid 08   Grid 09   Grid 10   Grid 11   Grid 12   Grid 13   Grid 14   Grid 15   Grid 16   Grid 17   Grid 18   Grid 19   Grid 20   Grid 21   Grid 22   Grid 23   Grid 24   Grid 25   Grid 26   Grid 27   Grid 28   Grid 29   Grid 30   Grid 31   Grid 32   Grid 33   Grid 34   Grid 35   Grid 36   Grid 37   Grid 38   Grid 39   Grid 40   Demonstrates:   - two basic grids with minimal configuration   View Source:   - View the source for this example on Github | |
