# Supplementary material for: GrapeTree: visualization of core genomic relationships among 100,000 bacterial pathogens
Source: Genome Res. 2018 Sep;28(9):1395–404. doi: 10.1101/gr.232397.117 (PMC6120633; doi:10.1101/gr.232397.117)
Supplement: Supplemental Material [file supp_gr.232397.117_Supplemental_data_S3.zip › Supplemental_data/GrapeTree-codes/static/js/SlickGrid/tests/test-4-rows-paging.html]

SlickGrid example 4: Model


SlickGrid

**Search:**


---

Show tasks with % at least: 

  
And title including:

  
  
Select first 10 rows
Set Page Size to 4
  

## Demonstrates:

- a filtered Model (DataView) as a data source instead of a simple array
- grid reacting to model events (onRowCountChanged, onRowsChanged)
- **FAST** DataView recalculation and **real-time** grid updating in response to data changes.  
  The grid holds **50'000** rows, yet you are able to sort, filter, scroll, navigate and edit as if it had 50
  rows.
- adding new rows, bidirectional sorting
- column options: cannotTriggerInsert
- events: onCellChange, onAddNewRow, onKeyDown, onSelectedRowsChanged, onSort
- NOTE: all filters are immediately applied to new/edited rows
- Handling row selection against model changes.
- Paging.
- inline filter panel

## View Source:

- View the source for this example on Github

Show tasks with title including 
and % at least
