# Supplementary material for: GrapeTree: visualization of core genomic relationships among 100,000 bacterial pathogens
Source: Genome Res. 2018 Sep;28(9):1395–404. doi: 10.1101/gr.232397.117 (PMC6120633; doi:10.1101/gr.232397.117)
Supplement: Supplemental Material [file supp_gr.232397.117_Supplemental_data_S3.zip › Supplemental_data/GrapeTree-codes/static/js/SlickGrid/tests/plugins/autotooltips.html]

SlickGrid - AutoTooltips plugin tests


# SlickGrid - AutoTooltips Test Suite
